# Impacts of feeding less food-competing feedstuffs to livestock on global food system sustainability

**DOI:** 10.1098/rsif.2015.0891

**Published:** 2015-12-06

**Authors:** Christian Schader, Adrian Muller, Nadia El-Hage Scialabba, Judith Hecht, Anne Isensee, Karl-Heinz Erb, Pete Smith, Harinder P. S. Makkar, Peter Klocke, Florian Leiber, Patrizia Schwegler, Matthias Stolze, Urs Niggli

**Affiliations:** 1 Research Institute of Organic Agriculture (FiBL), Ackerstrasse 113, 5070 Frick, Switzerland; 2 Institute of Environmental Decisions, ETH Zürich, Universitätstrasse 22, 8092 Zürich, Switzerland; 3 Food and Agriculture Organization of the United Nations (FAO), Viale Terme di Caracalla, 00150 Rome, Italy; 4 Institute of Social Ecology Vienna (SEC), Alpen-Adria University Klagenfurt-Vienna-Graz, Schottenfeldgasse 29, 1070 Vienna, Austria; 5 Scottish Food Security Alliance-Crops and Institute of Biological and Environmental Sciences, University of Aberdeen, 23 St Machar Drive, Aberdeen AB24 3UU, UK; 6 Bovicare GmbH, Hermannswerder Haus 14, 14473 Potsdam, Germany

**Keywords:** food security, livestock, sufficiency, consistency, sustainable intensification, food system

## Abstract

Increasing efficiency in livestock production and reducing the share of animal products in human consumption are two strategies to curb the adverse environmental impacts of the livestock sector. Here, we explore the room for sustainable livestock production by modelling the impacts and constraints of a third strategy in which livestock feed components that compete with direct human food crop production are reduced. Thus, in the outmost scenario, animals are fed only from grassland and by-products from food production. We show that this strategy could provide sufficient food (equal amounts of human-digestible energy and a similar protein/calorie ratio as in the reference scenario for 2050) and reduce environmental impacts compared with the reference scenario (in the most extreme case of zero human-edible concentrate feed: greenhouse gas emissions −18%; arable land occupation −26%, N-surplus −46%; P-surplus −40%; non-renewable energy use −36%, pesticide use intensity −22%, freshwater use −21%, soil erosion potential −12%). These results occur despite the fact that environmental efficiency of livestock production is reduced compared with the reference scenario, which is the consequence of the grassland-based feed for ruminants and the less optimal feeding rations based on by-products for non-ruminants. This apparent contradiction results from considerable reductions of animal products in human diets (protein intake *per capita* from livestock products reduced by 71%). We show that such a strategy focusing on feed components which do not compete with direct human food consumption offers a viable complement to strategies focusing on increased efficiency in production or reduced shares of animal products in consumption.

## 1. Background

Since the 1960s, breeding efforts to improve genetic potential, improvements in herd management, increase in use of protein- and energy-rich concentrate feed and a reduction in use of low-productivity grassland systems have increased the productivity of livestock systems [1]. This led to an increase in feed conversion efficiency, per-animal yields and labour productivity, and a decrease in greenhouse gas (GHG) emissions per kg of animal product [2].

However, the livestock sector as a whole has considerably grown in absolute terms and contributes substantially to global warming, water and air pollution and biodiversity loss [1,3,4]. This overall growth of livestock production parallels population growth and increasing *per capita* incomes that are associated with increasing shares of animal products in human diets [5].

About one-third of arable land is currently used for feed production [1,6,7] and about a third of global cereal production is fed to animals [8]. This leads to considerable trade-offs with producing food for direct human consumption as food provision via animals entails large conversion losses [9–12]. The proportion of arable land used for livestock feed production is expected to increase further, thus increasing the pressure on arable land areas [8].

Several strategies to increase sustainability in livestock production have been suggested. They largely fall into three categories.

(1) Productivity increases, aiming at meeting expected demand while curbing environmental impacts (‘efficiency strategies' [13]): they include improved feeding and feed use efficiency, improved digestibility, protein and mineral contents, optimally matching the animals' requirements, breeding and herd management [2]. They contribute to the sustainable intensification of agriculture [14,15] and provide many benefits for society. For example, if applied globally, GHG emissions from the livestock sector could be reduced by 30% when compared with a reference without such intensification [16].
(2) Reduced demand for animal products (‘sufficiency strategies'): they include changes in human diets and demand patterns, but also measures such as the replacement of ruminants' products with monogastrics' products [9,17,18]. Changes in dietary patterns can have considerable mitigation potential, as demonstrated by several modelling studies [19–21]. A comprehensive overview of the literature, distinguishing between supply and demand-side measures, can be found in [21].
(3) Reduction of the use of food-competing feed components in livestock rations, which also affects the availability of livestock products (a ‘consistency strategy’ [22] or ‘transformation of the food system’ [15]): this consistency strategy shifts the focus from livestock's role in the food system as a source for high-quality protein, to another role, which is to use resources that cannot otherwise be used for food production. These resources are (a) grasslands, which cover two-third of global agricultural area and can be used for food production by ruminants, whereas a large proportion of these grasslands is not or less suitable for arable crop production [23–25] and (b) food waste and by-products of food production–consumption chains, such as brans, whey and oil-cakes [26,27]. The rationale is that environmental pressures from livestock production could be reduced by focusing on grassland-based ruminant production and by reducing the amount of primary feedstuffs derived from cropland in both ruminant and monogastric feeding rations [3,7,20,28]. This affects production and consumption at the same time as it would also lead to a reduction in animal product supply.

While the impacts of the efficiency and sufficiency strategies have been modelled in detail in previous works [9,16,18,29,30], the consistency strategy of reducing food-competing feedstuffs (FCF) in livestock rations has not previously been assessed to this extent.

In this paper, we explore the potential for sustainable livestock production by modelling the impacts of such a consistency strategy on food provision as well as on natural processes. We scrutinize the potential and challenges of reductions in FCF and investigate the implications of such a consistency strategy as one option for sustainable livestock production.

It has to be pointed out that the consistency strategy that we analyse in this paper is a complement and not a substitute of the sufficiency and efficiency strategies. It restricts the feeding rations for livestock and thus limits the availability of livestock products for human consumption. Corresponding changes in consumption patterns are thus one important implication of this strategy.

We use a mass-flow model of the food system to investigate the effects of the consistency strategy of reducing FCF on crop and livestock production patterns, human dietary patterns and key environmental indicators. This study examines the implications of such a strategy from a physical and biological perspective, aiming at maximal coverage regarding country-wise production and availability of final and intermediate commodities and related nutrient requirements and availability, as well as environmental impacts. It explicitly does not aim at assessing price changes and market effects and the decision behaviour of farmers and consumers. The purpose of this study is, instead, to examine the system-level food and environmental implications of pursuing this consistency strategy and to identify whether it could be a complement to efficiency and sufficiency strategies.

## 2. Methods

This analysis employs a bottom-up mass-flow model of the agricultural and food sector, described in the following and the electronic supplementary material. The model uses FAOSTAT [6] as the central data source and covers 180 plant production activities (e.g. cultivating 1 ha of wheat for a year) and 22 livestock production activities (e.g. keeping a dairy cow for a year). The base year refers to mean values for the years 2005–2009. These are the most recent data available that are compatible with the other datasets used, with 192 single countries and territories as geographical reference units.

Country-specific herd structures for cattle, pigs and chickens were estimated to improve calculations of feed requirements and GHG emissions. Herd structures were calculated for each country with an optimization model using a cross-entropy estimator. These models predict the most likely average herd structure in a country based on the relation between producing and living animals according to FAOSTAT as well as a number of normative data (see electronic supplementary material, §1.3.2).

For each activity, we defined inputs and outputs, i.e. all physical flows related to individual activities. Inputs for livestock activities include four categories of livestock feeds: (i) fodder crops grown on arable land, i.e. according to FAO, land being cropped or fallow, (ii) concentrate feed derived from human-edible food (e.g. grains, pulses) grown on arable land, (iii) grassland-based fodder, and (iv) fodder from agricultural/agri-industrial by-products. While (i) and (ii) are in competition with production of human-edible food, (iii) and (iv) are not. The term grasslands is used synonymously with the term grazing land. Further inputs for livestock activities are energy input for buildings, in-stall processes and fences. Outputs of animal production activities include human-edible and human-inedible products, manure excretion, nutrient losses and GHG emissions owing to enteric fermentation and manure management (CH_4_, N_2_O, NO_3_ and NH_3_). Country-specific data for amounts of concentrate feed and by-products used are derived from FAOSTAT food balance sheets (see electronic supplementary material, §1.3.7). Inputs for plant production activities included arable or grassland areas, mineral fertilizers, manure, crop residues, symbiotic nitrogen fixation, herbicides, fungicides, insecticides and management practices. Outputs from plant production activities include crop yield quantities, crop residues and nitrogen losses during fertilizer application. Based on these data, we calculated livestock feed and fertilizer supply/demand balances at national, regional and global level.

The main model outputs are food availability (equation (2.1)) and environmental impacts (equation (2.2)).2.1
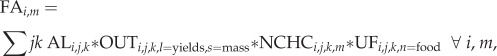
where *i* is the index of geographical units, *j* is the index of activities, *k* is the index of farming systems, *l* is the index of inputs and outputs, *m* is the index of nutrients for human consumption, *n* is the index of utilization types (food, feed, seed, waste, other) and *s* is the index of units of inputs and outputs. FA is the food availability expressed in kcal or g protein, AL is the activity level (ha per year for land-use activities, number of animals per year for livestock activities), OUT is the output (kg per ha or kg per animal), NCHC is the nutrient contents for human consumption [%] and UF is the utilization factor [%].

In the electronic supplementary material, we describe how food availability per person, activity levels, inputs and outputs, nutrient contents and utilization factors are determined in our model.

### 2.1. Modelling environmental impacts

Environmental impacts are aggregated across all geographical units, activities and farming systems (equation (2.2)). Activity levels (AL*_i,j,k_*) are multiplied by inputs (IN*_i,j,k,s,o_*) and the impact factors of the inputs (IF*_i,j,k,l,s,o_*).2.2
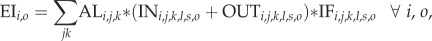
where EI is an environmental impact, *o* is the index of environmental impacts, IN = inputs [kg or ha] and IF = impact factors [environmental impact per kg of input or output per emission]. An overview of the environmental indicators used in this study and their units are given in table 1. In the main body of the paper, we focus on land occupation, N-surplus, GHG emissions and deforestation, whereas the other indicators (P-surplus, renewable energy use, pesticide use, freshwater use, soil erosion) are addressed only shortly. Further methodological details on the main indicators and more detailed results on the other indicators are provided in the electronic supplementary material, §1.3.10.

**Table 1.** Overview of the indicators for analysing environmental impacts in the model.

environmental impact	indicator	unit
land occupation	land occupation by arable and grassland	ha
soil erosion potential	crop-specific factor covering the erosion susceptibility of crops combined with country-specific or regional average soil erosion rates	t soil lost per year
non-renewable energy demand	cumulative energy demand, versions 1.05–1.08	GJ per year
greenhouse gas emissions	global warming potential (GWP) IPCC100a	t CO_2_-eq per year
nitrogen surplus	nitrogen surplus	N-surplus per ha per year
phosphorus surplus	P_2_O_5_ surplus	P_2_O_5_-surplus per ha per year
pesticide use	classification of pesticide use per ha by intensity and by crop, legislation by country and access to pesticides by farmers	semi-quantitative indicator
annual deforestation potential	additional crop land required annually	ha per year
water use	use of water for irrigation	m^3^

#### 2.1.1. Land occupation

This indicator measures how much land is necessary for agricultural production each year. Because arable land is much scarcer and more valuable than permanent grasslands for food production, we differentiate between land occupation of arable land and grassland. For equation (2.2), the inputs (IN) that are taken into account are grassland and arable land. For all arable crops and grasslands, the IF is defined as one. This indicator combines values for areas harvested with values for cropping intensities that indicate how often, on average, a hectare is harvested per year. On average, cropping intensity is less than one; therefore, land occupation is larger than the values for areas harvested [6,8].

#### 2.1.2. N-surplus

NO_3_ losses to soil, and NH_3_ and N_2_O losses to the atmosphere occur as a result of N use in agricultural systems. Consequently, sensitive terrestrial and aquatic ecosystems are adversely affected.

N-surplus is defined as the difference between the N content of outputs (e.g. yields) and inputs (e.g. fertilizer quantities) for each country and activity. Changes in cropping areas, animal numbers (manure), production quantities, mineral fertilizer use and N-fixation thus potentially lead to changes in N-surplus. Based on equation (2.2), the amount of N is calculated by multiplying the mass of an input (IN) or output (OUT) by its N content. Relevant inputs for calculating the N-surplus are mineral N fertilizers, N-fixation, organic fertilizer, crop residues and seeds. Relevant outputs are yields and crop residues. IF is defined as the N-content of the inputs, whereas all outputs are defined as negative values. As a basis for calculating GHG emissions, N-losses during fertilizer application are separated according to the type of fertilizer (mineral, manure, crop residues) and the substance emitted (NH_3_, NO_3_, N_2_O). Model factors are specified according to IPCC 2006 Guidelines (Tier 1). Model calculations for the total N-balance in the base year are in line with literature values reported for different sources and the overall balance [1,31,32]. We did not include estimates of atmospheric nitrogen deposition in the N-surplus calculations.

#### 2.1.3. Greenhouse gas emissions

GHG emissions of the agricultural sector have been estimated by several projects at regional [28] or global level [33–36]. Estimations of global GHG emissions of the agricultural sector are between 4.2 and 5.2 Gt CO_2_-eq [21] and this constitutes approximately 10–12% of total global emissions.

GHG emissions were modelled according to the Global Warming Potential (GWP) ‘IPCC 2006 100a’ tier 1 methodology [37]. For enteric fermentation modelling, we used the tier 2 methodology in order to capture the impacts of different feeding regimes on GHG emissions. Additionally, the GWP owing to the production of inputs from non-agricultural sectors (mineral fertilizers and pesticides) was included in calculations according to LCA studies [38,39], the ecoinvent 2.0 database and [40]. To calculate the GHG emissions from processes and buildings, the cumulative energy demand (CED) values for different processes were taken from ecoinvent 2.0 and transformed into GWP values with process-specific conversion factors derived from ecoinvent 2.0. Emissions from deforestation and from organic soils under agricultural use were taken directly from [41]. According to equation (2.2), all relevant inputs (e.g. fertilizers) and processes (e.g. enteric fermentation) were specified in physical quantities. The respective CO_2_-eq values of CO_2_, CH_4_ (25) and N_2_O (298) were used as IF, as suggested in the IPCC 2006 guidelines. Restricting the analysis to the common emission categories, total GHG emissions calculated for the base year in our model are similar to [16,41]. These references only differ substantially in terms of enteric fermentation calculations; the results of our model are similar to [41].

#### 2.1.4. Annual deforestation potential

Because agricultural land is scarce and natural grasslands are generally not well suited for cultivation (water or temperature limited), increasing the amount of land needed for agricultural production increases pressure on grasslands and forests [42]. Conversion of grassland to cropland may also indirectly lead to increased deforestation, owing to displacement effects that result in the conversion of forests to meadows and pastures [43,44]. With limited data available, we have assumed that additional cropland generally increases pressure on forests and may lead to increased deforestation. Following Kissinger *et al*. [45], we have attributed 80% of deforestation to agriculture. Following Alexandratos & Bruinsma [8], we have forecast constant grassland areas.

The deforestation potential of agricultural land expansion was estimated from the average annual growth in agricultural area and the average annual deforestation rates in each country from 2005 to 2009 (taken from FAOSTAT). Deforestation rates in the scenarios were calculated by multiplying the change in land areas in each scenario by the ratio of deforestation areas over agricultural land area expansion, scaled by a factor of 0.8 to account for the 80% of deforestation attributed to agriculture.

In cases where no change in agricultural land area was reported for the years 2005–2009, deforestation values were calculated using the *total* agricultural area (instead of the change in agricultural area) as a proxy for the pressure of agriculture on forests. In these cases, deforestation rates were calculated by multiplying the total agricultural land area in each scenario by the ratio of deforestation areas from [41] over total agricultural land area in the base years, scaled by the factor 0.8. The indicators for deforestation were applied only in the cases of positive deforestation rates. Deforestation was set to zero in countries where total forest area increased.

#### 2.1.5. Other indicators

Here, we provide short descriptions only, further details can be found in the electronic supplementary material, §1.3.9. *P-surplus* is calculated analogously to the N-surplus. All P-flows are expressed as P_2_O_5_. No differentiation between types of P-losses is made. Therefore, the balance (inputs–outputs) calculated expresses a ‘loss potential’, acknowledging that large quantities of P are fixed in soils. The total P-balance in the base year as calculated in our model is in line with literature values reported in [31]. *Non-renewable energy use* is calculated according to the life cycle impact assessment methodology, ‘CED’ [40]. Only the non-renewable energy categories (fossil and nuclear energy) are used, and renewable energy components are disregarded. Inventory data for each activity were taken from the ecoinvent 2.0 database and [41–44]. *Water use* was derived based on AQUASTAT [46] data for irrigation use per tonne of irrigated production and data on irrigated areas for various crops and crop categories covered in [13]. As there is no consistent dataset on *pesticide use* covering different countries, we developed an impact assessment model for assessing pesticide use incorporating three factors: pesticide use intensity per crop and farming system, pesticide legislation in a country, and access to pesticides by farmers in a country (for details, see electronic supplementary material, §1.3.9.4). *Soil erosion potentials* were derived based on an assessment of soil erosion susceptibility per crop and soil erosion rates per country (literature review and expert judgements, details in electronic supplementary material*,* §1.3.9.5).

### 2.2. Scenarios

We calculated a reference scenario based on the most recent FAO projections for agricultural production patterns and food production and demand in 2050 [8], and a range of scenarios with a gradual reduction of FCF ranging from the reference scenario (referred to as 100% FCF) to 0% FCF. Each scenario presented provides the same amount of *per capita* energy as the reference scenario as the main measure of food availability. Additional scenarios, for constant *per capita* protein supply and for constant land use are given in the electronic supplementary material, §2. By-products from food production (brans, oilseed cake, whey, etc.) are assumed to be fed to animals in each scenario (electronic supplementary material, §1.3.5). Livestock numbers were derived from per-animal feed requirements and the available feed supply in each scenario. Land no longer required to supply animal feed was allocated to plant food production, according to the mix of crops in the reference scenario until the global levels of energy or protein for human consumption match the requirements of the reference scenario. For making the scenarios more comparable, grassland areas were kept at the level of the reference scenario [8]. Yields per animal were assumed to drop with reduced FCF. To account for the uncertainties regarding this effect, we computed the uncertainty range of 0–40% yield decrease with such feed pattern changes (electronic supplementary material, §1.4.3). The values presented in the paper refer to the mid-value of 20% yield reduction. Values for the boundary cases (0% and 40%) are presented in the electronic supplementary material, §2. Fish and seafood also decreased with a reduction of FCF, as such feed is used in aquaculture (assuming fed aquaculture to comprise about 20% of fish and seafood in the current situation, about 45% in the reference scenario [47,48], electronic supplementary material, §1.4.1.6). For the scenario with 0% food-competing feedstuffs (0%FCF), the induced reductions in animal protein supply were compensated by adjusting the share of legumes in cropping patterns to at least 20%, by allocating larger shares of the areas freed from feed production to legumes (electronic supplementary material, §2). This allows keeping the share of energy delivered through protein at recommended levels of at least 10% also without animal products. Average crop rotations were thus assumed to include a legume crop once every 5 years. This is also feasible agronomically, e.g. regarding breaking disease cycles in legumes. The effect of climate change on yields was assessed by means of sensitivity analysis based on the references and details given in electronic supplementary material, §1.4.3, covering a range from zero yield increases under strong climate change impacts to yield increases as reported in [8], signifying no climate change impact.

## 3. Results

**Figure 1.**
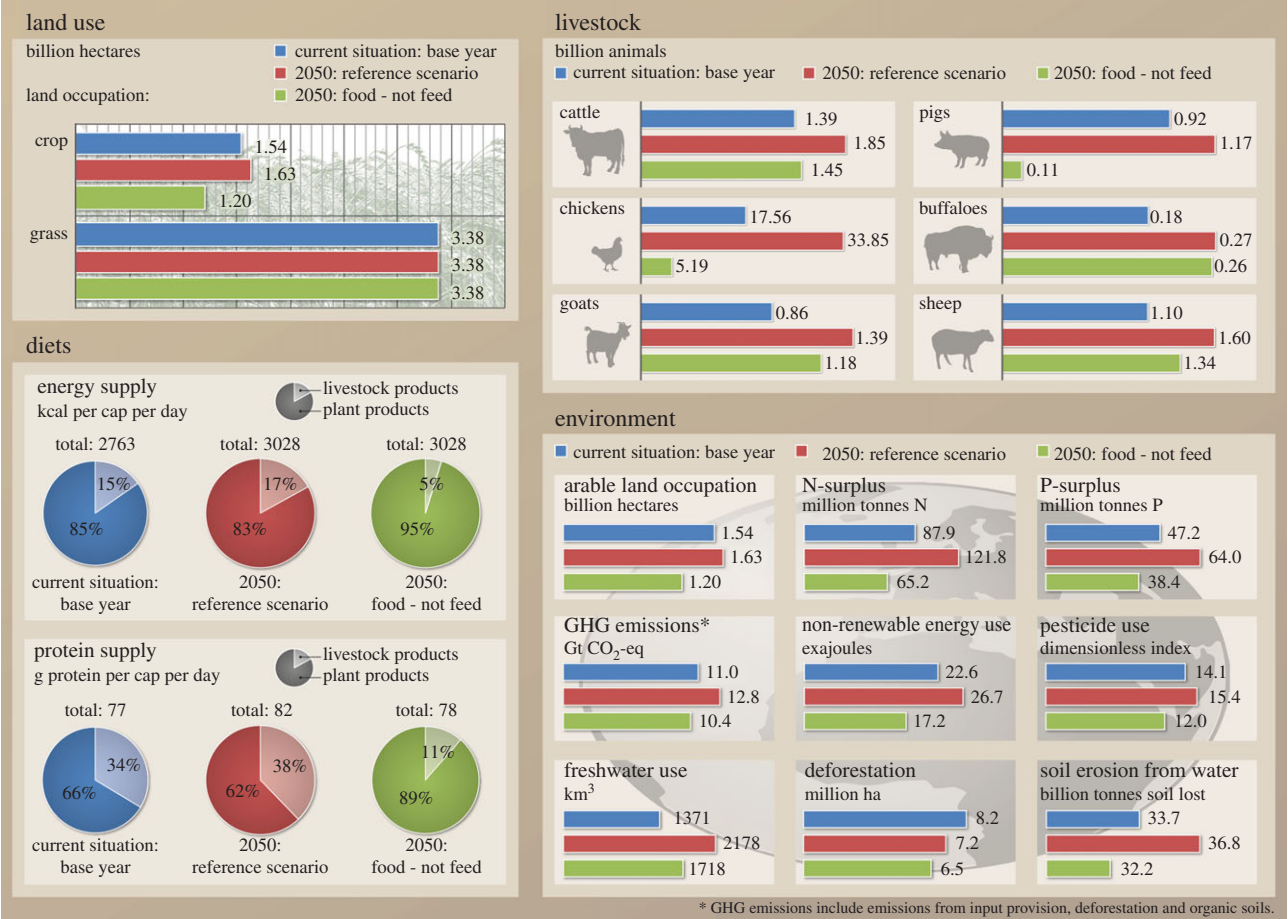
Impacts of feeding less food-competing feedstuffs to livestock (‘food - not feed’) on land use, livestock numbers, human diets and the environment in 2050.

**Figure 2.**
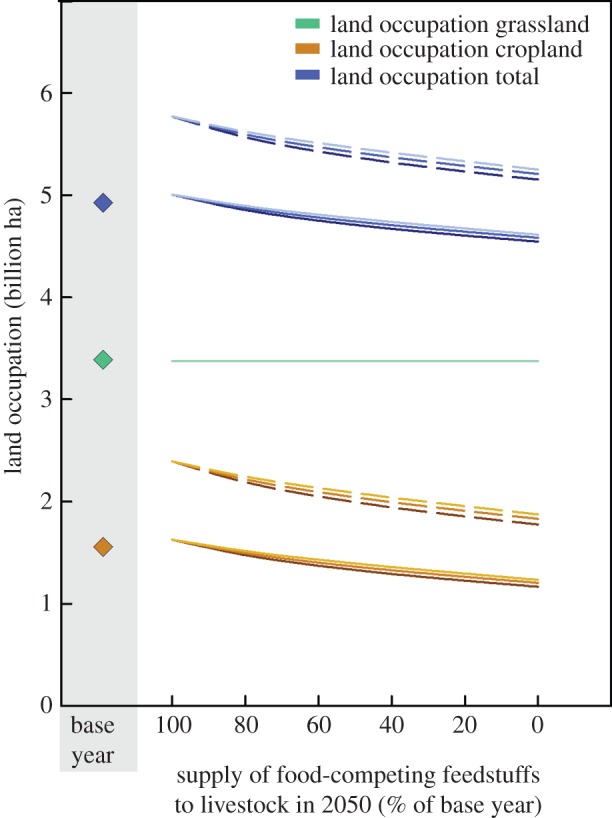
Land occupation by cropland, grassland and total agricultural land in the base year, reference scenario, i.e. no reduction in food-competing feedstuffs (=100%) and with reduced usage of such feedstuffs. Diamonds (filled diamonds): levels in the base year. Solid lines: negative impact of climate change (CC) on yields absent; dashed lines: CC impact present. Sensitivity to livestock yield reductions owing to reduction of food-competing feedstuffs: 0% (dark-coloured lines), 20% (medium-coloured), 40% (light-coloured).

**Figure 3.**
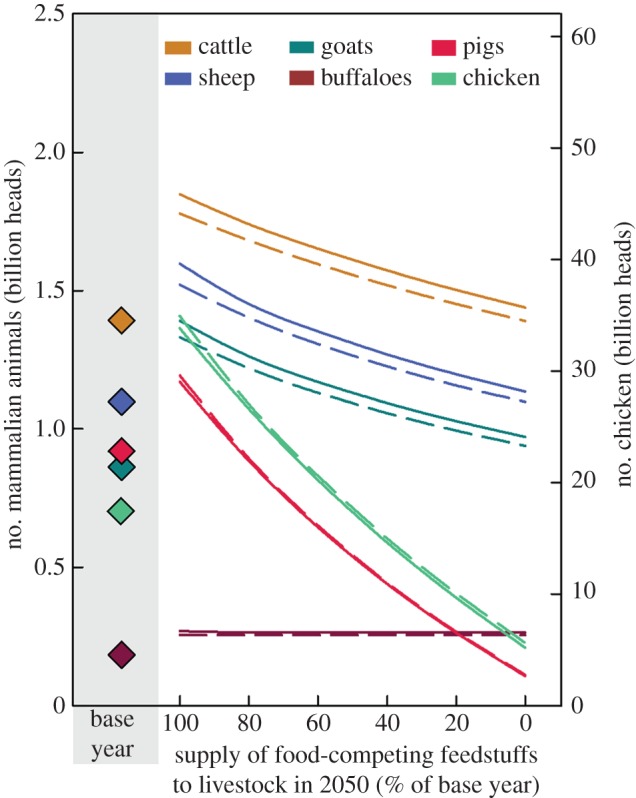
Livestock numbers in the base year, reference scenario, i.e. no reduction in food-competing feedstuffs (=100%) and with reduced usage of such feedstuffs. Diamonds (filled diamonds): levels in the base year. Solid lines: negative impact of climate change (CC) on yields absent; dashed lines: CC impact present.

**Figure 4.**
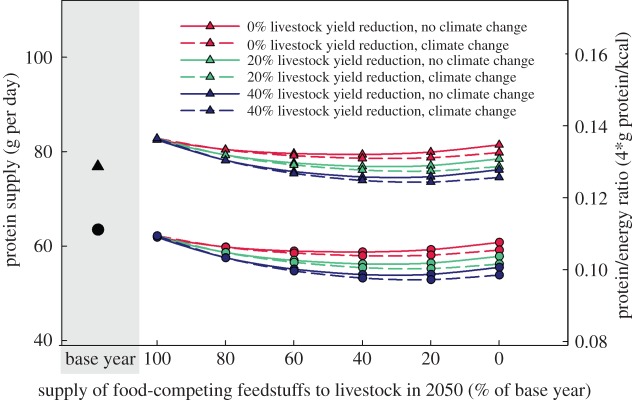
Daily protein supply per person [g protein per person per day] and protein/calorie ratio in the base year, the reference scenario for 2050 and with reduction of food-competing feedstuffs (global averages). Filled triangles, protein supply; filled circles, protein/energy ratio. Black symbols: base year.

**Figure 5.**
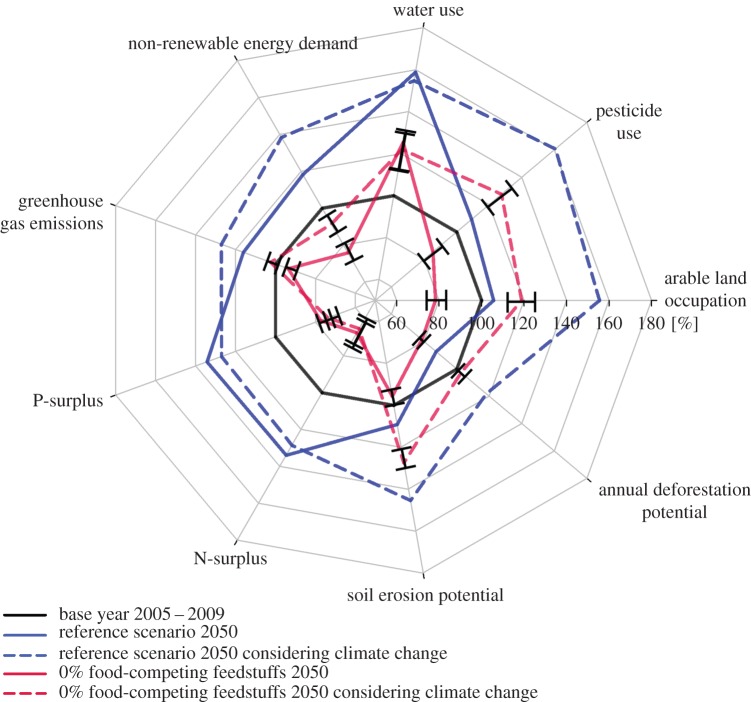
Change of environmental pressures resulting from a reduction in food-competing feedstuffs relative to the base year [%]. Solid lines: negative impact of CC on yields absent; dashed lines: CC impact present. Black: base year; blue: reference scenario (same level of food-competing feedstuffs use assumed for 2050); red: 0% food-competing feedstuffs. Black whiskers: range from 0% to 40% animal yield reduction.

Figure 1 gives an overview of the results comparing the base year (BAS), reference scenario (REF) and the scenario with 0%FCF. The other figures provide further details with regard to the impacts of a partial switch towards less FCF (figures 2–4) and sensitivity analyses (figures 2–4 and figure 5).

### 3.1. Changes in agricultural production patterns

In the reference scenario for 2050 [8], grassland area is assumed to stay constant compared with the current situation (base year), whereas arable land is projected to increase from 1.54 to 1.63 Mha, i.e. by 6% (figures 1 and 2), resulting in a 2% increase in total agricultural land area. In the reference scenario, animal numbers are projected to increase from 1.39 to 1.85 billion animals for cattle (33% increase), from 0.9 to 1.2 billion animals for pigs (27% increase) and from 17.6 to 33.9 billion animals for chickens (by 93%) if compared with the base year (figures 1 and 3).

Compared with the base year, the scenario with 100% reduction of FCF resulted in a 335 Mha decrease in arable land area, which corresponds to a decrease of 22% in arable and 7% in the total agricultural area. For cattle, in the scenario with 0%FCF, the number would increase by 60 million, i.e. 4% compared with the base year, and goat, sheep and buffalo numbers would increase by 320, 240 and 80 million, respectively (i.e. 37%, 22% and 44%), as these animals are mainly fed on grasslands and are thus less dependent on feed sources that compete with direct food production. In the 0%FCF scenario, the number of monogastrics is substantially reduced by 12.37 billion (i.e. 70%) for chickens and 810 million (88%) for pigs (figures 1 and 3).

Depending on the extent to which climate change limits the growth of crop yields (electronic supplementary material, §1.4.3), cropland area would need to increase by up to 0.85 Mha, i.e. 55%, in the reference scenario compared with the base year. In the 0%FCF scenario, these increases in cropland area are limited to 0.29 Mha (19%) for the worst-case scenario, showing a considerable reduction in pressure on land use from this scenario, particularly if projected crop yield increases cannot be achieved (figure 2).

### 3.2. Changes in food consumption patterns

Food consumption patterns are represented via projected provision in quantities, calories and proteins *per capita* and day (table 2), differentiated by commodity group (see electronic supplementary material, §1.3.8). We report food supply *before* subtraction of food waste at retail and consumption level. For the production level, the quantities of food loss reported in FAOSTAT have been used in order to be consistent with Alexandratos & Bruinsma [8].

**Table 2.** Daily intake of main food categories per person (fresh matter, primary crop equivalents, global average) in the base year, the reference scenario and in scenarios with reduced food-competing feedstuffs (no climate change impacts on yields, 20% yield reduction in livestock due to reduction in food-competing feedstuffs use, cf. Methods).

			supply of food-competing feedstuffs to livestock in scenarios for 2050 [% of base year]							
food types (PPE)^a^	unit^b^	base year (2005–2009)	100%	80%	60%	40%	20%	0%	difference of 0% food-competing feedstuffs scenario to base year (%)	difference of 0% to 100% food-competing feedstuffs scenario (%)
plant products	g/(cap*day)	1442	1484	1495	1507	1512	1509	1499	4	1
grains	g/(cap*day)	519	499	531	555	570	577	575	11	15
starchy roots	g/(cap*day)	185	193	201	207	212	214	212	15	10
oil crops	g/(cap*day)	74	104	96	90	84	79	73	−1	−30
legumes	g/(cap*day)	42	52	69	89	112	140	177	317	242
vegetables	g/(cap*day)	343	295	278	263	248	231	213	−38	−28
fruits	g/(cap*day)	210	260	243	228	215	201	187	−11	−28
sugars and sweeteners^c^	g/(cap*day)	65	78	73	70	66	63	60	−8	−23
others^d^	g/(cap*day)	5	4	4	4	4	3	3	−39	−29
livestock products	g/(cap*day)	425	484	400	336	283	239	201	−53	−58
milk	g/(cap*day)	242	274	237	207	181	158	138	−43	−50
meat	g/(cap*day)	110	136	101	75	54	38	26	−77	−81
non-ruminants meat	g/(cap*day)	77	97	68	46	29	16	7	−91	−93
ruminants meat	g/(cap*day)	34	39	33	29	25	22	19	−43	−50
fish	g/(cap*day)	50	48	44	41	39	37	35	−30	−27
eggs	g/(cap*day)	23	26	19	13	8	5	2	−90	−91
all products	g/(cap*day)	1867	1968	1896	1843	1794	1747	1701	−9	−14
total energy availability	kcal/(cap*day)	2763	3028	3028	3028	3028	3028	3028	10	0
total protein availability	g CP/(cap*day)^e^	77	82	79	78	77	77	78	1	−5
animal protein/total protein (%)	ratio	34	38	31	24	19	15	11	−67	−70
energy from proteins/total energy	ratio	0.111	0.108	0.104	0.103	0.102	0.102	0.103	−8	−5

^a^PPE, primary product equivalents.

^b^Cap, person.

^c^Raw sugar equivalents.

^d^Mainly treenuts, stimulants and spices.

^e^CP, crude protein.

To allow for optimal comparison with the reference scenario, *per capita* calorie supply from both plants and animals in the scenarios was kept constant at the level of the reference scenario (3028 kcal cap^−1^ d^−1^). This slightly differs from the 3070 kcal cap^−1^ d^−1^ reported in [8] owing to some differences in assumptions for cases where we had access to newer information, or where underlying information from [8] has not been available. This high number of calorie availability includes food wastage of about 30–40% on global average, which when deducted leads to a level in the range of human maintenance requirements. In the scenario with 0%FCF and at the same time keeping energy levels in human diet constant, the share of energy delivered through protein would change from 10.8% to 10.3% owing to the higher share of crops in the human diet, and crops generally having lower protein relative to energy contents (figures 1 and 4).

Owing to the decreasing animal numbers and livestock yields, the share of livestock products in the total protein supply would drop from 38% to 11% and the share of livestock products in the total energy supply would drop from 17% to 5% (with 20% livestock yield reduction; figures 1 and 4). This is also reflected in the *per capita* daily consumption quantities of different commodity groups. Meat, eggs and milk drop from 136, 26 and 274 g cap^−1^ day^−1^ to 26, 2 and 138 g cap^−1^ day^−1^, respectively. Climate change (i.e. lower yield increases) leads to further small changes in dietary composition with less livestock products and more grains, legumes and fish.

### 3.3. Environmental impacts

We focus on the presentation of the results on N-surplus, GHG emissions and deforestation. Results on land occupation have been covered already above. Results for the other impacts (P-surplus, non-renewable energy use, water use, pesticide use and soil erosion) are included in figures 1 and 5 and discussed shortly; more details can be found in the electronic supplementary material, §2.1. Details for the calculations are provided in the Methods section and in particular in the electronic supplementary material, §1.3.

In the reference scenario, all environmental impacts are exacerbated compared with the base year, except for deforestation rates (figures 1 and 5). The N-surplus (i.e. total input minus total extraction by crops per ha; global average, including grasslands) increases by 34%, which means an increase from 18.6 to 25.0 kg ha^−1^ yr^−1^. This is driven by the increase in output from the whole food system, which leads to correspondingly increased input use, i.e. mineral fertilizer inputs and N-fixation (as legume areas and production increase as well), whereas the increases in agricultural area are much lower. GHG emissions increase by 27%. This again reflects the increase in production volume; increased emissions from higher ruminant numbers and manure quantities as well as increased fertilizer inputs to the fields are the main drivers of these emission increases. With deforestation and organic soils included, the increase in GHG emissions in comparison with the base year is 16%, which reflects the lower changes in those two additional categories in comparison with the agricultural production. Deforestation pressure decreases by 13% compared with the base year. The decrease in deforestation rates is due to the reduced expansion rates in agricultural area between now and 2050 compared with the expansion rate in the base years 2005–2009. The lower expansion rates of agricultural land are due to assumptions about yield increase and cropping intensity increase in the reference scenario [8]. Those effects, and not the utilization of additional land, are the main mechanisms through which increased food demand would be met. For the other environmental impacts, most notably, freshwater use increases by about 60%, owing to an increase in irrigated areas and irrigation intensity. Pesticide use and erosion potential increase by about 10% each, driven by the increase in arable land areas, and P-surplus and non-renewable energy demand increase by 30% and 20%, driven by the general increase in production volumes and corresponding input use.

For the 0%FCF scenario, the environmental impacts are lower than in the reference scenario just described (figures 1 and 5). Compared with the current situation, the N-surplus per ha would drop by 22%, as the whole production volume and corresponding demand for inputs is decreased. GHG emissions would increase by 1%, or would drop by 5% by including deforestation and organic soils. This is due to a drastic reduction in animal numbers and manure quantities, as well as in total N-fertilizer quantities needed. It is important to point out that owing to the focus on grassland feed, the number of ruminants is reduced much less than the number of monogastrics (figures 1 and 3), and that the effect of reduced emissions from enteric fermentation is thus less prominent than it would be in a strategy that would predominantly aim at reducing ruminants to reduce emissions from enteric fermentation. We also note that we did not include atmospheric N-deposition in the calculations. Given that animal husbandry and mineral fertilizers account for a large share of NH_3_ emissions as the key source for N-deposition [49], we thus rather underestimate how the reduction of FCF affects the N-surplus, as these sources are also correspondingly lower. Deforestation pressure is reduced by 21% compared with the base year, which reflects the reduced land demand already reported above. The other environmental impacts besides water use are lower than in the base year, driven by the reduced production volumes, animal numbers and cropland areas. Freshwater use still increases by 25% owing to the increase in the share of irrigated areas (figures 1 and 5).

How environmental impacts change as a result of climate change effects on yields is also displayed in figure 5. Generally, the environmental impacts in the 0%FCF scenario are still smaller than in the reference scenario, but the relative advantages decrease if climate change impacts are included (electronic supplementary material*,* §1.4).

## 4. Discussion and conclusions

### 4.1. Creating synergies between enhanced food availability and reduced environmental impact

A continuation of current food consumption and production trends, as forecast in Alexandratos & Bruinsma [8], increases *per capita* food availability until 2050. However, food availability in that scenario hinges on large yield increases over the next 40 years, with environmental impacts projected to increase substantially. If projections of climate change effects and natural limitations on yields are considered, then agricultural land areas would have to increase drastically to meet the forecast demand for 2050 (figure 2).

Livestock production with lower shares of FCF would generate synergies between increased food availability and reduced environmental impacts. Our exploration of the impacts of a consistency strategy with 0%FCF shows that reduction in land use and emissions can be realized, albeit with significant changes in people's diets, as well as changes of the role of livestock. It would avoid drastic increases in the demand for agricultural land area, in particular if more pessimistic yield forecasts under climate change transpire.

The results of our study are not to be understood as forecasts but as explorations of possible long-term futures. It is important to note that the results of this study are subject to uncertainties, stemming from known data flaws or lacking data, particularly for smaller countries and developing countries. Therefore, extrapolation of some datasets is unavoidable, and uncertainties of future trends that are not included in the model, for instance the share of renewable energies in country-specific energy mixes, demand for biofuels or potential new technologies such as cultured meat, evolve. However, because we use the model at global level and model only fundamental changes in food systems, the general trends of our results are meaningful, as shown in the uncertainty analysis (see electronic supplementary material). Such an exploration of possible long-term futures is required, as fundamental changes in the food system will not be feasible within the timeframe of only one decade.

### 4.2. Implications of the strategy with reduced food-competing feedstuffs for livestock production

Advocating reduced grass-based production of ruminants and enhanced use of concentrates, which contain human-edible feedstuffs, for both ruminants and monogastrics is not the only strategy to achieve sustainable intensification. Here, we show that a consistency strategy which reduces FCF is a viable alternative. Such a strategy could combine the advantages of breeding, veterinary health measures and feed management, with a strategy that aims at reducing the amount of cropland-derived feedstuffs, to thus alleviate land-use competition [50].

Ruminants have been the focus of sustainability discussions because of the large CH_4_ emissions from enteric fermentation [1,3]. Roughage-fed ruminants could, however, play an important role for food security, as they allow the use of resources that are otherwise not, or only barely, usable for food production, as is the case with most global grasslands [23]. Therefore, in the scenarios with 0%FCF, the number of monogastrics is reduced much more than the number of ruminants, and roughage-fed ruminants still provide an important source of protein. We show that a food system with ruminant- and grassland-based animal products can provide enough food while reducing environmental impacts. Furthermore, grasslands can contain large carbon stocks and can provide many ecosystem functions [24]: much of which would be lost if grassland were converted to arable land [51–53]. An important challenge to the livestock feed industry will be to further improve the use of agricultural residues, agro-industrial by-products and waste material to produce high-quality feedstuffs [54,55], where reuse is a far better option than landfilling, incineration, composting or anaerobic digestion.

### 4.3. From modelling production systems to modelling food systems

While most studies concentrate either on production issues [16] or consumption patterns [15,30], this assessment emphasizes the importance of considering the nexus between agricultural production patterns and systems with food consumption. Thus, it links the discussion of sustainable food production and sustainable food consumption and can be used to assess integrative strategies that have an impact on both resource efficiency of production and the availability of certain foodstuffs. We show that despite roughage-fed beef or milk having a higher carbon footprint than products from intensive, concentrate-fed cattle systems, or even pig and poultry, the scenario with 0%FCF results in a more sustainable food system than the reference scenario based on business-as-usual projections, as losses in resource efficiency are more than offset by the beneficial effects of reducing feed production on arable land. This perspective of connecting efficiency and consumption strategies can complement existing life cycle assessments and economic modelling approaches [56].

The scenarios we have investigated would necessitate dietary change; namely reduced consumption of animal products, with particular reductions in pig and poultry meat, and eggs. This is viable from a physical and food availability point of view and would also yield other benefits, primarily related to human health [57]. High consumption of livestock products has been linked to non-communicable and chronic diseases, and obesity [29]. The societal acceptability of such dietary change is not well understood, but is clearly key to any successful implementation of such a strategy [19], and likely remains challenging [58].

While other studies examining the impacts of changing food consumption patterns concentrated on the reduction of ruminant production or on livestock products in general, this study provides insights into the relative benefits of roughage-fed meat and milk over other livestock products from the perspective of sustainable consumption. We have shown that in such a scenario, the reduction in consumption of monogastric livestock products would be much more drastic than for ruminant meat. Thus, there are alternatives to the frequently suggested replacement of ruminant with monogastric meat, which is based on carbon footprints or attributional life cycle assessments of single products that do not consider the limited availability of arable land and the utilization of grasslands.

Our scenarios are based on nutrient balances and assessments of the physical and technical viability of different food production scenarios and global food system scenarios that have not previously been captured in global land-use models. This provides important insights concerning the physical viability and environmental effects of these food system scenarios. However, to assure food security, access to food, stability and utilization also need to be addressed in addition to food availability [14].

Reducing the amount of human-edible crops that are fed to livestock represents a reversal of the current trend of steep increases in livestock production, and especially of monogastrics, so would require drastic changes in production and consumption. Achieving such drastic changes is a huge challenge for society. Policy measures on both the supply and demand sides would be required to assist such structural change necessary to prevent potential future crises for food availability, the environment and human health [15,50]. Long-term and global *ex ante* impact assessments, such as that presented here, are essential to inform the scientific debate and to provide a basis for informed decision-making.

Clearly, to decide on specific policy measures and implementation options for these strategies, physical models that assess the principal viability and impacts need to be complemented with economic models to take market effects on demand and supply into account [59]. Such economic assessment is, however, beyond the scope of this study.

Ideally, elements of all proposed strategies may best be combined to achieve sustainable food systems, complementing increased efficiency with reduced meat consumption and changed livestock feeding patterns towards less human-edible crops and feed from arable land. Such a combination would avoid the need to pursue one strategy to very high levels of implementation, that are likely expensive and unrealistic, but a combination of strategies, each implemented at intermediate levels may be promising. The contribution of this paper is to show that a consistency strategy with 0% FCF can play a significant role in such a combination of complementary strategies, on par with the other previous suggestions.

## Supplementary Material

Supplementary Information

## Data accessibility

All data and modelling code are accessible as .gms and .gdx files at ftp://paper.fibl.ch (username: Paper; password: +pAp!er-2).
