# Supplementary material for: Impacts of feeding less food-competing feedstuffs to livestock on global food system sustainability
Source: J R Soc Interface. 2015 Dec 6;12(113):20150891. doi: 10.1098/rsif.2015.0891 (PMC4707862; doi:10.1098/rsif.2015.0891)
Supplement: Supplementary Information [file rsif20150891supp1.pdf]

Impacts of feeding less food-competing feedstuffs to livestock on global food system sustainability

SUPPORTING INFORMATION

Christian Schader[1*], Adrian Muller[1,2], Nadia El-Hage Scialabba[3], Judith Hecht[1], Anne Isensee[1], Karl-Heinz Erb[4], Pete Smith[5], Harinder P.S. Makkar[3], Peter Klocke[1,6], Florian Leiber[1], Patrizia Schwegler[2], Matthias Stolze[1], Urs Niggli[1].

Affiliations:

[1] Research Institute of Organic Agriculture (FiBL), Ackerstrasse 113, 5070 Frick, Switzerland

[2] Institute of Environmental Decisions, ETH Zürich, Universitätstrasse 16, 8092 Zürich, Switzerland

[3] Food and Agriculture Organization of the United Nations (FAO), Viale Terme di Caracalla, 00150 Rome, Italy

[4] Institute of Social Ecology Vienna (SEC), Alpen-Adria University Klagenfurt-Vienna-Graz, Schottenfeldgasse 29, 1070 Vienna, Austria

[5] Scottish Food Security Alliance-Crops and Institute of Biological and Environmental Sciences, University of Aberdeen, 23 St Machar Drive, Aberdeen, AB24 3UU, Scotland, UK

[6] bovicare GmbH, Hermannswerder Haus 14, 14473 Potsdam, Germany

*Correspondence to: christian.schader@fibl.org.

Contents

1 Modelling food systems at global level ... 3
1.1 Fertilizer balance ... 3
1.2 Animal feed balance ... 3
1.3 Modelling the food system in the base year ... 4
1.3.1 Activity levels ... 4
1.3.2 Herd and flock structures ... 4
1.3.3 Inputs for plant production activities ... 13
1.3.4 Outputs of plant production activities ... 14
1.3.5 Inputs for livestock production activities ... 15
1.3.6 Outputs of livestock production ... 15
1.3.7 Modelling utilization of agricultural products ... 16
1.3.8 Modelling food demand ... 17
1.3.9 Additional information on environmental impacts ... 17
1.4 Modelling the food sector in the scenarios ... 19
1.4.1 Reference scenario ... 19
1.4.2 Scenarios with reduction in food-competing feedstuffs ... 24
1.4.3 Sensitivity analysis ... 24
2 Results additional to those shown in the main paper ... 26
2.1 Results for the additional environmental impacts. ... 26
2.2 Additional results for scenarios with constant calorie provision in all scenarios for 2050 ... 26
2.3 Results for scenarios with constant protein provision in all scenarios for 2050 ... 36
2.4 Results for scenarios with constant land use in all scenarios for 2050 ... 50
Appendices ... 64

1 Modelling food systems at global level

In this section supplementary information on the model is given. Information on how fertilizer and feed balances were calculated is given in Section 1.1 and 1.2. Afterwards we explain the detailed assumptions and data used to model the food sector in the base year (Section 1.3) and present the adjustments undertaken in the scenarios (Section 1.4).

1.1 Fertilizer balance

Fertilizer demand was calculated using Equation 6, which gives the sum of nutrient demand for each crop by multiplying crop and grassland activity levels by yields and nutrient demand, where $PND_{r,j,k,q}$ is the nutrient demand per tonne of each product (index r).

$$FERTD_{i,q} = \sum_{j,k} AL_{i,j,k} * OUT_{i,j,k,Yield} * PND_{r,j,k,q} \; \forall \; i, q \qquad (6)$$

Where:

q = index of plan nutrients (subset of index s);

FERTD = Fertilizer demand [kg of nutrient];

FERTI = Fertilizer input [kg of nutrient];

PND = Product-specific nutrient demand [kg nutrient / tonne of product harvested].

Fertilizer input is calculated using Equation 7, which gives the sum of all N and P inputs for crop production over activities, farming systems and all inputs and outputs that are relevant for N and P (mineral fertilizers, organic manure, crop residues and N fixation). Sections 1.3.2 to 1.3.6 describe how the inputs, outputs and nutrient contents (NC) for the fertilizer balance are calculated.

$$FERTI_{i,q} = \sum_{j,k,l} AL_{i,j,k} * IN_{i,j,k,l,Mass,q} * NC_{i,j,k,q} + \sum_{j,k,l} AL_{i,j,k} * OUT_{i,j,k,l,Mass,q} * NC_{i,j,k,q} \; \forall \; j, k, l \; (7)$$

Where:

NC = nutrient content in the inputs and outputs [%].

Different types of fertilizers are allocated as follows. For mineral fertilizers, country-specific fertilizer use [1] was related to total nutrient demand. For the scenarios, it was assumed that mineral fertilizers are applied with the same ratio of supply to demand. Crop residues and manure are applied proportionally to the remaining N-demand after accounting for mineral N fertilizer applications, with a share of 50% of manure remaining on grasslands. This procedure determines the amounts of crop residues and manure applied and the corresponding phosphorus inputs. N-fixation is a fertilizer input to legumes only, as the effect for other crops is represented by in crop residue inputs from leguminous crops. The total amount of crop residues and manure is determined at national level and distributed to all crops as described above.

1.2 Animal feed balance

Animal feed demand is calculated using Equation 9, which gives the sum of the nutrient demand for each livestock type by multiplying livestock activity levels by animal feed demand factors for grass-based fodder, animal feed from forage crops and concentrate feed. Animal feed demand factors were specified according to a number of references and datasets [2-6]. For cattle, pigs and chickens, separate herd structure models are used, allowing for a more exact definition of demand factors (see Section 1.3.2).

$$FEEDD_{i,t,u} = \sum_{j,k} AL_{i,j,k} * IN_{i,j,k,t,u} \; \forall \; i, t, u \qquad (9)$$

Where:

t = index of animal nutrition requirement (subset of index s);

u = index of animal feed types;

FEEDD = Animal feed demand [kg of nutrient].

Animal feed supply is calculated using Equation 10, which gives the sum of all outputs of all crop and livestock activities multiplied by the share of the products utilized as animal feed and the product's digestible energy and raw protein content. Feed nutrient contents are taken from several data sources [7-11]. The derivation of utilization factors is described in Section 1.3.7.1.

$$FEEDS_{i,t,u} = \sum_{j,k,l} AL_{i,j,k} * OUT_{i,j,k,Yield,Mass,t} * UF_{i,j,k,feed} * NC_{i,j,k,t} \; \forall \; i,t,u \quad (10)$$

Where:

FEEDS = Feed input [kg of nutrient].

1.3 Modelling the food system in the base year

The base year (the scenario named "current situation") was modelled as an average of FAOSTAT data from the period 2005-2009 in order to smooth annual variations in yields and prices. As a priority, FAOSTAT data were used as far as possible for the base year in SOL-m. Data gaps were filled with supplementary data if FAOSTAT data were either missing, or of poor quality. If several data sources were available, supplementary data was selected based on the principles of quality and comprehensiveness.

1.3.1 Activity levels

To model activity levels for the current situation, areas harvested were taken from FAOSTAT (average of the years 2005-2009) for each country.

The number of live animals in the base year is taken from stock numbers in the FAOSTAT data. This number does not account for imports and exports. It is assumed that this stock number is the average number of all living animals in one country. The number of animals in the reference scenario is taken from Alexandratos and Bruinsma [12].

1.3.2 Herd and flock structures

1.3.2.1 General description

Herd structures describe the composition of an animal herd in terms of different sub-classes defined according to age, sex and production purpose of the animal (Table S1). Herd structures were calculated for pigs, chickens and cattle based on assumptions relating to fertility rates, age of first calving, slaughtering rates and losses due to diseases and accidents (Table S2), calibrated using FAOSTAT data for producing animals, living animals (stocks), imports and exports. Herd structures were calculated for each country with a separate optimization model using a cross entropy estimator [13] (Equation 10). These models predict the most likely average herd structure in a country, based on information that is available. Support points 1, 2 and 3 were defined based on expert opinions. While support point 2 describes a central value, support points 1 and 3 refer to the upper and lower bounds of a country-specific parameter.

$$H(p,q) = -\sum_X p(x) * \log q(x) \quad (10)$$

Where:

H (p,q) is the entropy;

P (x) = true distribution;

q (x) = given probability distribution.

For each sub-class of livestock (Table S1), feeding requirements and GHG emissions were defined based on IPCC [14] and Kirchgeßner [15]. Feeding requirements and GHG emission categories for a country-specific average category of animal livestock were calculated, as each sub-class has different feeding needs. Average feeding requirements for each livestock main type (cattle, chickens and pigs) were calculated using a) the composition of the herds and flocks in each country as calculated by the herd structure models and b) the feeding requirements defined for each livestock sub-class.

The per-head average was multiplied by the number of living animals to give the total feeding needs of the animals in a country. For other animal types, we calculated feeding requirements using a live-weight ratio with the most similar animal (cattle, pigs or chickens).

Table S1 Overview of livestock types defined in cattle, pig and chicken herd structure models

Herd	Sub-class	Description
Cattle	A_Dcow	Number of dairy cows
Cattle	A_Dsire	Number of dairy sires
Cattle	A_DFemaleCalf1	Number of female dairy calves aged 1 year
Cattle	A_DFemaleCalf2	Number of female dairy calves aged 2 years
Cattle	A_DFemaleCalf3	Number of female dairy calves aged 3 years
Cattle	A_DMaleCalf1	Number of male dairy calves aged 1 year
Cattle	A_DMaleCalf2	Number of male dairy calves aged 2 years
Cattle	B_Bcow	Number of beef cows
Cattle	B_Bsire	Number of beef sires
Cattle	B_BFemaleCalf1	Number of female beef calves aged 1 year
Cattle	B_BFemaleCalf2	Number of female beef calves aged 2 years
Cattle	B_BMaleCalf1	Number of male beef calves aged 1 year
Cattle	B_BMaleCalf2	Number of male beef calves aged 2 years
Pigs	A_Sows	Number of sows
Pigs	A_Boars	Number of boars
Pigs	A_Sucklers	Number of sucklers
Pigs	A_Weaners	Number of weaners
Pigs	A_Fatteners	Number of fatteners
Chickens	A_Lbhen	Number of laying hens
Chickens	A_Lbcock	Number of laying hen cocks
Chickens	A_Lrearchick	Number of rearing chicken
Chickens	A_Lgrowchick	Number of growing chicken
Chickens	B_Fbhen	Number of breeding hens
Chickens	B_Fbcock	Number of breeding hen cocks
Chickens	B_Ffatteningchick	Number of fattening chicken

Table S2 Overview of external variables of the cattle, pig and chicken herd structure models

Herd	Variable	Support point 1	Support point 2	Support point 3
Cattle	Share of calve losses in year 1	0.01	0.10	0.50
Cattle	Share of calve losses in year 2	0.01	0.10	0.50
Cattle	Share of calve losses in year 3	0.01	0.10	0.50
Cattle	Fertility rate of dairy cows	0.50	0.90	1.00
Cattle	Fertility rates of beef cows	0.50	0.80	1.00
Cattle	Calving rates	0.55	0.95	1.00
Cattle	Share of slaughtered male dairy calves aged 1 year	0.00	0.05	0.10
Cattle	Share of slaughtered female dairy calves aged 1 year	0.00	0.01	0.10
Cattle	Share of slaughtered female dairy calves aged 2 years	0.00	0.05	0.10
Cattle	Share of slaughtered male beef calves aged 1 year	0.00	0.05	0.10
Cattle	Share of slaughtered female beef calves aged 1 year	0.00	0.05	0.10
Cattle	Share of sires	0.0095	0.01	0.0105
Cattle	Dairy cow replacement rate	0.10	0.30	0.50
Cattle	Beef cow replacement rate	0.10	0.30	0.50
Cattle	Dairy sire replacement rate	0.10	0.30	0.50
Cattle	Beef sire replacement rate	0.10	0.30	0.50
Cattle	Age at first calving (years)	2.00	2.50	4.00
Pigs	Share of suckler losses	0.01	0.12	0.40
Pigs	Share of weaner losses	0.01	0.05	0.20
Pigs	Share of fattener losses	0.01	0.03	0.10
Pigs	Suckling period (days)	7.00	28.00	45.00
Pigs	Weaning period (days)	20.00	35.00	60.00
Pigs	Fattening period (days)	80.00	120.00	250.00
Pigs	Litters per year	1.00	1.50	2.50
Pigs	Litter size	8.00	13.00	15.00
Pigs	Share of boars	0.01	0.08	0.10
Pigs	Culling rate of sows	0.20	0.40	0.50
Pigs	Culling rate of boars	0.25	0.40	0.50
Pigs	Age at first parturition (months)	12.00	15.00	18.00
Chickens	Share of rearing losses	0.01	0.02	1.00

Herd	Variable	Support point 1	Support point 2	Support point 3
Chickens	Share of growing losses	0.01	0.02	1.00
Chickens	Share of laying losses	0.01	0.05	1.00
Chickens	Share of fattening losses	0.01	0.03	1.00
Chickens	Rearing period (days)	100.00	140.00	200.00
Chickens	Growing period (days)	70.00	85.00	100.00
Chickens	Laying period (days)	170.00	252.00	300.00
Chickens	Fattening period (days)	28.00	39.00	50.00
Chickens	Turnover	5.00	8.00	9.00
Chickens	Share of slaughtered fattening chicken	0.70	0.90	1.00
Chickens	Share of cocks	0.00	0.01	0.01
Chickens	Female breeding hens	40.00	80.00	85.00
Chickens	Male breeding hens	60.00	120.00	135.00
Chickens	Eggs per hen	150	280.00	365.00

All herd and flock structures are based on a virtual starting population of each age and sex category (marked in Figures 2-6 with the abbreviation ST). After one year the starting population is modified by: a) subtracting young animal losses (LO), animal slaughtering (SL), young animals entering the adult population (XC), and exports (EXP); and b) adding replacement animals (REP) and imports (IMP), to give an end of year population. The final number of animals in each category refers to the arithmetic mean of the start (ST) and end population numbers (EN). The end population of each cohort serves as the starting population for the next year (e.g. the end population of one year old animals in the first year serves as the starting population of two year old animals in the second year). For pigs and chickens of different age and sex categories, we calibrated the herds and flocks to one year. Average numbers (AV) are multiplied by the number of life cycles per year. For cattle, we defined a dairy and a beef herd structure, and for chickens we defined a laying hen and fattening chicken flock. Each herd and flock structure is linked through several conditions (purple boxes) which state that the total number slaughtered, living, imported and exported must equal the observed quantities in FAOSTAT. For laying hens and fattening chickens, FAOSTAT also provides the total population numbers.

Figures 2-6 describe the herd and flock structures and dynamics for dairy cattle, beef cattle, pigs, laying hens and fattening chickens. All herd and flock structure figures should be read from left to right for each age and sex category. The dairy and beef herd structures are similar, except for the absence of third year female beef calves (Figure 3). Some support points also differ between dairy and beef cattle (see Table 4).

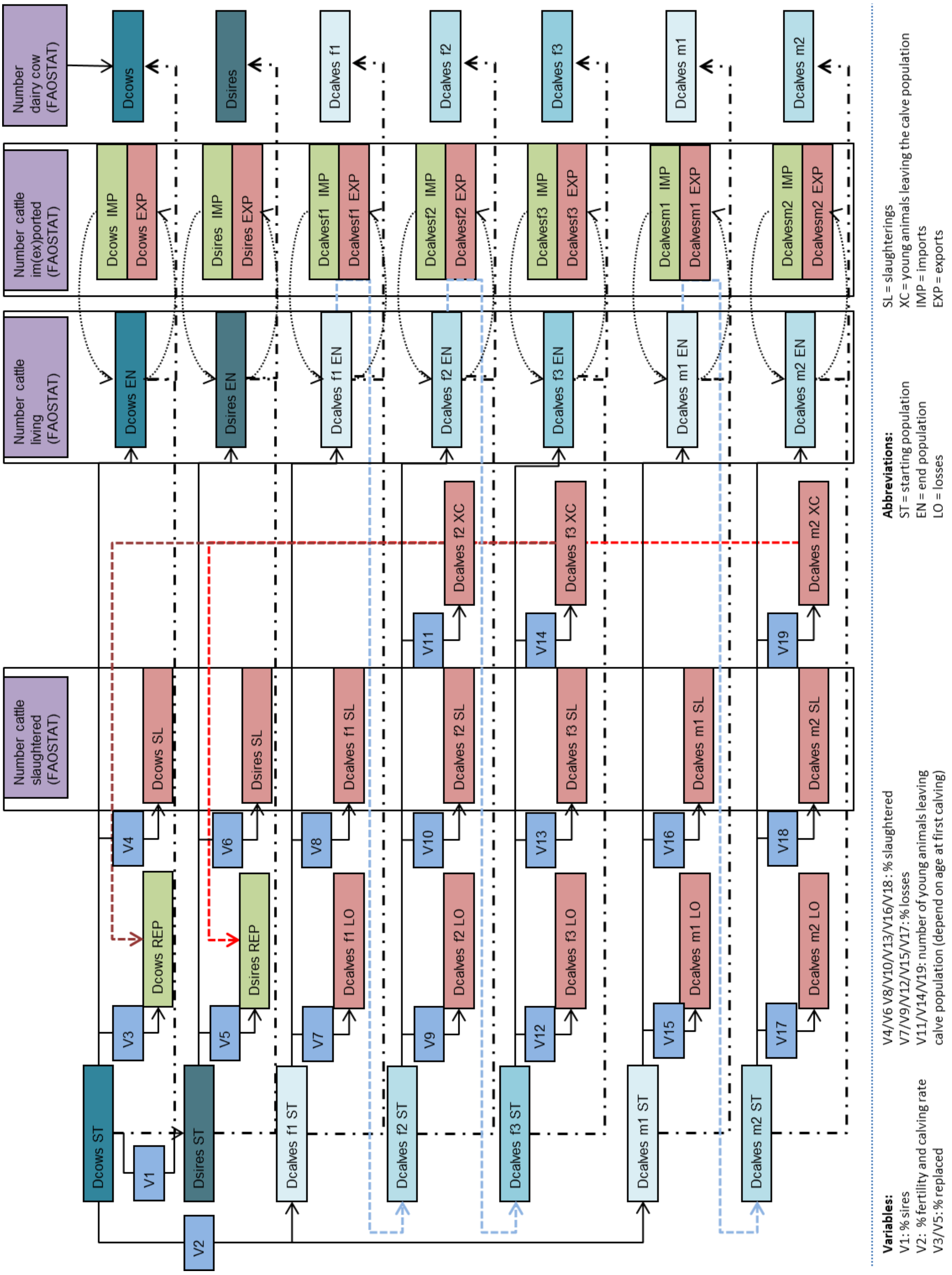

Figure S1 Illustration of dairy cow herd structure

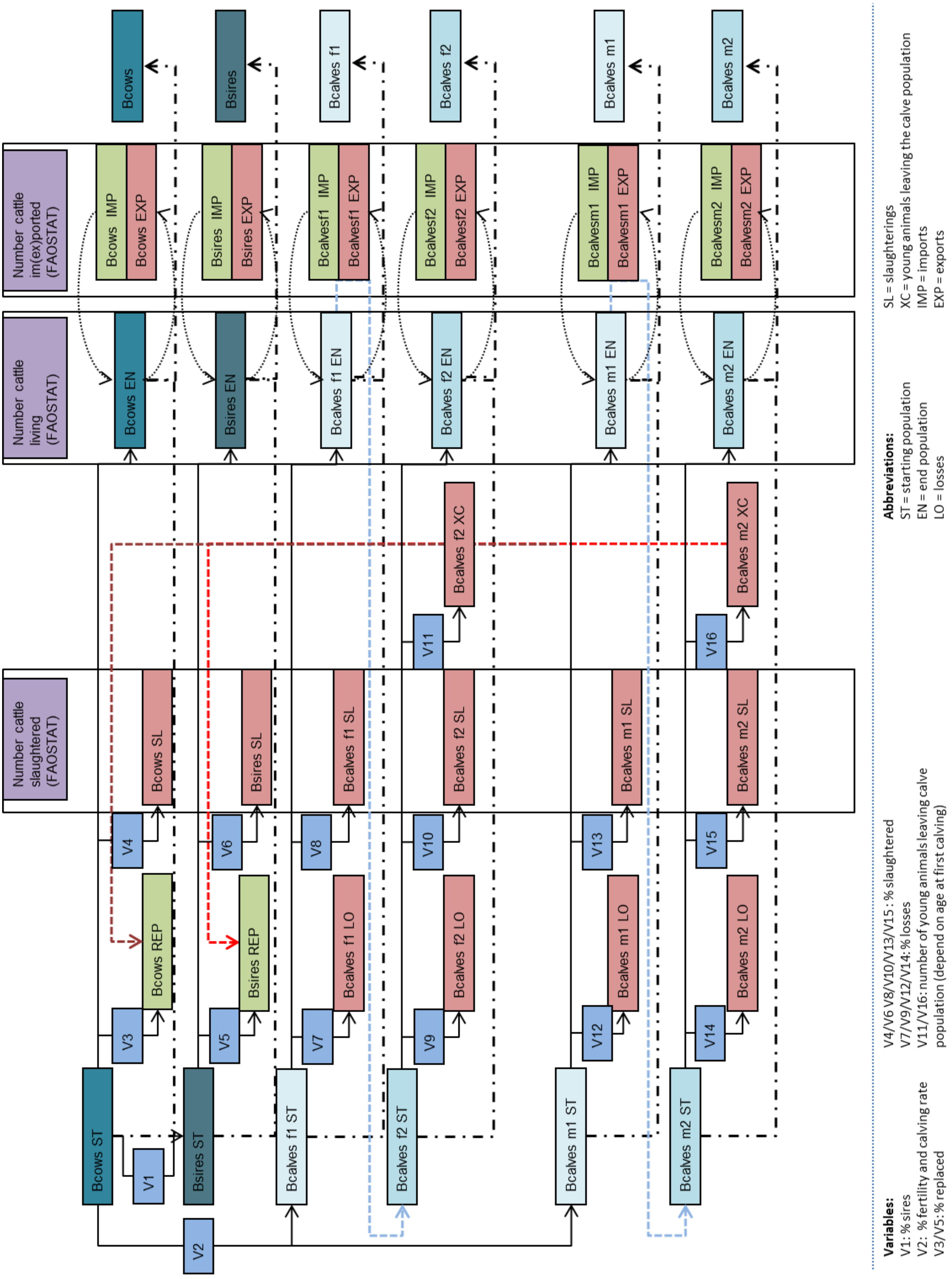

Figure S2 Illustration of beef cow herd structure

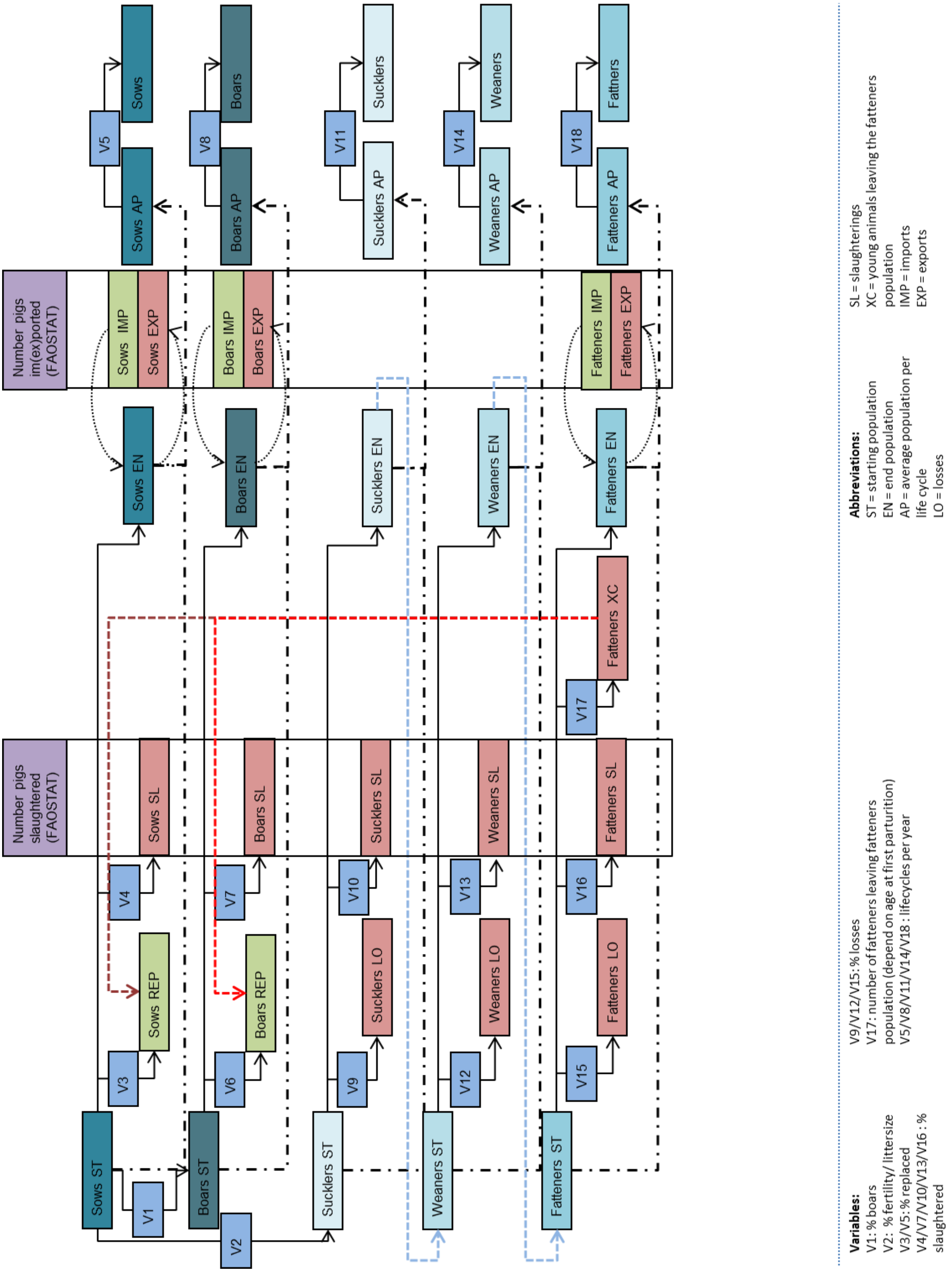

Figure S3 Illustration of pig herd structure

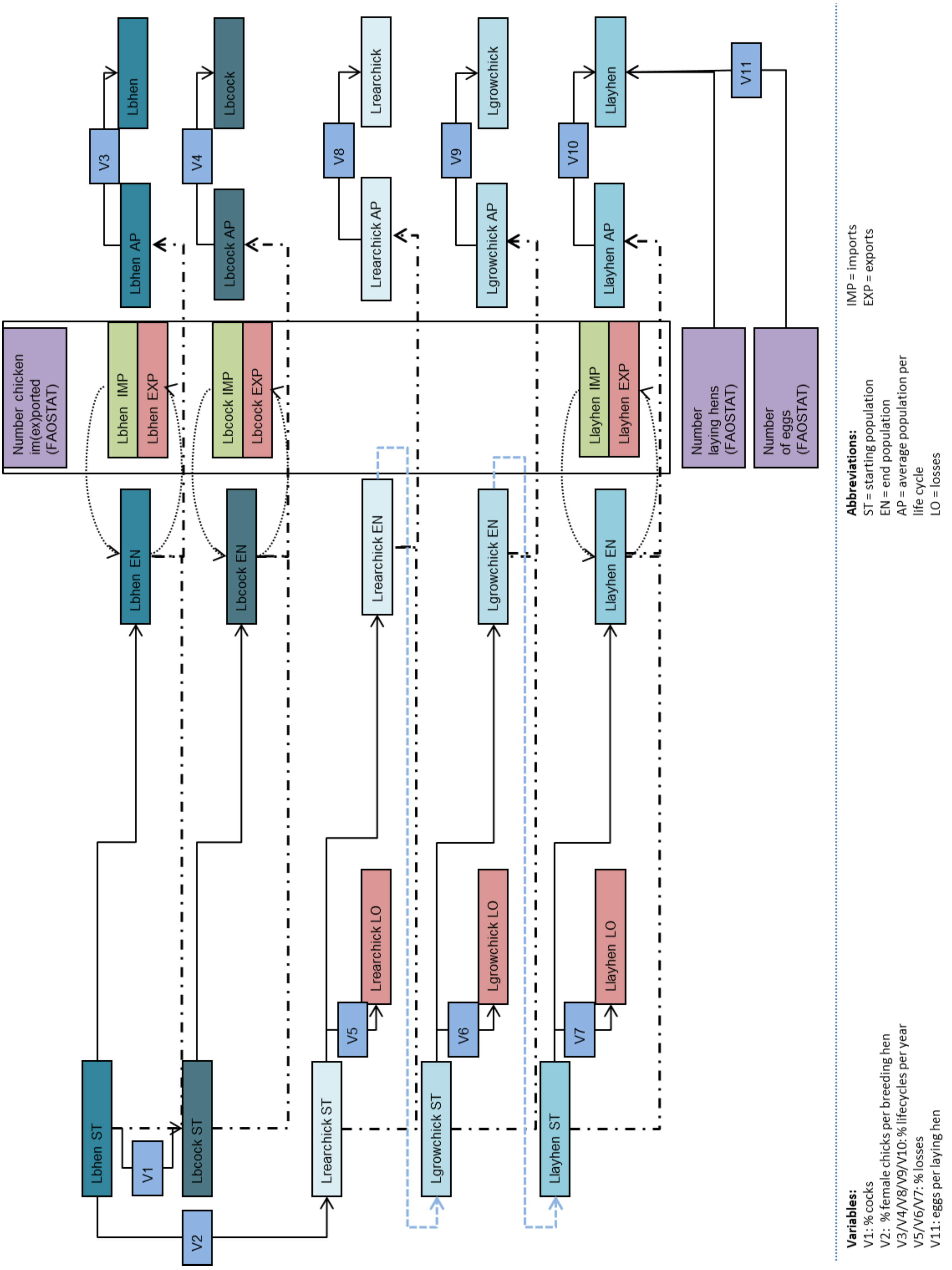

Figure S4 Illustration of laying hen flock structure

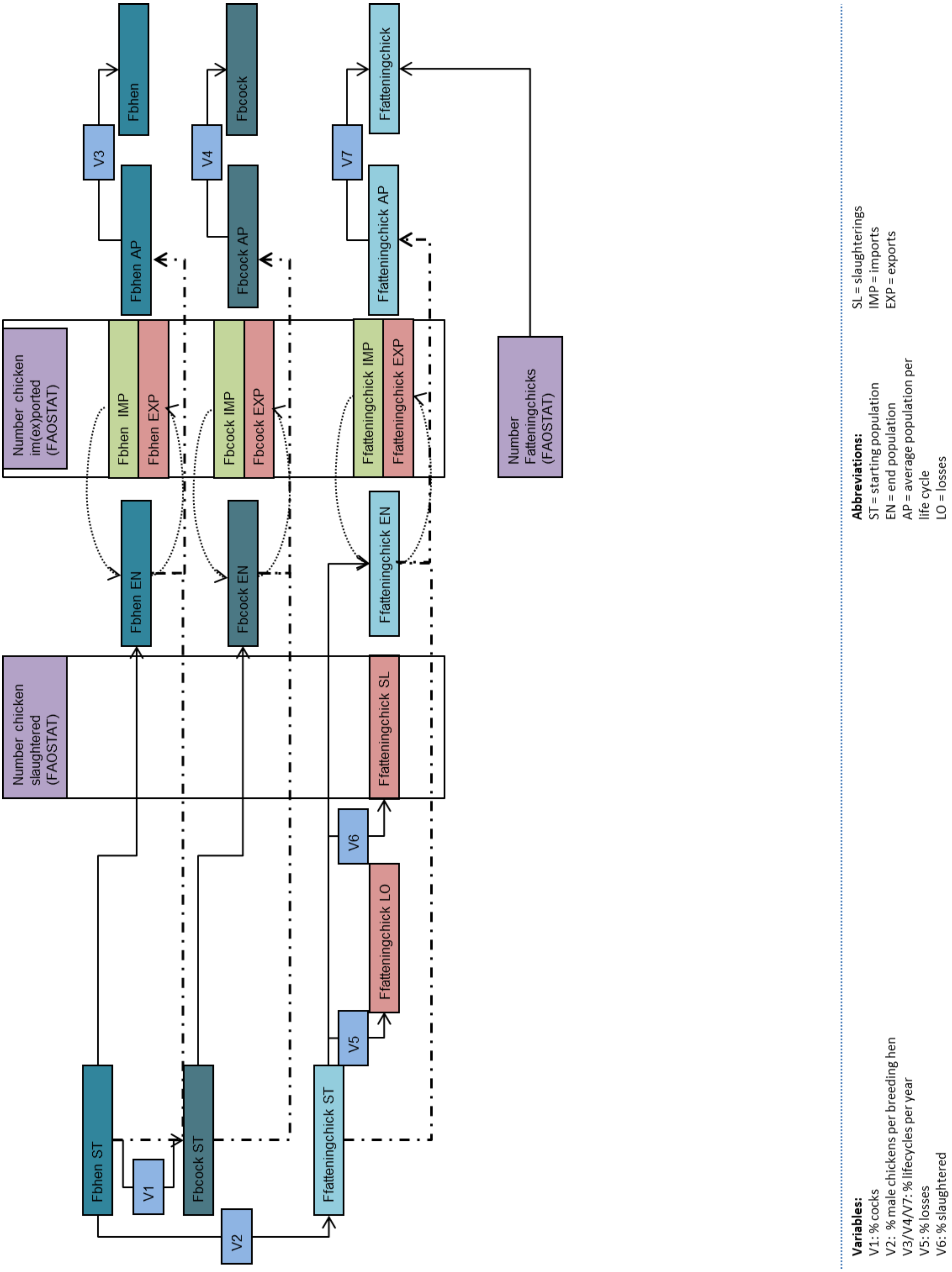

Figure S5 **Illustration of fattening chicken flock structure**

1.3.3 Inputs for plant production activities

The inputs for crop production activities that are included in SOL-m are land, water, nutrients, pesticides, seeds, electricity and fuel use, and buildings and infrastructure. Management practices that are necessary to obtain outputs from an activity, such as tillage, irrigation and harvesting, are also covered. These are described in more detail in the sections below.

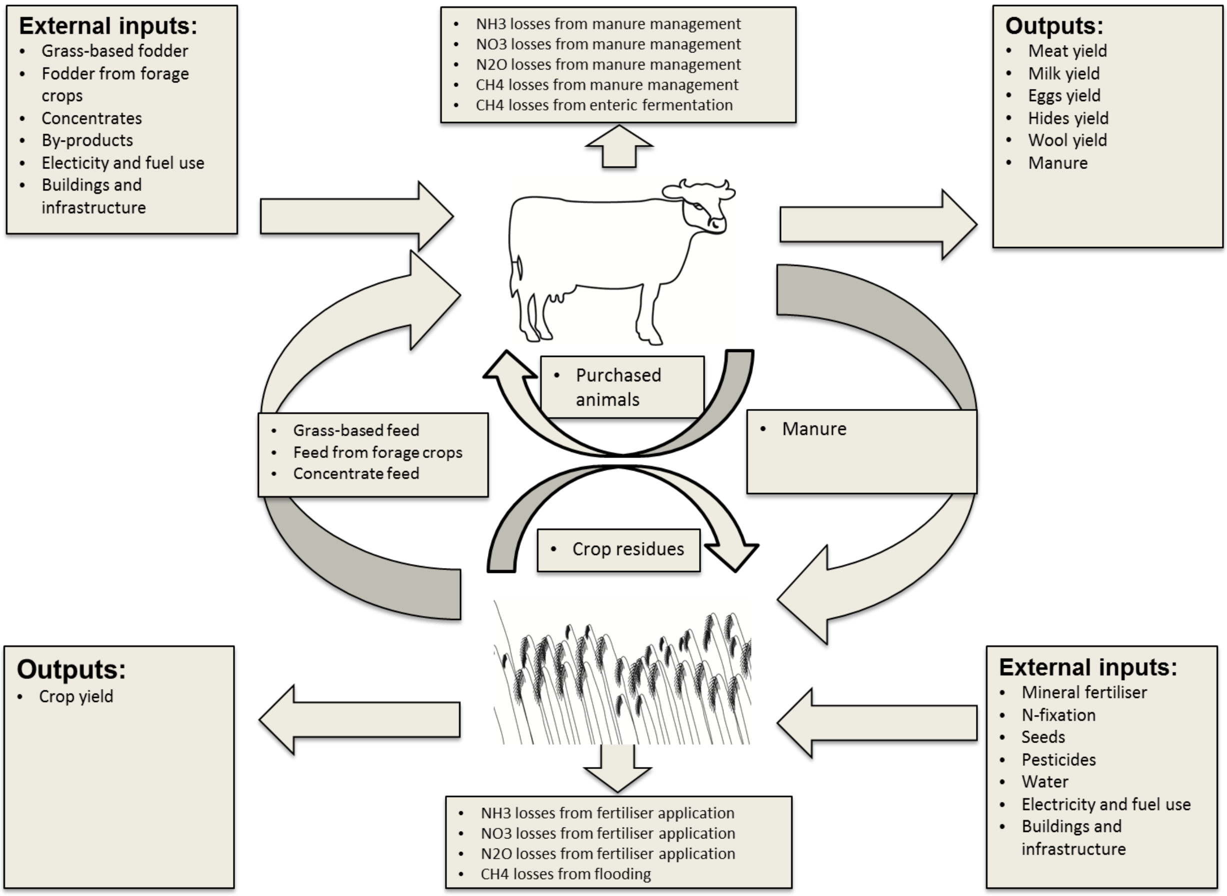

Figure S6 Overview of internal flows, inputs, outputs and losses of agricultural systems in SOL-m

1.3.3.1 Mineral fertilizer inputs

Country-specific mineral fertilizer inputs for each activity were defined using FERTISTAT data, which specifies total mineral N, P and K input per country. Country-level fertilizer amounts are allocated to the crops in proportion to their total nutrient demand. The nutrient demand per crop is calculated from production, seed and residue quantities and their respective nutrient contents. This allocation of mineral fertilizer supply to primary crop activities is only for N. The P and K demand used to calculate the quantities of mineral P and K fertilizer is the residual after accounting for the P and K quantities from manure and crop residues that are applied (see 1.3.3.2 and 1.3.3.3).

1.3.3.2 Manure inputs

N demand drives the supply of the different fertilizer types to crops. Some amount of P and K is also applied for organic fertilizers, manure and crop residues. These nutrients are taken into account when calculating mineral P and K fertilizer quantities, as the crop P and K demand used for this (see above) is reduced by the P and K quantities already applied through organic fertilizers.

It is assumed that 50% of all manure excreted by ruminants remains on grassland. The rest of the manure is allocated to crops and grassland as follows. Manure is applied to grasslands until the nutrient demand is met (in addition to the 50% of manure that remains on grasslands anyway). If some

manure remains unused after this, it is assumed that this is applied to croplands, together with all mineral fertilizer, crop residues and N-fixation available, in proportion to demand.

1.3.3.3 Crop residue inputs

In all systems, the total amount of crop residues is applied to the crop areas in proportion to demand. It is assumed that the residues nutrient uptake efficiency is the same for all systems. The amount and nutrient contents of crop residues were estimated based on several data sources [16-19]. Where no data were available, values from similar crops have been used, scaled with yields.

1.3.3.4 Nitrogen fixation

The fixation rates reported in[20] were used to determine N input due to atmospheric fixation through legume crops and due to legumes on grasslands . N-fixation also takes place in rice and sugar cane cultures. These values were also taken from[20].

1.3.3.5 Seeds

The amount of seeds needed for each crop was taken from several publications and databases [17, 21-26]. For crops with very low seed weights (e.g. vegetables), the weight was set to zero. The nutrient contents of seeds were assumed to be equivalent to the nutrient contents of the produce (as is the case for crops with relevant seed weights). Where no data were available, values from similar crops have been used.

1.3.3.6 Irrigation water

The amount of irrigation water per crop and country was taken from [27]. AQUASTAT is part of the FAOSTAT database and covers a range of variables related to agricultural water use.

1.3.3.7 Pesticides

There is no comprehensive database on crop and country-specific pesticide use. FAOSTAT does not specify pesticide use at country level for many countries. As pesticide production is mostly relevant for energy and GHG emissions, we used life cycle inventories in Nemecek, Dubois [28], Nemecek and Kägi [29] and Schader [30] and extrapolated the results to other countries and crops according to the procedure described in the main paper.

1.3.3.8 Electricity, fuel use and buildings

We used life cycle inventories by Frischknecht, Althaus [31], Nemecek and Kägi [29] and Schader [30] to calculate the inputs for electricity and fuel as well as for buildings. The life cycle inventories differentiate between the following processes: tillage, seeding, fertilization, spraying, irrigation, flooding (paddy rice), harvesting, on-farm transport and drying harvest. The results were extrapolated to other countries and crops where data were unavailable according to the procedure described in the main paper.

1.3.4 Outputs of plant production activities

The outputs of plant production activities in SOL-m are production quantities, crop residues and losses from fertilizer application and rice flooding.

1.3.4.1 Crop and grass yields

Production quantities in SOL-m are calculated from areas harvested and yields. Data for base year yields were taken from FAOSTAT, using the average (weighted by production) of the five years 2005-2009. For grasslands, yields were based on alternative data sources, as data were unavailable in FAOSTAT [29, 32]. This data includes conventional and organic production.

Food and feed production and nutritional value were determined using the production quantities from the FAO Food Balance Sheets and the commodity trees (see below, Sections 1.3.7.1 and 1.3.7.2). In particular, food production results in a considerable amount of by-products that are mainly used as animal feed. The corresponding physical and nutrient quantities are also from the commodity trees (1.3.7.2).

1.3.4.2 Crop residue outputs
Crop residue quantities and nutrients are calculated from crop quantities, crop residue shares (in relation to yields) and crop residue nutrient contents (see Section 1.3.3.3).

1.3.4.3 Emissions
For crop activities, the calculation of NO_3, NH_3, N_2O and CH_4 emissions is based on the IPCC guidelines [14].

1.3.5 Inputs for livestock production activities
Inputs to animal production are animal feed and energy requirements for buildings, in-barn processes and fences. Feed is described in Section 1.3.5.1.

1.3.5.1 Feeding rations
In SOL-m there are four animal feed types: grass-based fodder, concentrates (all food crops utilized as animal feed, all food-by-products used as animal feed), crop residues and forage crops. The herd structures account for imported animals that could also be seen as inputs to animal production (Section 1.3.2).

The model does not address animal feed additives, medicines or the water demand of animals. Feeding rations describe the shares of the four different feed types in the animal diet. These shares were taken from Herrero, Havlik [33]. The feed type "stover" was assigned to crop residues. The feed type "occasional" in Herrero, Havlik [33] and in the associated references was partly attributed to forage crops (for cattle) and partly to grasslands (for sheep and goats, 90% grass, 10% forage). This category was important for sheep and goats but not for cattle. For sheep and goats it was assumed that only a minor part of feed not from "concentrates" was related to crops that would use arable land for their production and that thus the majority of this non-concentrate feed was sourced without competition with food production (i.e. from grassland, road-side grasses, etc.).

Data on the specific feed energy demand of different livestock types were taken from the literature cited in Section 1.2. For cattle, pigs and chickens, an average feed demand per-animal was determined across the different livestock sub-classes (as described in Section 1.3.2 on herd structures).

1.3.5.2 Farm infrastructure and energy use
Farm infrastructure is relevant for modelling non-renewable energy use and GHG emissions. Our inventories are based on data in Nemecek, Dubois [28], Nemecek and Kägi [29] and Schader [30]. The inventories were extrapolated to different activities and countries as described in the main paper.

1.3.6 Outputs of livestock production
In SOL-m the outputs from animal production are yields, manure, and CH_4 emissions due to enteric fermentation.

1.3.6.1 Livestock Yields
Livestock yields are distinguished between meat, milk, eggs, honey, beeswax, wool and hides based on FAOSTAT. Base year yields represent weighted means of the years 2005-2009 for each country.

1.3.6.2 Manure output
Manure quantities and nutrient contents associated with different livestock activities and sub-activities were taken from various sources [14, 34-44].

1.3.6.3 Emissions
Livestock emissions from manure management (N_2O, NH_3, NO_3, CH_4) and CH_4 emissions from enteric fermentation are calculated in SOL-m according to the Tier 1 approach from the IPCC Guidelines[14]. For a more detailed modelling of cattle, pigs and chickens, country-specific herd structures are used (Section 1.3.2).

1.3.7 Modelling utilization of agricultural products

1.3.7.1 Food Balance Sheets

The FAOSTAT Food Balance Sheets [45] are a data set that contains information on production, imports, exports, stocks and stock changes and domestically available quantities (i.e. production + import – export – stock-changes) of each of the primary activities covered in the model. Food Balance Sheets also contain information on "utilization shares", which describe how much of the domestically available quantity of each commodity is used for food, feed, seed, waste and other utilizations.

The FBS have recently been reorganised and some underlying calculations have changed. In this work, we still use the FBS as they have been compiled before this reorganisation (before 2013).

In SOL-m, we use the detailed data from the FAO-internal "working system" that underlies the published food balance sheets. This data covers not only primary products but also intermediate and by-products of the various commodity processing chains (cf. next section on commodity trees). This data contains information on the utilization of each of those intermediate and by-products as well, i.e., for example, on how much feed is derived from brans. This detailed information allows to derive how much main and by-products correspond to one unit of primary product equivalent and how much food and feed energy and protein are derived from those main and by-products.

In general, biofuel utilization is not covered in the FBS data, but in some cases it is (this depends on how the various countries report the data to FAO). We checked this for important biofuel countries such as USA and Brazil, where the utilization category "other" is, for example, huge for maize and sugar cane primary crop equivalents, respectively, and covers biofuel use. In some cases, the detailed working system data then reports feed utilization for the by-products from these quantities as well. Where it does not, we proceeded as described in sections 1.4.1.1 and 1.4.1.2. For consistency, we used other more encompassing data on biofuels to derive the biofuel utilization shares, though (cf. section 1.3.7.4 below). Animal feed for aquaculture is neither covered consistently for all countries in the FBS data and we thus used other sources to derive the share of concentrate feed for aquaculture (cf. references in 1.3.7.3 and section 1.4.1.7).

1.3.7.2 Commodity trees

The commodity trees [46] are part of the FAOSTAT data and provide information on how commodities relate to each other and to primary activities, using country-specific averages from the years 1992-1996. SOL-m includes 690 underlying commodities that are associated with primary activities (e.g. for the primary activity 'wheat', the underlying commodities are wheat flour, bran, germ, beer, starch, etc.; for the primary activity 'cattle', there are two yield types, milk and meat, and the underlying commodities are milk, whey, cheese, meat, offal, animal fats, etc.). Certain relations exist among commodities: some commodities are by-products of another main product (e.g. wheat brans and germ are by-products of wheat flour); various commodity trees can produce the same commodity (e.g. alcohol can be derived from a range of grains and sugar crops); and certain commodities are derived from other commodities. To account for these relationships and processes, information on extraction rates is provided.

This information is used to translate the Food Balance Sheet data on quantities of commodities into primary product equivalents (e.g. 750 kg of wheat flour is equivalent to a tonne of wheat grains). Primary crop equivalents are needed to calculate the nutritional values of primary product quantities (it is not the tonne of wheat that is eaten by humans, but the quantity of wheat flour, bread, germ oil, bran oil, starch, etc.). The nutritional value of commodities for humans (calories, protein, fat) and for animals (metabolizable energy, gross energy, crude protein) are combined with the respective quantities and the utilization shares from the Food Balance Sheets to calculate the nutrient supply from a certain quantity of primary products.

We used commodity trees to calculate nutrient supply for all crop and animal activities besides fish and seafood. For fish and seafood, we used aggregate FAOSTAT data at the primary product level. Detailed commodity trees allowed us to calculate the feed quantities from by-products of food production (e.g. brans, whey, etc.; see below).

1.3.7.3 Fish, Seafood and aquaculture

Fish, seafood and aquaculture supply in the base year were taken from FAOSTAT and FAO publications [47-49]

1.3.7.4 Biofuels

Biofuel shares in food crops for the base year were taken from OECD/FAO [50]. To be consistent, we used this data also in case biofuel data has been reported in the food balance sheets (cf. section 1.3.7.1 above).

1.3.7.5 Trade, markets and prices

Trade was not modelled explicitly. It is reflected in the domestically available quantities that are the basis of the food supply (see Section 1.3.7.1) and that account for imports and exports. To calculate domestically available quantities in the scenarios, we used the quantities forecasted in Alexandratos and Bruinsma [12]. This assumes that trade patterns in all scenarios are the same as in the reference scenario. The disadvantage of not modelling trade patterns explicitly is that supply and demand cannot react to changes in prices, based on price elasticities (i.e. how demand and supply change with price changes). This problem is less relevant for our model, as the aim is to provide a physical mass balance and a physical/technical/agronomic assessment of the viability of the scenarios investigated, irrespective of economic effects.

1.3.8 Modelling food demand

For the modelling scenarios, population forecasts from UN [51] were used, adjusted according to the numbers used in Alexandratos and Bruinsma [12]. Food demand was based on the per-capita Average Daily Energy Requirements (ADER) in FAO [52]. ADER data (in kcal) is differentiated by country. Average daily protein requirements (in grams) were calculated from ADER using a factor of 0.10/4 (10% of the energy supply is from proteins and 1g protein carries 4 kcal). Therefore, the protein/calorie ratio is 0.1, which can be seen as a lower acceptable limit according to FNB [53]. These data account for population structure and body weight in different countries. For the scenarios, we assumed that ADER and protein requirements (and therefore, average weight) will not change in the future.

1.3.9 Additional information on environmental impacts

The main body of the paper contains some methodological details for the indicators land occupation, N-surplus, GHG emissions and deforestation. In the following, similar modelling details are provided for the indicators P-surplus, renewable energy use, pesticide use, freshwater use and soil erosion. Further details on how the various variables used in the calculation of these indicators are determined can be found above, where fertilizer inputs, pesticide classifications, etc. are described.

1.3.9.1 P-surplus

P is immobile in the soil but can be lost in incidences of erosion [39]. Like N, P can cause damage to aquatic and terrestrial ecosystems. P-surplus is defined as the difference between the P-outputs (e.g. yields) and P-inputs (e.g. fertilizer quantities). For P-surplus, the same procedures apply as for N. Relevant inputs (IN) and outputs (OUT) for calculating the P-surplus are mineral P fertilizer, P_2O_5 in manure, crop residues and yields. IF is defined as the P-content of the inputs, while all outputs are defined as negative values.

1.3.9.2 Non-renewable energy use

The agricultural sector is dependent on fossil fuels. This indicator measures how food production contributes to the depletion of this natural resource. Most of the crop production-related categories are linked to farm activities. This includes energy use for seeds, crop protection, fertilization, mechanization, organic fertilization, fences and depots for roughage. Animal husbandry data (including buildings) were taken directly from the ecoinvent 2.0 database and [43, 44].

While energy use from fertilizer production was modelled specifically for the fertilizer amounts used for each crop in each country, the input of energy carriers was modelled according to the ecoinvent 2.0

and SALCA inventories [45]. Energy use for transportation was not considered due to a lack of quality data on trade. For activities where no data were available, we extrapolated inventories from the most similar activities where data were available. Yields were specifically defined for activities and countries according to FAOSTAT. Due to lower degrees of mechanization and lower capital intensity in developing countries, we adapted the data by setting the following components to zero: barns, installations, depots for fodder, harvesting and drying.

For the modelling scenarios, it is assumed that the share of non-renewable energy for fuels and electricity stays constant and that there is no technical progress in the energy efficiency of the machinery used. This is a large assumption, but it is legitimate for the results we derive with this model, which are relative changes between a reference scenario and the scenarios tested.

1.3.9.3 Water use

The agricultural sector is the largest sector in terms of water withdrawals, using about 70% of freshwater resources worldwide [54]. We tested the water footprint [55] and AQUASTAT [27] data for modelling water use in the SOL-model and decided to use AQUASTAT data due to better coverage and compatibility with other data used and better data quality as judged by experts from FAO. We only used irrigation water, as green water would lead to a large overestimation of total global water use for agriculture. Furthermore, the impact of agriculture on water pollution (grey water) is already included in the model by calculating N and P surplus and pesticide use intensity. Final calculations were made using

1.3.9.4 Pesticide use

As there is no consistent dataset on pesticide use covering different countries, we developed an impact assessment model for assessing pesticide use incorporating three factors: pesticide use intensity per crop and farming system (PUIj,k), pesticide legislation in a country (PLi), and access to pesticides by farmers in a country (APi) (cf. table S3 below). Each factor was rated on a scale from 0 to 3 by FAO-internal and external experts (Jan Breithaupt, FAO, involving experts from regional FAO offices; Frank Hayer, Swiss Federal Office of the Environment; Bernhard Speiser, Research Institute of Organic Agriculture, FiBL) with experience in different countries and with different methods of calculating pesticide impacts (life cycle assessment, risk assessment). The descriptors for each scale have been designed so that risks from pesticides are 0 if only one of the three model parameters is equal to 0. For instance, if there are no harmful pesticides used in a crop, or if in a country legislation completely bans harmful pesticides or farms do not have access to pesticides at all, the impact factor for a crop-country combination will be 0.

As an example, the pesticide use IF for coffee production in Ghana was 6 as: PUI was rated as 3, PL was rated as 2 and AP was rated as 1. Values for PL and AP are shown in the Table S41 in the appendix and values for PUI can be found in the Table S42. To calculate crop and country-specific pesticide use impact factors (IF), the three factors were multiplied together (Equation 4).

$$IF_{i,j,k,l,s,pesticide\ use} = PUI_j * PL_i * AP_i \ \forall\ i,j,k,l,s \qquad (4)$$

Finally, the crop-specific factors for all agricultural activities in a country were summed and aggregated at global level according to Equation 2, with pesticides qualified as IN and the crop-and country-specific toxicity rating as IF.

Table S3 Pesticide model classifications

Rating	Pesticide level per crop (PUI)
0	No harmful pesticides* used
1	Low level of pesticide application
2	Medium level of pesticide application
3	High level of pesticide application / harmful pesticides used*

* WHO classification

Rating	Pesticide legislation per country (PL)
0	All chem.-synthetic pesticides (WHO-classes 1-2) banned
1	Rigid pesticide legislation and control excludes harmful pesticides*
2	Average pesticide legislation and control
3	Legislation does not preclude the use of harmful pesticides*

*WHO classification

Rating	Access to pesticides per country (AP)
0	Farmers have no access to chem.-synthetic (WHO-classes 1-2) pesticides
1	Only few farmers have access to chem.-synthetic pesticides (max. 10% of the cultivated land is treated with pesticides)
2	Some farmers have access to chem.-synthetic pesticides (10-50% of the land that deserves treatment is treated)
3	Many farmers have access to chem.-synthetic pesticides (min. 50% of the land that deserves treatment is treated)

1.3.9.5 Soil erosion potential

Fertile soil is one of the most scarce production resources. Soil erosion potential was modelled as a function of the erosion susceptibility of different crop types. To calculate erosion susceptibility we classified each crop according to the period of time during the cropping period where the soil is left bare. On a scale from 0 to 2, permanent grasslands were classified as 0, crops with a short period of soil left bare were classified as 1 and crops with a longer period of bare soil like maize or beets were classified as 2. The erosion susceptibility of crops was used as an impact factor (IF). Classifications were based on the agronomy literature and expert consultations [60-66]. Results based on expert consultations were cross-checked against the literature, for example [67].

The susceptibility to soil erosion of various crops was combined with data from an extensive literature review of national average water–induced soil erosion rates (excluding wind erosion, as data were largely unavailable). Specific values were found for 58 countries for croplands and 15 countries for grasslands (SI, Table S43). Regional average values were assigned to countries where data were missing. The soil erosion levels were measured in tonnes of soil lost per ha per year. Crop-specific impacts for soil erosion were calculated by multiplying the national per ha soil erosion values by total national cropland and grassland areas (differentiated values were used for cropland and grassland). This product was then divided by the erosion susceptibility-weighted crop and grassland areas to arrive at an average per ha soil erosion value. Finally, the average value was multiplied by the erosion susceptibility of a crop, to give crop-specific per ha soil erosion values.

1.4 Modelling the food sector in the scenarios

In the following sections we describe how the scenarios for 2050 were modelled. The assumptions and references used are described where they are different from the base year.

1.4.1 Reference scenario

The following sections describe specific assumptions relating to the reference scenario. Where appropriate, some assumptions that apply to other scenarios are also introduced. Further technical assumptions for the scenarios are discussed separately in Section 1.4.2.

Forecasts for domestically available quantities and production for crop and livestock activities are used in SOL-m and *per-capita* energy intake was calculated according to Alexandratos and Bruinsma [12].

Table S4 Overview of model assumptions for the scenario types

Parameter	Base year	Reference scenario	Reducing food-competing feedstuffs (FCF) (different levels down to 0% FCF)
**Year**	2005-2009	2050	2050
**Human population**	According to FAOSTAT	According to FAOSTAT	According to FAOSTAT
**Calorie and protein supply per person**	According to FAOSTAT	According to "World agriculture towards 2030/2050: the 2012 revision" [12]	Calorie supply equal to the reference scenario; for comparison, scenarios with constant protein intake are also assessed.
**Share of livestock products in human diets**	According to FAOSTAT	According to "World agriculture towards 2030/2050: the 2012 revision" [12]	Fraction of livestock-based food energy in total food energy supplied by the food system.
**Increase in crop yield**	n.a.	According to "World agriculture towards 2030/2050: the 2012 revision" [12]	As in the reference scenario. A sensitivity analysis based on projections of climate change impacts on yields is included.
**Increase in cropping intensity**	n.a.	According to "World agriculture towards 2030/2050: the 2012 revision" [12]	As in the reference scenario.
**Ratio arable land / grassland**	According to FAOSTAT	According to "World agriculture towards 2030/2050: the 2012 revision" [12] (Net grassland stays constant, arable land increases)	Net grassland is kept constant as in the reference scenario; arable land change according to the amount of calories/protein supply.
**Deforestation**	According to FAOSTAT	Increased land areas increase deforestation, using the land areas forecasted and deforestation rates from FAOSTAT	If more/less land is needed to satisfy food availability, pressure on forests increases/decreases.

Parameter	Base year	Reference scenario	Reducing food-competing feedstuffs (FCF) (different levels down to 0% FCF)
**Ruminant numbers**	According to FAOSTAT	According to “World agriculture towards 2030/2050: the 2012 revision” [12]	Model-endogenous calculation of the number of animals that can be fed on available feed.
**Non-ruminant numbers**	According to FAOSTAT	According to “World agriculture towards 2030/2050: the 2012 revision” [12]	Model-endogenous calculation of the number of animals that can be fed on available feed.
**Share of feed types in feeding rations**	According to Herrero et al. [33]	As in the base year	Based on rations for base year and reference scenario but adapted according to supply of food-competing feedstuffs dropping gradually to 0%.
**Livestock yields**	According to FAOSTAT	According to “World agriculture towards 2030/2050: the 2012 revision” [12]	Based on reference scenario but yields decrease by up to 20% with zero food-competing feedstuffs, due to suboptimal feed composition. As a sensitivity analysis, scenarios are also calculated for 0% and up to 40% yield reductions.
**Utilization shares**	According to FAOSTAT	According to “World agriculture towards 2030/2050: the 2012 revision” [12]	Feed share of primary food crops reduced (0% for 100% reduction of food-competing feedstuffs).

1.4.1.1 Activity levels

For the reference scenario, activity levels are calculated by Alexandratos and Bruinsma [12], including the data on yield increase, cropping intensity forecasts and dietary changes. Dietary changes are reflected in changes in available quantities of various commodity groups (e.g. cereals, pulses or vegetables). As we use a mass balance model with the same trade patterns in the scenarios as in the reference scenario Alexandratos and Bruinsma [12], we assume that these dietary changes are reflected in the areas harvested, quantities produced and in the domestically available quantities.

Areas harvested are the basis for the calculation of production quantities and environmental effects according to input and output coefficients. Food quantities, calorie and protein supply are calculated from the domestically available quantities (for details, see Section 1.3.7.1).

The number of non-ruminants in the scenarios is determined from the available animal feed energy from concentrates according to feeding rations and per animal head feed energy demand. The number of ruminants is determined from the available feed energy from grasslands, forage crops and concentrates, according to feeding rations and per animal head feed energy demand. For non-ruminants, feeding rations are always 100% concentrates. For ruminants, feeding rations determine how much of the demand has to be met from grassland and how much from concentrates and forage. The share of demand for the different feed types is used to calculate the demand per animal feed type per head of animal and the number of animals from total supply from each animal feed type. The lowest number of animals from these values for each feed type is then taken as the number of animals that can be fed, given the feed supply and the feeding rations. For ruminants, this is usually also the number derived from grassland supply. The shares of different ruminant and non-ruminant animal types are determined so that the share in total ruminant and non-ruminant feed demand for these animal types stays the same as in the reference scenario.

1.4.1.2 Feeding rations

Feeding rations in the reference scenario were assumed to be the same as in the current situation. In the current situation (based on FAOSTAT data), we observed some discrepancies between feed energy supply, given the aggregate feed availability, and feed energy demand based on animal numbers. The difference is further accentuated in the reference scenario [12]. To allow comparability of results between the reference scenario and the other scenarios, we used the supply/demand ratio as calculated for the reference scenario to determine the animal numbers in the other scenarios, assuming the same difference between supply and demand in the scenarios as in [12].

1.4.1.3 Yields

Annual yield increases are taken from Alexandratos and Bruinsma [12]. Yield increases of grass are assumed to be 10% of the average of other crop yield increases, due to smaller research investments. Cropping intensities (i.e. how many times harvests are possible on a certain area, or the fraction of overall cropland that is fallow/idle) for the current situation and forecasts on their values in the scenarios for 2050 are taken from Alexandratos and Bruinsma [12]. Nutrient quantities are calculated from the production and residue quantities and their respective nutrient contents, following the same method as in the base year.

1.4.1.4 Inputs

Mineral fertilizer inputs and irrigated land areas for the reference scenario were taken from the detailed data underlying the calculations in [12] that were made accessible by FAO. Manure and crop residues inputs and N-fixation were derived from the number of animals and production quantities, and allocated to the field according to the same procedure as in the base year described above. Seeds, pesticides, energy use were based on the same factors per quantity or hectare as in the baseline.

1.4.1.5 Food Balance Sheets

In the scenarios, the utilization shares in the base year were adapted according to concentrate feed reduction by reducing the feed share and correspondingly increasing the food share for food crops.

1.4.1.6 Commodity trees

We assume identical commodity trees in the scenarios as in the baseline (i.e. an identical composition of how primary production is consumed). The same baseline ratio was used to determine the nutritional values (e.g. food calories, protein and fat, feed metabolizable energy) that correspond to physical primary product quantities produced or domestically available in the scenarios (see 2.6.7.2).We differentiate between food calories, feed metabolizable energy, etc. from direct production and from by-products. By-products cannot be linked to primary product quantities. For example, 1 tonne of wheat grains corresponds to 750 kg wheat flour, 200 kg brans and 50 kg germs. Therefore, the primary crop equivalent of 750 kg wheat flour is 1 tonne of wheat grains, and similarly, the primary crop equivalent for 200 kg wheat brans or 50 kg wheat germs are each 1 tonne of wheat grains. But only 1 tonne (not 3 tonnes) is accounted for in the model. We resolved this allocation problem by identifying main products, to which the primary crop equivalents were assigned, and by-products, that do not correspond to a primary crop equivalent. This distinction is important for concentrate feed produced from human-edible crops. Compared to concentrates from primary production, concentrates produced from by-products of human food do not occupy any additional agricultural land or cause additional environmental impacts (as these are assigned to the main products in SOL-m). This allocation could be further refined in an LCA context but it provides a clear rule for the scenarios and allows transparent identification of land use and environmental impacts of food crop and animal production.

1.4.1.7 Fish, seafood and aquaculture supply

Consumption forecasts for fish and seafood are taken from OECD/FAO [50], linearly extrapolated to 2050. In scenarios with reduced concentrate use, the output of aquaculture (amount of fish and seafood) that is fed by concentrates is also reduced. The share of aquaculture that is fed (with concentrates) is taken from FAO [49] and OECD/FAO [50], linearly extrapolated to 2050. Fed aquaculture makes up 17% of total fish and seafood supply in the current situation [48, 49]; this is derived from the share of 35% aquaculture in in total fish and seafood supply in 2007, and the fact that 50% of this are fed [47]. In the reference scenario, fed aquaculture makes up 45% of total fish and seafood supply [50]; based on the assumption that about 50% more aquaculture than capture will be present in 2050, i.e. 60% aquaculture, 40% capture, assuming a continued increase as forecasted till 2021; it is further assumed that a 50% higher share than today will be fed, i.e. in total, 75% of aquaculture are fed; thus, as aquaculture comprises 60% of total fish and seafood supply, 75% of 60% are fed aquaculture, i.e. 45%). The reductions of fish and seafood supply are calculated from the share of concentrates used by aquaculture and the change in concentrate quantities in the scenarios, assuming that the same share of concentrates is used for aquaculture as in the reference scenario. The share of total concentrates used for aquaculture is 8%, composed from roughly 4% concentrate compounds and 4% from informal sources, e.g. on farm feedstuff, fish trash, etc. [47]. As a rough estimation, we have adjusted global averages downwards for land locked countries (0.1%), and for Europe and Africa (1%), based on the prevalence of aquaculture in relation to other livestock production.

1.4.1.8 Biofuels

The most recent biofuel forecasts that were available when SOL-m was developed only extended to 2012 [50]. Therefore, for all crops produced, we have assumed the same utilization shares for biofuel use in 2050 as in 2012. This is the same approach used by [12], where they model 2050 with the same biofuel quantity as 2019 (the final year of biofuel forecasts when their study was undertaken). However, we have assumed the same shares of biofuels in food crops, rather than the same quantities. Most forecasts assume increasing biofuel use. Therefore, the assumption of constant biofuel quantities corresponds to a “very low bioenergy” scenario.

1.4.1.9 Environmental impact factors

The same environmental impact factors from the base year (2005-2009) are used for the 2050 scenarios. This allows for a more reliable comparison between the base year and the scenarios. Therefore, potential technical progress in resource efficiency was not considered. For example, there may be higher shares of renewable energy in electricity mixes in 2050, or existing improved

application procedures for organic and mineral fertilizers may become more common and new ones may be invented.

1.4.2 Scenarios with reduction in food-competing feedstuffs

Further assumptions relating to the scenarios with a reduction of food-competing feedstuffs are discussed below. Scenarios with a 0 to 100% decrease (in 20% steps) of concentrates and forage crops fed to livestock were calculated. Reductions do not include by-products from food production which are not grown for the purpose of livestock feeding. Livestock numbers were calculated from per animal feed requirements and the available feed supply. Land no longer used for animal feed production (triticale, forage crops) was allocated to human food production according to the crop shares in the reference scenario. In addition, in the scenarios some cropland is released from reduced use of food crops for feed utilization (e.g. wheat or maize). The released cropland is allocated to the same food crops for food utilization. If less wheat is used as feed, the same amount of wheat is made available for food.

1.4.2.1 Feeding rations

Feeding rations of ruminants were linearly adjusted towards 100% grass-fed, in parallel with the reduction in concentrates to the "0% concentrates" scenario. In this scenario, no primary food products, feed crops (e.g. Triticale) or forage crops are fed to animals, but food by-products (e.g. brans, whey) are still fed as concentrates. In the reduced concentrate scenarios, all by-products from food production (brans, whey, etc.) that are not used elsewhere are still allocated to animal feed. It is assumed that processing patterns do not change, for example, that wheat flour is still predominantly consume d as white flour and whey is not widely used for human nutrition.

The feeding rations of cattle and other ruminants change in the scenarios with reduced concentrate availability. For monogastrics, feeding rations are always 100% from concentrates, but with further reductions of primary food products, feed crops and forage crops, the share of food by-products increases to 100%. This is reflected in reduced feed quality and reduced yields (see below).

The concentrates available in each scenario are allocated to the different animal types according to the nutrient demand shares of animal types in the reference scenario [12]. It is assumed that the rations between numbers of animals in livestock sub-classes are the same in the scenarios as in the base year.

1.4.2.2 Yields

Yields in the scenarios with reduced concentrate feed are assumed to be the same as in the reference scenario. However, it is likely that concentrate reductions will affect yields negatively. The possibility of reduced yields is reflected in the sensitivity analyses described below (1.4.3).

1.4.3 Sensitivity analysis

We conducted sensitivity analyses on the potential impacts of climate change on crop yields and on the potential impact of concentrate reduction on livestock yields.

Firstly, we calculated alternative scenarios with lower crop yield increases due to climate change impacts [54-59]. On average, yield increases are a third of the values in Alexandratos and Bruinsma [12], including the positive effects of CO_2-fertilization. Excluding CO_2-fertilization, which is highly uncertain and difficult to quantify, yield increases are zero or negative on aggregate. For the scenarios with climate change impacts on yield increases and without CO_2-fertilization, we assumed a zero average yield increase. This process provides a sensitivity analysis for yield increases, covering a range from zero increases to those used in Alexandratos and Bruinsma [12].

Secondly, monogastric animal yields are likely to decrease with a lower concentrate supply. This is because the concentrate mix becomes increasingly sub-optimal as the share of food by-products in the concentrate mix increases and producing a balanced concentrate mix becomes more difficult. Ruminant yields are also reduced with decreasing concentrate shares in the feeding rations. To account for this, we included a sensitivity analysis that assumes a 0%, 20% and 40% yield reduction for the scenario with 100% reduction in food-competing feedstuffs (i.e. for by-product based feed for monogastrics and grassland based feed for ruminants only). This yield reduction is assumed to scale

linearly with more concentrate feed (i.e. 0%, 10%, 20% yield reduction for 50% concentrates reduction). This range is based on literature on nutrient requirements and optimal feeding of various animal types [60-64]. We implemented this via such a sensitivity analysis, as from the literature, it has not been possible to derive such yield effects of general validity that would allow modelling this in more detail in the context of such a global model.

2 Results additional to those shown in the main paper

2.1 Results for the additional environmental impacts.

This subsection shortly presents a results for the environmental indicators not addressed in the main body of the paper. Similar to the N-surplus, P-surplus increases by 32% in the reference scenario, from 10.0 kg/ha/yr to 13.1 kg/ha/yr, as the demand for P-fertilization increases with the increase in volume of the whole agricultural production. Non-renewable energy demand and 18% and Furthermore, freshwater use increases by 59%, pesticide use by 9%, soil erosion potential by 9%. Also these effects are mainly driven by the increase in the total production volume. Energy demand relates to cropping areas and animal numbers and thus increases more than pesticide use and soil erosion potential, which are mainly driven by the area increases that are not that big in the reference scenario (and shifts in the share of crops in the total output). Freshwater use is mainly driven by the forecasts on how irrigated areas increase, which show a much higher growth than agricultural area in total.

For scenarios 0% food-competing feedstuffs, and compared to the base year, P-surplus drops by 14%, driven by the reduction in the overall production volume. Non-renewable energy demand decreases by 24%, showing the effect of the reduction in animal numbers that is larger than the overall drop in production volume. Freshwater use increases by 25%, which captures the increase in the share of irrigated areas, which however applies to less area only, as the total crop production is reduced. Pesticide use decreases by 15%, also mainly due to the drop in production volume and areas cropped. The soil erosion potential, finally, drops by 4%, driven by the reduction in production volumes and the specific shifts in crop shares in total production.

2.2 Additional results for scenarios with constant calorie provision in all scenarios for 2050

Table S5 **Daily intake of main food categories per person (fresh matter, primary crop equivalents, global average) in the base year, the reference scenario and in scenarios with reduced food-competing feedstuffs (climate change impacts on yields, 20% yield reduction in livestock due to reduction in food-competing feedstuffs) for constant energy provision in all scenarios**

Food types (PPE)[x]	Unit[#]	Base year (2005-2009)	Supply of food-competing feedstuffs to livestock in scenarios for 2050 [% of base year] 100%	80%	60%	40%	20%	0%	Difference of 0% food-competing feedstuffs scenario to base year	Difference of 0% to 100% food-competing feedstuffs scenario
**Plant products**	**g/(cap*day)**	**1442**	**1442**	**1444**	**1448**	**1445**	**1434**	**1413**	***-2%***	***-2%***
Grains	g/(cap*day)	519	511	544	568	585	592	590	*14%*	*16%*
Starchy roots	g/(cap*day)	185	186	193	198	202	203	201	*9%*	*8%*
Oil crops	g/(cap*day)	74	107	100	94	88	83	77	*4%*	*-28%*
Legumes	g/(cap*day)	42	53	61	71	84	99	119	*182%*	*126%*
Vegetables	g/(cap*day)	343	253	236	223	210	196	181	*-47%*	*-28%*
Fruits	g/(cap*day)	210	252	235	221	208	195	182	*-13%*	*-28%*
Sugars and sweeteners[+]	g/(cap*day)	65	76	72	68	65	63	59	*-8%*	*-22%*
Others*	g/(cap*day)	5	5	4	4	4	4	3	*-35%*	*-27%*
**Livestock products**	**g/(cap*day)**	**425**	**489**	**410**	**346**	**294**	**250**	**213**	***-50%***	***-56%***
Milk	g/(cap*day)	242	261	229	201	177	155	136	*-44%*	*-48%*
Meat	g/(cap*day)	110	136	101	75	55	39	27	*-76%*	*-80%*
Non-ruminants meat	g/(cap*day)	77	99	69	47	30	17	7	*-91%*	*-93%*
Ruminants meat	g/(cap*day)	34	37	32	29	25	22	19	*-42%*	*-47%*
Fish	g/(cap*day)	50	65	60	57	54	51	49	*-3%*	*-25%*
Eggs	g/(cap*day)	23	26	19	13	8	5	2	*-89%*	*-91%*
**All products**	**g/(cap*day)**	**1867**	**1931**	**1854**	**1794**	**1739**	**1684**	**1627**	***-13%***	***-16%***
Total energy availability	*kcal/(cap*day)*	*2763*	*3028*	*3028*	*3028*	*3028*	*3028*	*3028*	*10%*	*0%*
Total protein availability	*g CP/(cap*day)*[§]	*77*	*83*	*79*	*77*	*76*	*76*	*77*	*0%*	*-7%*
Animal protein/total protein	*Ratio*	*34%*	*38%*	*31%*	*25%*	*20%*	*15%*	*12%*	*-66%*	*-70%*
Energy from proteins/total energy	*Ratio*	*0.111*	*0.110*	*0.104*	*0.102*	*0.100*	*0.100*	*0.102*	*-9%*	*-7%*

x: PPE: primary product equivalents; #: cap: person; +: raw sugar equivalents; *: mainly treenuts, stimulants and spices; §: CP: crude protein;

Table S6 **Daily intake of main food categories per person in kcal energy in the base year, the reference scenario and in scenarios with reduced food-competing feedstuffs (no climate change impacts on yields, 20% yield reduction in livestock due to reduction in food-competing feedstuffs) for constant energy provision in all scenarios**

Food types (PPE)[x]	Unit[#]	Base year (2005-2009)	Supply of food-competing feedstuffs to livestock in scenarios for 2050 [% of base year] 100%	80%	60%	40%	20%	0%	Difference of 0% food-competing feedstuffs scenario to base year	Difference of 0% to 100% food-competing feedstuffs scenario
**Plant products**	**kcal/(cap*day)**	**2314**	**2488**	**2603**	**2692**	**2763**	**2821**	**2868**	***24%***	***15%***
Grains	kcal/(cap*day)	1363	1334	1442	1512	1556	1573	1564	*15%*	*17%*
Starchy roots	kcal/(cap*day)	150	159	168	175	180	183	182	*21%*	*15%*
Oil crops	kcal/(cap*day)	168	241	227	214	201	189	176	*5%*	*-27%*
Legumes	kcal/(cap*day)	190	249	290	337	395	467	561	*195%*	*125%*
Vegetables	kcal/(cap*day)	88	75	71	67	64	59	55	*-37%*	*-27%*
Fruits	kcal/(cap*day)	99	127	120	113	107	100	93	*-6%*	*-27%*
Sugars and sweeteners	kcal/(cap*day)	217	275	260	247	235	224	212	*-3%*	*-23%*
Others*	kcal/(cap*day)	38	27	26	26	25	25	24	*-36%*	*-9%*
**Livestock products**	**kcal/(cap*day)**	**449**	**540**	**425**	**336**	**265**	**207**	**160**	***-64%***	***-70%***
Milk	kcal/(cap*day)	154	193	167	146	128	112	98	*-37%*	*-49%*
Meat	kcal/(cap*day)	230	279	206	150	106	73	46	*-80%*	*-83%*
Non-ruminants meat	kcal/(cap*day)	177	212	149	100	63	35	14	*-92%*	*-94%*
Ruminants meat	kcal/(cap*day)	53	67	57	50	43	38	33	*-39%*	*-51%*
Fish	kcal/(cap*day)	33	32	27	23	19	16	13	*-60%*	*-59%*
Eggs	kcal/(cap*day)	32	36	26	18	12	7	3	*-90%*	*-91%*
**All products**	**kcal/(cap*day)**	**2763**	**3028**	**3028**	**3028**	**3028**	**3028**	**3028**	***10%***	***0%***

x: PPE: primary product equivalents; #: cap: person; *: mainly treenuts, stimulants and spices;

Table S7 Daily intake of main food categories per person in kcal energy in the base year, the reference scenario and in scenarios with reduced food-competing feedstuffs (climate change impacts on yields, 20% yield reduction in livestock due to reduction in food-competing feedstuffs) for constant energy provision in all scenarios

Food types (PPE)[x]	Unit[#]	Base year (2005-2009)	Supply of food-competing feedstuffs to livestock in scenarios for 2050 [% of base year] 100%	80%	60%	40%	20%	0%	Difference of 0% food-competing feedstuffs scenario to base year	Difference of 0% to 100% food-competing feedstuffs scenario
**Plant products**	**kcal/(cap*day)**	**2314**	**2482**	**2596**	**2686**	**2757**	**2815**	**2863**	***24%***	***15%***
Grains	kcal/(cap*day)	1363	1357	1466	1538	1583	1601	1593	*17%*	*17%*
Starchy roots	kcal/(cap*day)	150	154	162	168	172	174	173	*15%*	*12%*
Oil crops	kcal/(cap*day)	168	247	233	220	208	196	184	*9%*	*-26%*
Legumes	kcal/(cap*day)	190	237	276	321	376	446	536	*182%*	*126%*
Vegetables	kcal/(cap*day)	88	64	60	57	54	50	46	*-47%*	*-27%*
Fruits	kcal/(cap*day)	99	125	117	111	105	99	92	*-7%*	*-26%*
Sugars and sweeteners	kcal/(cap*day)	217	270	256	244	234	224	212	*-3%*	*-21%*
Others*	kcal/(cap*day)	38	28	27	27	27	26	26	*-32%*	*-7%*
**Livestock products**	**kcal/(cap*day)**	**449**	**546**	**432**	**342**	**271**	**213**	**165**	***-63%***	***-70%***
Milk	kcal/(cap*day)	154	185	162	142	125	110	96	*-38%*	*-48%*
Meat	kcal/(cap*day)	230	281	206	151	107	74	48	*-79%*	*-83%*
Non-ruminants meat	kcal/(cap*day)	177	217	150	101	64	35	14	*-92%*	*-93%*
Ruminants meat	kcal/(cap*day)	53	63	56	49	44	38	34	*-37%*	*-47%*
Fish	kcal/(cap*day)	33	44	37	31	27	22	18	*-44%*	*-58%*
Eggs	kcal/(cap*day)	32	37	26	18	12	7	3	*-89%*	*-91%*
**All products**	**kcal/(cap*day)**	**2763**	**3028**	**3028**	**3028**	**3028**	**3028**	**3028**	***10%***	***0%***

x: PPE: primary product equivalents; #: cap: person; *: mainly treenuts, stimulants and spices;

Table S8 Daily intake of main food categories per person in g crude protein in the base year, the reference scenario and in scenarios with reduced food-competing feedstuffs (no climate change impacts on yields, 20% yield reduction in livestock due to reduction in food-competing feedstuffs) for constant energy provision in all scenarios

Food types (PPE)[x]	Unit[#]	Base year (2005-2009)	Supply of food-competing feedstuffs to livestock in scenarios for 2050 [% of base year] 100%	80%	60%	40%	20%	0%	Difference of 0% food-competing feedstuffs scenario to base year	Difference of 0% to 100% food-competing feedstuffs scenario
**Plant products**	**g CP/(cap*day)**	**48**	**48**	**52**	**57**	**60**	**64**	**68**	***42%***	***41%***
Grains	g CP/(cap*day)	33	32	34	35	36	36	36	*9%*	*10%*
Starchy roots	g CP/(cap*day)	2	2	2	2	2	2	2	*2%*	*-2%*
Oil crops	g CP/(cap*day)	0	1	1	0	0	0	0	*5%*	*-21%*
Legumes	g CP/(cap*day)	6	7	10	13	16	20	26	*317%*	*242%*
Vegetables	g CP/(cap*day)	4	4	3	3	3	3	3	*-37%*	*-27%*
Fruits	g CP/(cap*day)	1	1	1	1	1	1	1	*-2%*	*-26%*
Sugars and sweeteners	g CP/(cap*day)	0	0	0	0	0	0	0	*34%*	*-11%*
Others*	g CP/(cap*day)	1	0	1	1	1	1	0	*-73%*	*-32%*
**Livestock products**	**g CP/(cap*day)**	**29**	**34**	**27**	**21**	**17**	**13**	**10**	***-64%***	***-69%***
Milk	g CP/(cap*day)	8	9	8	7	6	5	4	*-43%*	*-49%*
Meat	g CP/(cap*day)	14	18	13	10	7	5	4	*-73%*	*-79%*
Non-ruminants meat	g CP/(cap*day)	9	12	8	6	4	2	1	*-91%*	*-93%*
Ruminants meat	g CP/(cap*day)	5	6	5	4	4	3	3	*-40%*	*-50%*
Fish	g CP/(cap*day)	5	5	4	3	3	2	2	*-60%*	*-59%*
Eggs	g CP/(cap*day)	2	3	2	1	1	1	0	*-90%*	*-91%*
**All products**	**g CP/(cap*day)**	**77**	**82**	**79**	**78**	**77**	**77**	**78**	***1%***	***-5%***

x: PPE: primary product equivalents; #: cap: person; CP: crude protein; *: mainly treenuts, stimulants and spices;

Table S9 **Daily intake of main food categories per person in g crude protein in the base year, the reference scenario and in scenarios with reduced food-competing feedstuffs (climate change impacts on yields, 20% yield reduction in livestock due to reduction in food-competing feedstuffs) for constant energy provision in all scenarios**

Food types (PPE)[x]	Unit[#]	Base year (2005-2009)	Supply of food-competing feedstuffs to livestock in scenarios for 2050 [% of base year] 100%	80%	60%	40%	20%	0%	Difference of 0% food-competing feedstuffs scenario to base year	Difference of 0% to 100% food-competing feedstuffs scenario
**Plant products**	**g CP/(cap*day)**	**48**	**48**	**51**	**54**	**58**	**62**	**66**	***38%***	***38%***
Grains	g CP/(cap*day)	33	33	35	36	36	37	36	*10%*	*10%*
Starchy roots	g CP/(cap*day)	2	2	2	2	2	2	2	*-3%*	*-3%*
Oil crops	g CP/(cap*day)	0	1	1	1	1	1	0	*18%*	*-20%*
Legumes	g CP/(cap*day)	6	7	9	12	15	18	23	*277%*	*252%*
Vegetables	g CP/(cap*day)	4	3	3	3	3	2	2	*-47%*	*-27%*
Fruits	g CP/(cap*day)	1	1	1	1	1	1	1	*-5%*	*-25%*
Sugars and sweeteners	g CP/(cap*day)	0	0	0	0	0	0	0	*38%*	*-10%*
Others*	g CP/(cap*day)	1	1	1	1	1	1	1	*-29%*	*-29%*
**Livestock products**	**g CP/(cap*day)**	**29**	**35**	**28**	**23**	**18**	**14**	**11**	***-62%***	***-68%***
Milk	g CP/(cap*day)	8	8	7	7	6	5	4	*-44%*	*-48%*
Meat	g CP/(cap*day)	14	18	13	10	8	6	4	*-72%*	*-78%*
Non-ruminants meat	g CP/(cap*day)	9	12	8	6	4	2	1	*-91%*	*-93%*
Ruminants meat	g CP/(cap*day)	5	6	5	4	4	3	3	*-39%*	*-47%*
Fish	g CP/(cap*day)	5	6	5	5	4	3	3	*-44%*	*-58%*
Eggs	g CP/(cap*day)	2	3	2	1	1	1	0	*-89%*	*-91%*
**All products**	**g CP/(cap*day)**	**77**	**83**	**79**	**77**	**76**	**76**	**77**	***0%***	***-7%***

x: PPE: primary product equivalents; #: cap: person; CP: crude protein; *: mainly treenuts, stimulants and spices;

Table S10 Overview of the main model outputs for all scenarios and sensitivity analyses with constant energy supply for human nutrition not considering climate change impacts on yields (absolute values). FCF: Food-competing feedstuffs.

			Reference	0% livestock yield reduction due reducting FCF					20% livestock yield reduction due reducting FCF					40% livestock yield reduction due reducting FCF				
Indicators	Unit	Base year 2005-2009	Scenario 2050	80% FCF	60% FCF	40% FCF	20% FCF	0% FCF	80% FCF	60% FCF	40% FCF	20% FCF	0% FCF	80% FCF	60% FCF	40% FCF	20% FCF	0% FCF
Human population	billion people	6.67	9.14	9.14	9.14	9.14	9.14	9.14	9.14	9.14	9.14	9.14	9.14	9.14	9.14	9.14	9.14	9.14
Land occupation: Total	billion hectares	4.92	5.01	4.85	4.75	4.67	4.60	4.54	4.87	4.78	4.71	4.64	4.58	4.89	4.81	4.74	4.67	4.61
Land occupation: Crop	billion hectares	1.54	1.63	1.48	1.37	1.29	1.22	1.17	1.50	1.40	1.33	1.26	1.20	1.51	1.43	1.36	1.30	1.23
Land occupation: Grass	billion hectares	3.38	3.38	3.38	3.38	3.38	3.38	3.38	3.38	3.38	3.38	3.38	3.38	3.38	3.38	3.38	3.38	3.38
Number of cattle	billion animals	1.39	1.85	1.74	1.66	1.58	1.51	1.45	1.74	1.66	1.58	1.51	1.45	1.74	1.66	1.58	1.51	1.45
Number of pigs	billion animals	0.92	1.17	0.87	0.63	0.43	0.26	0.11	0.88	0.64	0.44	0.26	0.11	0.90	0.66	0.45	0.27	0.11
Number of chickens	billion animals	17.56	33.85	26.29	19.83	14.33	9.50	5.16	26.60	20.19	14.61	9.66	5.19	26.89	20.52	14.86	9.79	5.20
Number of buffaloes	billion animals	0.18	0.27	0.26	0.26	0.26	0.26	0.26	0.26	0.26	0.26	0.26	0.26	0.26	0.26	0.26	0.26	0.26
Number of goats	billion animals	0.86	1.39	1.33	1.29	1.25	1.22	1.18	1.33	1.29	1.25	1.22	1.18	1.33	1.29	1.25	1.22	1.18
Number of sheep	billion animals	1.10	1.60	1.52	1.47	1.43	1.38	1.34	1.52	1.47	1.43	1.38	1.34	1.52	1.47	1.43	1.38	1.34
Energy intake per person and day	kcal/cap/day	2,763.27	3,028.06	3,028.06	3,028.06	3,028.06	3,028.06	3,028.06	3,028.06	3,028.06	3,028.06	3,028.06	3,028.06	3,028.06	3,028.06	3,028.06	3,028.06	3,028.06
Protein intake per person and day	g Protein/cap/day	76.83	82.48	80.45	79.59	79.39	79.90	81.43	79.30	77.62	76.88	77.07	78.48	78.19	75.79	74.66	74.71	76.19
Protein-Calorie ratio	Ratio	0.11	0.11	0.11	0.11	0.10	0.11	0.11	0.10	0.10	0.10	0.10	0.10	0.10	0.10	0.10	0.10	0.10
Calorie share of livestock products	%	15%	17%	14%	12%	11%	9%	8%	13%	11%	8%	6%	5%	12%	9%	6%	4%	3%
Protein share of livestock products	%	34%	38%	33%	28%	24%	21%	17%	31%	25%	19%	15%	11%	29%	21%	15%	10%	7%
Calorie share of meat	%	8%	9%	7%	6%	5%	3%	2%	7%	5%	4%	2%	2%	6%	4%	3%	2%	1%
Protein share of meat	%	20%	23%	19%	16%	13%	10%	8%	18%	14%	10%	7%	5%	17%	12%	8%	5%	3%
Calorie share of milk	%	6%	6%	6%	6%	6%	5%	5%	6%	5%	4%	4%	3%	5%	4%	3%	2%	2%
Protein share of milk	%	11%	11%	11%	10%	10%	9%	9%	10%	9%	8%	7%	6%	9%	8%	6%	5%	3%
Meat quantity per capita and day	g/cap/day	110.46	135.73	109.30	88.17	70.52	55.31	41.86	101.68	75.63	55.33	39.41	26.90	94.24	63.82	41.74	26.03	15.17
Milk quantity per capita and day	g/cap/day	241.63	274.17	257.67	246.33	236.29	227.31	219.22	237.47	208.50	182.98	160.39	140.30	218.09	173.81	136.48	105.11	78.92
N-surplus	million tonnes N	87.91	121.76	107.78	95.16	84.26	73.96	62.36	109.85	98.35	87.92	77.52	65.16	111.93	101.56	91.59	81.10	67.92
N-surplus per hectare	kg N/ha	18.57	24.98	22.76	20.51	18.45	16.42	14.01	23.10	21.07	19.11	17.07	14.53	23.45	21.63	19.79	17.74	15.05
P-surplus	million tonnes P	47.18	63.95	57.40	51.88	47.08	42.50	37.84	57.95	52.68	47.95	43.29	38.38	58.50	53.47	48.77	44.02	38.85
P-surplus per hectare	kg P/ha	9.97	13.12	12.12	11.18	10.31	9.43	8.50	12.19	11.28	10.42	9.53	8.56	12.26	11.39	10.54	9.63	8.61
GHG emissions	Gt CO_2-eq	11.01	12.78	12.08	11.54	11.11	10.72	10.33	12.14	11.63	11.20	10.82	10.43	12.19	11.71	11.29	10.90	10.50
Non-renewable energy demand	Exajoules	22.58	26.69	23.75	21.57	19.82	18.28	16.68	24.06	22.02	20.33	18.79	17.16	24.35	22.44	20.78	19.22	17.54
Pesticide use	dimensionless	14.07	15.36	14.22	13.32	12.63	12.08	11.61	14.41	13.63	12.99	12.46	12.00	14.60	13.91	13.31	12.78	12.30
Freshwater use	km^3	1,371.32	2,177.70	2,044.80	1,936.00	1,840.22	1,752.85	1,659.29	2,071.48	1,979.25	1,893.11	1,810.56	1,717.78	2,097.36	2,019.47	1,939.86	1,858.67	1,763.48
Deforestation	million ha	8.23	7.18	6.90	6.71	6.59	6.49	6.40	6.94	6.76	6.64	6.55	6.46	6.97	6.81	6.69	6.60	6.50
Soil erosion due to water	billion tonnes	33.71	36.77	35.20	34.08	33.17	32.39	31.66	35.46	34.50	33.68	32.95	32.23	35.72	34.89	34.13	33.41	32.67

Table S11 Overview of the main model outputs for all scenarios and sensitivity analyses with constant energy supply for human nutrition not considering climate change impacts on yields (relative values compared to base year). FCF: Food-competing feedstuffs.

Indicators	Unit	Base year 2005-2009	Reference Scenario 2050	0% livestock yield reduction due reducting FCF					20% livestock yield reduction due reducting FCF					40% livestock yield reduction due reducting FCF				
				80% FCF	60% FCF	40% FCF	20% FCF	0% FCF	80% FCF	60% FCF	40% FCF	20% FCF	0% FCF	80% FCF	60% FCF	40% FCF	20% FCF	0% FCF
Human population	billion people	6.67	137%	137%	137%	137%	137%	137%	137%	137%	137%	137%	137%	137%	137%	137%	137%	137%
Land occupation: Total	billion hectares	4.92	102%	99%	97%	95%	94%	92%	99%	97%	96%	94%	93%	100%	98%	96%	95%	94%
Land occupation: Crop	billion hectares	1.54	106%	96%	89%	84%	80%	76%	97%	91%	86%	82%	78%	98%	93%	88%	84%	80%
Land occupation: Grass	billion hectares	3.38	100%	100%	100%	100%	100%	100%	100%	100%	100%	100%	100%	100%	100%	100%	100%	100%
Number of cattle	billion animals	1.39	133%	125%	119%	114%	109%	104%	125%	119%	114%	109%	104%	125%	119%	114%	109%	104%
Number of pigs	billion animals	0.92	127%	95%	68%	47%	28%	12%	96%	70%	48%	28%	12%	97%	71%	49%	29%	12%
Number of chickens	billion animals	17.56	193%	150%	113%	82%	54%	29%	151%	115%	83%	55%	30%	153%	117%	85%	56%	30%
Number of buffaloes	billion animals	0.18	146%	144%	144%	144%	144%	144%	144%	144%	144%	144%	144%	144%	144%	144%	144%	144%
Number of goats	billion animals	0.86	161%	155%	150%	145%	141%	137%	155%	150%	145%	141%	137%	155%	150%	145%	141%	137%
Number of sheep	billion animals	1.10	146%	139%	134%	130%	126%	122%	139%	134%	130%	126%	122%	139%	134%	130%	126%	122%
Energy intake per person and day	kcal/cap/day	2,763.27	110%	110%	110%	110%	110%	110%	110%	110%	110%	110%	110%	110%	110%	110%	110%	110%
Protein intake per person and day	g Protein/cap/day	76.83	107%	105%	104%	103%	104%	106%	103%	101%	100%	100%	102%	102%	99%	97%	97%	99%
Protein-Calorie ratio	Ratio	0.11	98%	96%	95%	94%	95%	97%	94%	92%	91%	92%	93%	93%	90%	89%	89%	90%
Calorie share of livestock products	%	15%	111%	94%	80%	69%	59%	51%	87%	69%	54%	42%	33%	80%	58%	41%	28%	18%
Protein share of livestock products	%	34%	111%	96%	82%	71%	60%	50%	90%	72%	57%	44%	34%	84%	62%	44%	30%	20%
Calorie share of meat	%	8%	110%	88%	69%	54%	41%	30%	82%	60%	43%	29%	19%	76%	50%	32%	19%	11%
Protein share of meat	%	20%	116%	96%	79%	64%	51%	39%	91%	70%	52%	38%	26%	85%	60%	41%	26%	15%
Calorie share of milk	%	6%	114%	107%	103%	98%	94%	91%	99%	87%	76%	67%	58%	91%	72%	57%	44%	33%
Protein share of milk	%	11%	104%	99%	95%	91%	86%	81%	93%	83%	73%	63%	54%	86%	71%	56%	43%	31%
Meat quantity per capita and day	g/cap/day	110.46	123%	99%	80%	64%	50%	38%	92%	68%	50%	36%	24%	85%	58%	38%	24%	14%
Milk quantity per capita and day	g/cap/day	241.63	113%	107%	102%	98%	94%	91%	98%	86%	76%	66%	58%	90%	72%	56%	43%	33%
N-surplus	million tonnes N	87.91	139%	123%	108%	96%	84%	71%	125%	112%	100%	88%	74%	127%	116%	104%	92%	77%
N-surplus per hectare	kg N/ha	18.57	134%	123%	110%	99%	88%	75%	124%	113%	103%	92%	78%	126%	116%	107%	96%	81%
P-surplus	million tonnes P	47.18	136%	122%	110%	100%	90%	80%	123%	112%	102%	92%	81%	124%	113%	103%	93%	82%
P-surplus per hectare	kg P/ha	9.97	132%	122%	112%	103%	95%	85%	122%	113%	105%	96%	86%	123%	114%	106%	97%	86%
GHG emissions	Gt CO_2-eq	11.01	116%	110%	105%	101%	97%	94%	110%	106%	102%	98%	95%	111%	106%	103%	99%	95%
Non-renewable energy demand	Exajoules	22.58	118%	105%	96%	88%	81%	74%	107%	97%	90%	83%	76%	108%	99%	92%	85%	78%
Pesticide use	dimensionless	14.07	109%	101%	95%	90%	86%	83%	102%	97%	92%	89%	85%	104%	99%	95%	91%	87%
Freshwater use	km^3	1,371.32	159%	149%	141%	134%	128%	121%	151%	144%	138%	132%	125%	153%	147%	141%	136%	129%
Deforestation	million ha	8.23	87%	84%	82%	80%	79%	78%	84%	82%	81%	80%	79%	85%	83%	81%	80%	79%
Soil erosion due to water	billion tonnes	33.71	109%	104%	101%	98%	96%	94%	105%	102%	100%	98%	96%	106%	103%	101%	99%	97%

Table S12 Overview of the main model outputs for all scenarios and sensitivity analyses with constant energy supply for human nutrition not considering climate change impacts on yields (relative values compared to reference scenario). FCF: Food-competing feedstuffs.

Indicators	Unit	Base year 2005-2009	Reference Scenario 2050	0% livestock yield reduction due reducting FCF					20% livestock yield reduction due reducting FCF					40% livestock yield reduction due reducting FCF				
				80% FCF	60% FCF	40% FCF	20% FCF	0% FCF	80% FCF	60% FCF	40% FCF	20% FCF	0% FCF	80% FCF	60% FCF	40% FCF	20% FCF	0% FCF
Human population	billion people	6.67	9.14	100%	100%	100%	100%	100%	100%	100%	100%	100%	100%	100%	100%	100%	100%	100%
Land occupation: Total	billion hectares	4.92	5.01	97%	95%	93%	92%	91%	97%	96%	94%	93%	92%	98%	96%	95%	93%	92%
Land occupation: Crop	billion hectares	1.54	1.63	91%	84%	79%	75%	72%	92%	86%	82%	78%	74%	93%	88%	84%	80%	76%
Land occupation: Grass	billion hectares	3.38	3.38	100%	100%	100%	100%	100%	100%	100%	100%	100%	100%	100%	100%	100%	100%	100%
Number of cattle	billion animals	1.39	1.85	94%	90%	86%	82%	79%	94%	90%	86%	82%	79%	94%	90%	86%	82%	79%
Number of pigs	billion animals	0.92	1.17	74%	54%	37%	22%	9%	75%	55%	38%	22%	9%	77%	56%	38%	23%	9%
Number of chickens	billion animals	17.56	33.85	78%	59%	42%	28%	15%	79%	60%	43%	29%	15%	79%	61%	44%	29%	15%
Number of buffaloes	billion animals	0.18	0.27	98%	98%	98%	98%	98%	98%	98%	98%	98%	98%	98%	98%	98%	98%	98%
Number of goats	billion animals	0.86	1.39	96%	93%	90%	87%	85%	96%	93%	90%	87%	85%	96%	93%	90%	87%	85%
Number of sheep	billion animals	1.10	1.60	95%	92%	89%	87%	84%	95%	92%	89%	87%	84%	95%	92%	89%	87%	84%
Energy intake per person and day	kcal/cap/day	2,763.27	3,028.06	100%	100%	100%	100%	100%	100%	100%	100%	100%	100%	100%	100%	100%	100%	100%
Protein intake per person and day	g Protein/cap/day	76.83	82.48	98%	97%	96%	97%	99%	96%	94%	93%	93%	95%	95%	92%	91%	91%	92%
Protein-Calorie ratio	Ratio	0.11	0.11	98%	97%	96%	97%	99%	96%	94%	93%	93%	95%	95%	92%	91%	91%	92%
Calorie share of livestock products	%	0.15	0.17	85%	73%	62%	54%	46%	79%	62%	49%	38%	29%	73%	52%	37%	25%	17%
Protein share of livestock products	%	0.34	0.38	86%	74%	64%	54%	45%	81%	65%	52%	40%	30%	76%	56%	40%	27%	18%
Calorie share of meat	%	0.08	0.09	79%	63%	49%	37%	27%	74%	54%	39%	27%	17%	69%	46%	29%	18%	10%
Protein share of meat	%	0.20	0.23	83%	68%	56%	44%	34%	78%	60%	45%	33%	23%	74%	52%	35%	22%	13%
Calorie share of milk	%	0.06	0.06	94%	90%	86%	83%	80%	87%	76%	67%	58%	51%	79%	63%	50%	38%	29%
Protein share of milk	%	0.11	0.11	96%	92%	88%	83%	79%	89%	80%	70%	61%	52%	83%	68%	54%	41%	30%
Meat quantity per capita and day	g/cap/day	110.46	135.73	81%	65%	52%	41%	31%	75%	56%	41%	29%	20%	69%	47%	31%	19%	11%
Milk quantity per capita and day	g/cap/day	241.63	274.17	94%	90%	86%	83%	80%	87%	76%	67%	58%	51%	80%	63%	50%	38%	29%
N-surplus	million tonnes N	87.91	121.76	89%	78%	69%	61%	51%	90%	81%	72%	64%	54%	92%	83%	75%	67%	56%
N-surplus per hectare	kg N/ha	18.57	24.98	91%	82%	74%	66%	56%	92%	84%	77%	68%	58%	94%	87%	79%	71%	60%
P-surplus	million tonnes P	47.18	63.95	90%	81%	74%	66%	59%	91%	82%	75%	68%	60%	91%	84%	76%	69%	61%
P-surplus per hectare	kg P/ha	9.97	13.12	92%	85%	79%	72%	65%	93%	86%	79%	73%	65%	93%	87%	80%	73%	66%
GHG emissions	Gt CO_2-eq	11.01	12.78	94%	90%	87%	84%	81%	95%	91%	88%	85%	82%	95%	92%	88%	85%	82%
Non-renewable energy demand	Exajoules	22.58	26.69	89%	81%	74%	68%	63%	90%	82%	76%	70%	64%	91%	84%	78%	72%	66%
Pesticide use	dimensionless	14.07	15.36	93%	87%	82%	79%	76%	94%	89%	85%	81%	78%	95%	91%	87%	83%	80%
Freshwater use	km^3	1,371.32	2,177.70	94%	89%	85%	80%	76%	95%	91%	87%	83%	79%	96%	93%	89%	85%	81%
Deforestation	million ha	8.23	7.18	96%	93%	92%	90%	89%	97%	94%	92%	91%	90%	97%	95%	93%	92%	91%
Soil erosion due to water	billion tonnes	33.71	36.77	96%	93%	90%	88%	86%	96%	94%	92%	90%	88%	97%	95%	93%	91%	89%

Table S13 Overview of the main model outputs for all scenarios and sensitivity analyses with constant energy supply for human nutrition considering climate change impacts on yields (absolute values). FCF: Food-competing feedstuffs.

Indicators	Unit	Base year 2005-2009	Reference Scenario 2050	0% livestock yield reduction due reducting FCF					20% livestock yield reduction due reducting FCF					40% livestock yield reduction due reducting FCF				
				80% FCF	60% FCF	40% FCF	20% FCF	0% FCF	80% FCF	60% FCF	40% FCF	20% FCF	0% FCF	80% FCF	60% FCF	40% FCF	20% FCF	0% FCF
Human population	billion people	6.67	9.14	9.14	9.14	9.14	9.14	9.14	9.14	9.14	9.14	9.14	9.14	9.14	9.14	9.14	9.14	9.14
Land occupation: Total	billion hectares	4.92	5.77	5.57	5.43	5.32	5.23	5.15	5.59	5.47	5.37	5.28	5.21	5.62	5.51	5.41	5.33	5.25
Land occupation: Crop	billion hectares	1.54	2.39	2.19	2.05	1.94	1.85	1.77	2.22	2.09	1.99	1.91	1.83	2.24	2.13	2.04	1.95	1.87
Land occupation: Grass	billion hectares	3.38	3.38	3.38	3.38	3.38	3.38	3.38	3.38	3.38	3.38	3.38	3.38	3.38	3.38	3.38	3.38	3.38
Number of cattle	billion animals	1.39	1.78	1.68	1.60	1.53	1.46	1.40	1.68	1.60	1.53	1.46	1.40	1.68	1.60	1.53	1.46	1.40
Number of pigs	billion animals	0.92	1.19	0.88	0.64	0.43	0.26	0.11	0.89	0.65	0.44	0.27	0.11	0.90	0.66	0.45	0.27	0.11
Number of chickens	billion animals	17.56	34.95	26.73	20.23	14.72	9.92	5.63	27.02	20.56	14.97	10.04	5.61	27.30	20.86	15.18	10.13	5.58
Number of buffaloes	billion animals	0.18	0.26	0.26	0.26	0.26	0.26	0.26	0.26	0.26	0.26	0.26	0.26	0.26	0.26	0.26	0.26	0.26
Number of goats	billion animals	0.86	1.33	1.29	1.25	1.21	1.18	1.14	1.29	1.25	1.21	1.18	1.14	1.29	1.25	1.21	1.18	1.14
Number of sheep	billion animals	1.10	1.52	1.47	1.42	1.38	1.34	1.30	1.47	1.42	1.38	1.34	1.30	1.47	1.42	1.38	1.34	1.30
Energy intake per person and day	kcal/cap/day	2,763.27	3,028.06	3,028.06	3,028.06	3,028.06	3,028.06	3,028.06	3,028.06	3,028.06	3,028.06	3,028.06	3,028.06	3,028.06	3,028.06	3,028.06	3,028.06	3,028.06
Protein intake per person and day	g Protein/cap/day	76.83	82.80	80.38	79.15	78.60	78.74	79.81	79.25	77.21	76.11	75.92	76.86	78.16	75.40	73.91	73.58	74.57
Protein-Calorie ratio	Ratio	0.11	0.11	0.11	0.10	0.10	0.10	0.11	0.10	0.10	0.10	0.10	0.10	0.10	0.10	0.10	0.10	0.10
Calorie share of livestock products	%	15%	17%	14%	12%	10%	9%	8%	13%	10%	8%	6%	5%	12%	9%	6%	4%	3%
Protein share of livestock products	%	34%	38%	33%	28%	24%	21%	17%	31%	25%	20%	15%	12%	29%	21%	15%	10%	7%
Calorie share of meat	%	8%	9%	7%	6%	5%	3%	2%	7%	5%	4%	2%	2%	6%	4%	3%	2%	1%
Protein share of meat	%	20%	23%	19%	16%	13%	10%	8%	18%	14%	10%	8%	5%	17%	12%	8%	5%	3%
Calorie share of milk	%	6%	6%	6%	6%	5%	5%	5%	5%	5%	4%	4%	3%	5%	4%	3%	2%	2%
Protein share of milk	%	11%	11%	11%	10%	10%	9%	9%	10%	9%	8%	7%	6%	9%	8%	6%	5%	3%
Meat quantity per capita and day	g/cap/day	110.46	136.19	108.99	87.67	69.97	54.80	41.46	101.39	75.18	54.87	39.02	26.62	93.95	63.42	41.36	25.74	14.99
Milk quantity per capita and day	g/cap/day	241.63	261.46	248.96	238.01	228.30	219.62	211.81	229.44	201.45	176.80	154.97	135.56	210.72	167.94	131.87	101.55	76.25
N-surplus	million tonnes N	87.91	116.89	101.55	89.78	79.71	70.37	60.30	103.58	92.91	83.28	73.84	63.05	105.63	96.08	86.90	77.34	65.75
N-surplus per hectare	kg N/ha	18.57	20.95	18.84	17.06	15.44	13.85	12.04	19.12	17.53	15.99	14.39	12.46	19.41	18.00	16.55	14.95	12.89
P-surplus	million tonnes P	47.18	60.43	53.95	48.81	44.29	40.10	36.06	54.49	49.57	45.11	40.84	36.57	55.03	50.32	45.89	41.53	37.02
P-surplus per hectare	kg P/ha	9.97	10.83	10.01	9.28	8.58	7.89	7.20	10.06	9.35	8.66	7.96	7.23	10.11	9.43	8.74	8.03	7.26
GHG emissions	Gt CO_2-eq	11.01	14.01	13.21	12.61	12.13	11.70	11.28	13.28	12.72	12.25	11.83	11.40	13.35	12.82	12.36	11.93	11.49
Non-renewable energy demand	Exajoules	22.58	31.24	27.82	25.41	23.49	21.82	20.10	28.17	25.93	24.08	22.43	20.68	28.51	26.41	24.61	22.94	21.14
Pesticide use	dimensionless	14.07	22.59	20.83	19.63	18.71	17.98	17.37	21.10	20.05	19.22	18.52	17.93	21.36	20.45	19.67	18.98	18.36
Freshwater use	km^3	1,371.32	2,122.43	1,983.72	1,879.47	1,788.31	1,705.57	1,616.29	2,009.33	1,920.83	1,838.75	1,760.51	1,671.94	2,034.17	1,959.29	1,883.32	1,806.30	1,715.40
Deforestation	million ha	8.23	9.72	9.29	8.97	8.73	8.52	8.35	9.34	9.06	8.82	8.63	8.44	9.40	9.13	8.91	8.72	8.52
Soil erosion due to water	billion tonnes	33.71	48.98	46.81	45.30	44.08	43.02	42.03	47.19	45.91	44.83	43.86	42.90	47.55	46.47	45.49	44.55	43.58

Table S14 Overview of the main model outputs for all scenarios and sensitivity analyses with constant energy supply for human nutrition considering climate change impacts on yields (relative values compared to base year). FCF: Food-competing feedstuffs.

Indicators	Unit	Base year 2005-2009	Reference Scenario 2050	0% livestock yield reduction due reducting FCF					20% livestock yield reduction due reducting FCF					40% livestock yield reduction due reducting FCF				
				80% FCF	60% FCF	40% FCF	20% FCF	0% FCF	80% FCF	60% FCF	40% FCF	20% FCF	0% FCF	80% FCF	60% FCF	40% FCF	20% FCF	0% FCF
Human population	billion people	6.67	137%	137%	137%	137%	137%	137%	137%	137%	137%	137%	137%	137%	137%	137%	137%	137%
Land occupation: Total	billion hectares	4.92	117%	113%	110%	108%	106%	105%	114%	111%	109%	107%	106%	114%	112%	110%	108%	107%
Land occupation: Crop	billion hectares	1.54	155%	142%	133%	126%	120%	115%	144%	136%	129%	124%	119%	146%	138%	132%	127%	122%
Land occupation: Grass	billion hectares	3.38	100%	100%	100%	100%	100%	100%	100%	100%	100%	100%	100%	100%	100%	100%	100%	100%
Number of cattle	billion animals	1.39	128%	121%	115%	110%	105%	101%	121%	115%	110%	105%	101%	121%	115%	110%	105%	101%
Number of pigs	billion animals	0.92	130%	96%	69%	47%	28%	12%	97%	71%	48%	29%	12%	98%	72%	49%	29%	12%
Number of chickens	billion animals	17.56	199%	152%	115%	84%	57%	32%	154%	117%	85%	57%	32%	156%	119%	86%	58%	32%
Number of buffaloes	billion animals	0.18	139%	139%	139%	139%	139%	139%	139%	139%	139%	139%	139%	139%	139%	139%	139%	139%
Number of goats	billion animals	0.86	155%	150%	145%	141%	136%	133%	150%	145%	141%	136%	133%	150%	145%	141%	136%	133%
Number of sheep	billion animals	1.10	139%	134%	130%	126%	122%	118%	134%	130%	126%	122%	118%	134%	130%	126%	122%	118%
Energy intake per person and day	kcal/cap/day	2,763.27	110%	110%	110%	110%	110%	110%	110%	110%	110%	110%	110%	110%	110%	110%	110%	110%
Protein intake per person and day	g Protein/cap/day	76.83	108%	105%	103%	102%	102%	104%	103%	100%	99%	99%	100%	102%	98%	96%	96%	97%
Protein-Calorie ratio	Ratio	0.11	98%	95%	94%	93%	94%	95%	94%	92%	90%	90%	91%	93%	90%	88%	87%	89%
Calorie share of livestock products	%	15%	110%	93%	79%	68%	58%	50%	86%	68%	53%	41%	32%	80%	57%	40%	27%	18%
Protein share of livestock products	%	34%	111%	96%	83%	71%	61%	51%	91%	73%	58%	45%	34%	85%	63%	45%	30%	20%
Calorie share of meat	%	8%	111%	88%	69%	54%	41%	29%	82%	60%	42%	29%	19%	76%	50%	32%	19%	11%
Protein share of meat	%	20%	118%	98%	80%	65%	52%	40%	92%	71%	53%	38%	27%	87%	61%	41%	26%	16%
Calorie share of milk	%	6%	110%	104%	99%	95%	91%	88%	96%	84%	74%	65%	56%	88%	70%	55%	42%	32%
Protein share of milk	%	11%	100%	98%	94%	90%	86%	81%	91%	82%	72%	63%	54%	85%	70%	56%	43%	31%
Meat quantity per capita and day	g/cap/day	110.46	123%	99%	79%	63%	50%	38%	92%	68%	50%	35%	24%	85%	57%	37%	23%	14%
Milk quantity per capita and day	g/cap/day	241.63	108%	103%	98%	94%	91%	88%	95%	83%	73%	64%	56%	87%	70%	55%	42%	32%
N-surplus	million tonnes N	87.91	133%	116%	102%	91%	80%	69%	118%	106%	95%	84%	72%	120%	109%	99%	88%	75%
N-surplus per hectare	kg N/ha	18.57	113%	101%	92%	83%	75%	65%	103%	94%	86%	77%	67%	105%	97%	89%	80%	69%
P-surplus	million tonnes P	47.18	128%	114%	103%	94%	85%	76%	115%	105%	96%	87%	78%	117%	107%	97%	88%	78%
P-surplus per hectare	kg P/ha	9.97	109%	100%	93%	86%	79%	72%	101%	94%	87%	80%	73%	101%	95%	88%	81%	73%
GHG emissions	Gt CO_2-eq	11.01	127%	120%	115%	110%	106%	102%	121%	116%	111%	107%	104%	121%	116%	112%	108%	104%
Non-renewable energy demand	Exajoules	22.58	138%	123%	113%	104%	97%	89%	125%	115%	107%	99%	92%	126%	117%	109%	102%	94%
Pesticide use	dimensionless	14.07	161%	148%	140%	133%	128%	123%	150%	143%	137%	132%	127%	152%	145%	140%	135%	131%
Freshwater use	km^3	1,371.32	155%	145%	137%	130%	124%	118%	147%	140%	134%	128%	122%	148%	143%	137%	132%	125%
Deforestation	million ha	8.23	118%	113%	109%	106%	104%	101%	114%	110%	107%	105%	103%	114%	111%	108%	106%	104%
Soil erosion due to water	billion tonnes	33.71	145%	139%	134%	131%	128%	125%	140%	136%	133%	130%	127%	141%	138%	135%	132%	129%

Table S15 Overview of the main model outputs for all scenarios and sensitivity analyses with constant energy supply for human nutrition considering climate change impacts on yields (relative values compared to reference scenario). FCF: Food-competing feedstuffs.

				0% livestock yield reduction due reducting FCF					20% livestock yield reduction due reducting FCF					40% livestock yield reduction due reducting FCF				
Indicators	Unit	Base year 2005-2009	Reference Scenario 2050	80% FCF	60% FCF	40% FCF	20% FCF	0% FCF	80% FCF	60% FCF	40% FCF	20% FCF	0% FCF	80% FCF	60% FCF	40% FCF	20% FCF	0% FCF
Human population	billion people	6.67	9.14	100%	100%	100%	100%	100%	100%	100%	100%	100%	100%	100%	100%	100%	100%	100%
Land occupation: Total	billion hectares	4.92	5.77	96%	94%	92%	91%	89%	97%	95%	93%	92%	90%	97%	95%	94%	92%	91%
Land occupation: Crop	billion hectares	1.54	2.39	91%	86%	81%	77%	74%	93%	87%	83%	80%	76%	94%	89%	85%	82%	78%
Land occupation: Grass	billion hectares	3.38	3.38	100%	100%	100%	100%	100%	100%	100%	100%	100%	100%	100%	100%	100%	100%	100%
Number of cattle	billion animals	1.39	1.78	95%	90%	86%	82%	79%	95%	90%	86%	82%	79%	95%	90%	86%	82%	79%
Number of pigs	billion animals	0.92	1.19	74%	53%	36%	22%	9%	75%	54%	37%	22%	9%	76%	55%	38%	23%	9%
Number of chickens	billion animals	17.56	34.95	76%	58%	42%	28%	16%	77%	59%	43%	29%	16%	78%	60%	43%	29%	16%
Number of buffaloes	billion animals	0.18	0.26	100%	100%	100%	100%	100%	100%	100%	100%	100%	100%	100%	100%	100%	100%	100%
Number of goats	billion animals	0.86	1.33	97%	94%	91%	88%	86%	97%	94%	91%	88%	86%	97%	94%	91%	88%	86%
Number of sheep	billion animals	1.10	1.52	97%	93%	91%	88%	85%	97%	93%	91%	88%	85%	97%	93%	91%	88%	85%
Energy intake per person and day	kcal/cap/day	2,763.27	3,028.06	100%	100%	100%	100%	100%	100%	100%	100%	100%	100%	100%	100%	100%	100%	100%
Protein intake per person and day	g Protein/cap/day	76.83	82.80	97%	96%	95%	95%	96%	96%	93%	92%	92%	93%	94%	91%	89%	89%	90%
Protein-Calorie ratio	Ratio	0.11	0.11	97%	96%	95%	95%	96%	96%	93%	92%	92%	93%	94%	91%	89%	89%	90%
Calorie share of livestock products	%	0.15	0.17	85%	72%	62%	53%	45%	78%	62%	48%	38%	29%	72%	52%	36%	25%	16%
Protein share of livestock products	%	0.34	0.38	86%	74%	64%	54%	46%	81%	65%	52%	40%	30%	76%	56%	40%	27%	18%
Calorie share of meat	%	0.08	0.09	79%	62%	48%	37%	26%	73%	53%	38%	26%	17%	68%	45%	29%	17%	10%
Protein share of meat	%	0.20	0.23	83%	68%	55%	44%	34%	78%	60%	45%	33%	23%	73%	52%	35%	22%	13%
Calorie share of milk	%	0.06	0.06	95%	91%	87%	84%	80%	88%	77%	67%	59%	52%	80%	64%	50%	39%	29%
Protein share of milk	%	0.11	0.11	97%	93%	90%	85%	81%	91%	81%	72%	63%	54%	85%	70%	55%	42%	31%
Meat quantity per capita and day	g/cap/day	110.46	136.19	80%	64%	51%	40%	30%	74%	55%	40%	29%	20%	69%	47%	30%	19%	11%
Milk quantity per capita and day	g/cap/day	241.63	261.46	95%	91%	87%	84%	81%	88%	77%	68%	59%	52%	81%	64%	50%	39%	29%
N-surplus	million tonnes N	87.91	116.89	87%	77%	68%	60%	52%	89%	79%	71%	63%	54%	90%	82%	74%	66%	56%
N-surplus per hectare	kg N/ha	18.57	20.95	90%	81%	74%	66%	57%	91%	84%	76%	69%	59%	93%	86%	79%	71%	62%
P-surplus	million tonnes P	47.18	60.43	89%	81%	73%	66%	60%	90%	82%	75%	68%	61%	91%	83%	76%	69%	61%
P-surplus per hectare	kg P/ha	9.97	10.83	92%	86%	79%	73%	66%	93%	86%	80%	73%	67%	93%	87%	81%	74%	67%
GHG emissions	Gt CO_2-eq	11.01	14.01	94%	90%	87%	84%	81%	95%	91%	87%	84%	81%	95%	92%	88%	85%	82%
Non-renewable energy demand	Exajoules	22.58	31.24	89%	81%	75%	70%	64%	90%	83%	77%	72%	66%	91%	85%	79%	73%	68%
Pesticide use	dimensionless	14.07	22.59	92%	87%	83%	80%	77%	93%	89%	85%	82%	79%	95%	91%	87%	84%	81%
Freshwater use	km^3	1,371.32	2,122.43	93%	89%	84%	80%	76%	95%	91%	87%	83%	79%	96%	92%	89%	85%	81%
Deforestation	million ha	8.23	9.72	96%	92%	90%	88%	86%	96%	93%	91%	89%	87%	97%	94%	92%	90%	88%
Soil erosion due to water	billion tonnes	33.71	48.98	96%	92%	90%	88%	86%	96%	94%	92%	90%	88%	97%	95%	93%	91%	89%

2.3 Results for scenarios with constant protein provision in all scenarios for 2050

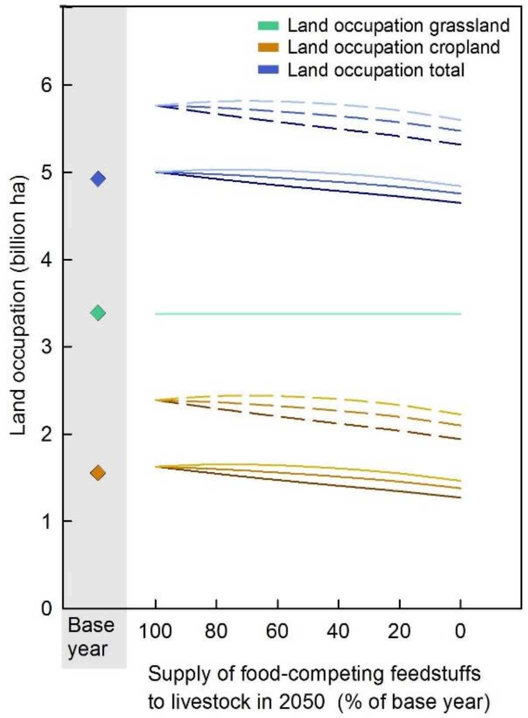

Figure S7 Land occupation by cropland, grassland and total agricultural land for constant protein provision in all scenarios. Results are shown for the base year (indicated by diamonds (♦)), reference scenario, i.e. no reduction in food-competing feedstuffs (= 100%) and with reduced usage of such feedstuffs. Straight lines: no negative impact of climate change on yields assumed; dashed lines: negative impact of climate change on yields assumed; dark lines: no livestock yield reductions due to reduction of food-competing feedstuffs assumed; medium lines: 20% livestock yield reductions due to reduction of food-competing feedstuffs assumed; light lines: 40% livestock yield reductions due to reduction of food-competing feedstuffs assumed

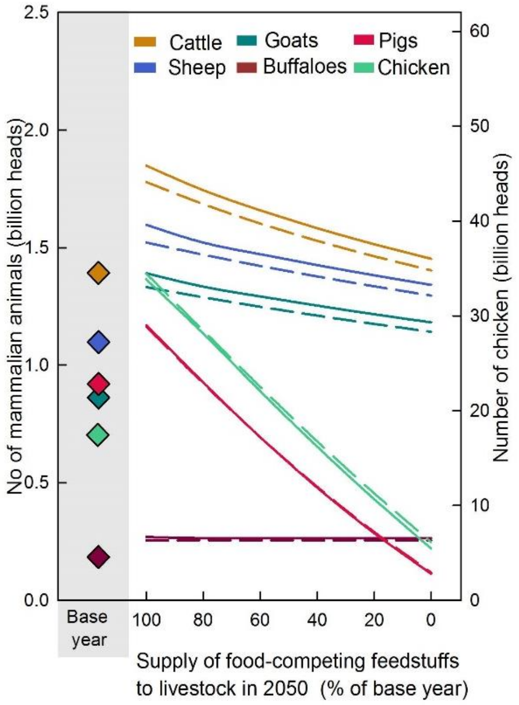

Figure S8 **Livestock numbers for constant protein provision in all scenarios. Results are shown for the base year (indicated by diamonds (♦)), reference scenario, i.e. no reduction in food-competing feedstuffs (= 100%) and with reduced usage of such feedstuffs. Straight lines: no negative impact of climate change on yields assumed; dashed lines: negative impact of climate change on yields assumed; dark lines: no livestock yield reductions due to reduction of food-competing feedstuffs assumed; medium lines: 20% livestock yield reductions due to reduction of food-competing feedstuffs assumed; light lines: 40% livestock yield reductions due to reduction of food-competing feedstuffs assumed**

Table S16 Daily intake of main food categories per person (fresh matter, primary crop equivalents, global average) in the base year, the reference scenario and in scenarios with reduced food-competing feedstuffs (no climate change impacts on yields, 20% yield reduction in livestock due to reduction in food-competing feedstuffs) for constant protein provision in all scenarios

Food types (PPE)[x]	Unit[#]	Base year (2005-2009)	Supply of food-competing feedstuffs to livestock in scenarios for 2050 [% of base year] 100%	80%	60%	40%	20%	0%	Difference of 0% food-competing feedstuffs scenario to base year	Difference of 0% to 100% food-competing feedstuffs scenario
**Plant products**	**g/(cap*day)**	**1442**	**1488**	**1568**	**1621**	**1648**	**1646**	**1612**	***12%***	***8%***
Grains	g/(cap*day)	519	499	558	604	638	656	655	*26%*	*31%*
Starchy roots	g/(cap*day)	185	193	209	221	228	230	226	*22%*	*17%*
Oil crops	g/(cap*day)	74	104	104	101	97	93	86	*17%*	*-17%*
Legumes	g/(cap*day)	42	56	68	83	99	119	142	*235%*	*156%*
Vegetables	g/(cap*day)	343	295	290	281	267	248	225	*-34%*	*-24%*
Fruits	g/(cap*day)	210	260	257	249	239	225	207	*-1%*	*-20%*
Sugars and sweeteners[+]	g/(cap*day)	65	78	78	77	75	72	68	*5%*	*-13%*
Others*	g/(cap*day)	5	4	4	4	4	4	4	*-29%*	*-18%*
**Livestock products**	**g/(cap*day)**	**425**	**484**	**409**	**348**	**294**	**248**	**208**	***-51%***	***-57%***
Milk	g/(cap*day)	242	274	237	208	183	160	140	*-42%*	*-49%*
Meat	g/(cap*day)	110	136	106	81	59	42	27	*-75%*	*-80%*
Non-ruminants meat	g/(cap*day)	77	97	73	51	33	19	7	*-90%*	*-92%*
Ruminants meat	g/(cap*day)	34	39	34	30	26	23	20	*-40%*	*-48%*
Fish	g/(cap*day)	50	48	46	44	43	40	38	*-24%*	*-21%*
Eggs	g/(cap*day)	23	26	20	14	9	5	2	*-90%*	*-91%*
**All products**	**g/(cap*day)**	**1867**	**1972**	**1978**	**1968**	**1942**	**1894**	**1820**	***-3%***	***-8%***
Total energy availability	*kcal/(cap*day)*	*2763*	*3028*	*3214*	*3356*	*3462*	*3526*	*3538*	*28%*	*17%*
Total protein availability	*g CP/(cap*day)* [§]	*77*	*82*	*82*	*82*	*82*	*82*	*82*	*6%*	*0%*
Animal protein/total protein	*Ratio*	*34%*	*38%*	*30%*	*24%*	*19%*	*15%*	*11%*	*-68%*	*-71%*
Energy from proteins/total energy	*Ratio*	*0.111*	*0.108*	*0.102*	*0.098*	*0.095*	*0.093*	*0.093*	*-17%*	*-14%*

x: PPE: primary product equivalents; #: cap: person; +: raw sugar equivalents; *: mainly treenuts, stimulants and spices; §: CP: crude protein;

Table S17 Daily intake of main food categories per person (fresh matter, primary crop equivalents, global average) in the base year, the reference scenario and in scenarios with reduced food-competing feedstuffs (climate change impacts on yields, 20% yield reduction in livestock due to reduction in food-competing feedstuffs) for constant protein provision in all scenarios

			Supply of food-competing feedstuffs to livestock in scenarios for 2050 [% of base year]						Difference of 0% food-competing feedstuffs scenario to base year	Difference of 0% to 100% food-competing feedstuffs scenario
**Food types (PPE)[x]**	**Unit[#]**	**Base year (2005-2009)**	**100%**	**80%**	**60%**	**40%**	**20%**	**0%**		
**Plant products**	**g/(cap*day)**	**1442**	**1439**	**1524**	**1585**	**1621**	**1629**	**1606**	***11%***	***12%***
Grains	g/(cap*day)	519	510	572	621	657	678	680	*31%*	*33%*
Starchy roots	g/(cap*day)	185	187	203	214	221	223	219	*18%*	*17%*
Oil crops	g/(cap*day)	74	106	106	105	101	97	91	*24%*	*-14%*
Legumes	g/(cap*day)	42	53	65	79	95	114	137	*224%*	*159%*
Vegetables	g/(cap*day)	343	252	249	243	233	219	200	*-42%*	*-20%*
Fruits	g/(cap*day)	210	250	248	243	234	222	206	*-2%*	*-18%*
Sugars and sweeteners[+]	g/(cap*day)	65	76	77	76	75	72	68	*6%*	*-10%*
Others*	g/(cap*day)	5	4	4	4	4	4	4	*-25%*	*-15%*
**Livestock products**	**g/(cap*day)**	**425**	**486**	**416**	**356**	**303**	**258**	**219**	***-49%***	***-55%***
Milk	g/(cap*day)	242	261	229	201	177	155	136	*-44%*	*-48%*
Meat	g/(cap*day)	110	134	105	80	59	42	27	*-75%*	*-80%*
Non-ruminants meat	g/(cap*day)	77	97	73	51	34	19	8	*-90%*	*-92%*
Ruminants meat	g/(cap*day)	34	37	32	29	25	22	19	*-42%*	*-47%*
Fish	g/(cap*day)	50	64	62	60	58	56	53	*6%*	*-17%*
Eggs	g/(cap*day)	23	26	20	14	9	6	3	*-88%*	*-90%*
**All products**	**g/(cap*day)**	**1867**	**1925**	**1941**	**1941**	**1925**	**1887**	**1824**	***-2%***	***-5%***
Total energy availability	*kcal/(cap*day)*	*2763*	*3025*	*3223*	*3379*	*3498*	*3575*	*3601*	*30%*	*19%*
Total protein availability	*g CP/(cap*day)[§]*	*77*	*82*	*82*	*82*	*82*	*82*	*82*	*6%*	*0%*
Animal protein/total protein	*Ratio*	*34%*	*38%*	*30%*	*24%*	*19%*	*14%*	*11%*	*-68%*	*-71%*
Energy from proteins/total energy	*Ratio*	*0.111*	*0.108*	*0.102*	*0.097*	*0.094*	*0.092*	*0.091*	*-18%*	*-16%*

x: PPE: primary product equivalents; #: cap: person; +: raw sugar equivalents; *: mainly treenuts, stimulants and spices; §: CP: crude protein;

Table S18 **Daily intake of main food categories per person in kcal energy in the base year, the reference scenario and in scenarios with reduced food-competing feedstuffs (no climate change impacts on yields, 20% yield reduction in livestock due to reduction in food-competing feedstuffs) for constant protein provision in all scenarios**

Food types (PPE)[x]	Unit[#]	Base year (2005-2009)	Supply of food-competing feedstuffs to livestock in scenarios for 2050 [% of base year] 100%	80%	60%	40%	20%	0%	Difference of 0% food-competing feedstuffs scenario to base year	Difference of 0% to 100% food-competing feedstuffs scenario
**Plant products**	**kcal/(cap*day)**	**2314**	**2488**	**2775**	**3004**	**3183**	**3309**	**3372**	***46%***	***36%***
Grains	kcal/(cap*day)	1363	1334	1550	1717	1839	1909	1919	*41%*	*44%*
Starchy roots	kcal/(cap*day)	150	159	174	186	193	195	192	*28%*	*21%*
Oil crops	kcal/(cap*day)	168	241	240	234	225	213	197	*17%*	*-18%*
Legumes	kcal/(cap*day)	190	249	307	372	446	533	637	*235%*	*156%*
Vegetables	kcal/(cap*day)	88	75	74	72	68	63	57	*-35%*	*-24%*
Fruits	kcal/(cap*day)	99	127	126	123	118	111	102	*3%*	*-20%*
Sugars and sweeteners	kcal/(cap*day)	217	275	276	272	266	255	240	*10%*	*-13%*
Others*	kcal/(cap*day)	38	27	27	28	29	28	27	*-28%*	*2%*
**Livestock products**	**kcal/(cap*day)**	**449**	**540**	**439**	**352**	**279**	**217**	**166**	***-63%***	***-69%***
Milk	kcal/(cap*day)	154	193	168	147	129	113	99	*-36%*	*-49%*
Meat	kcal/(cap*day)	230	279	216	161	116	79	50	*-79%*	*-82%*
Non-ruminants meat	kcal/(cap*day)	177	212	158	110	71	39	15	*-92%*	*-93%*
Ruminants meat	kcal/(cap*day)	53	67	58	51	45	40	35	*-35%*	*-48%*
Fish	kcal/(cap*day)	33	32	28	24	21	17	14	*-57%*	*-56%*
Eggs	kcal/(cap*day)	32	36	27	19	13	7	3	*-90%*	*-91%*
**All products**	**kcal/(cap*day)**	**2763**	**3028**	**3214**	**3356**	**3462**	**3526**	**3538**	***28%***	***17%***

x: PPE: primary product equivalents; #: cap: person; *: mainly treenuts, stimulants and spices;

Table S19 **Daily intake of main food categories per person in kcal energy in the base year, the reference scenario and in scenarios with reduced food-competing feedstuffs (climate change impacts on yields, 20% yield reduction in livestock due to reduction in food-competing feedstuffs) for constant protein provision in all scenarios**

Food types (PPE)[x]	Unit[#]	Base year (2005-2009)	Supply of food-competing feedstuffs to livestock in scenarios for 2050 [% of base year] 100%	80%	60%	40%	20%	0%	Difference of 0% food-competing feedstuffs scenario to base year	Difference of 0% to 100% food-competing feedstuffs scenario
**Plant products**	**kcal/(cap*day)**	**2314**	**2485**	**2782**	**3023**	**3216**	**3354**	**3432**	***48%***	***38%***
Grains	kcal/(cap*day)	1363	1357	1582	1761	1893	1972	1991	*46%*	*47%*
Starchy roots	kcal/(cap*day)	150	156	170	181	188	190	187	*25%*	*20%*
Oil crops	kcal/(cap*day)	168	246	247	243	235	225	210	*25%*	*-15%*
Legumes	kcal/(cap*day)	190	238	293	355	427	513	616	*224%*	*159%*
Vegetables	kcal/(cap*day)	88	64	63	62	59	56	51	*-42%*	*-20%*
Fruits	kcal/(cap*day)	99	125	124	122	117	111	103	*4%*	*-17%*
Sugars and sweeteners	kcal/(cap*day)	217	271	273	271	266	257	244	*12%*	*-10%*
Others*	kcal/(cap*day)	38	28	29	30	30	30	30	*-21%*	*7%*
**Livestock products**	**kcal/(cap*day)**	**449**	**540**	**441**	**356**	**282**	**221**	**169**	***-62%***	***-69%***
Milk	kcal/(cap*day)	154	185	162	142	125	110	96	*-38%*	*-48%*
Meat	kcal/(cap*day)	230	276	214	160	115	79	49	*-79%*	*-82%*
Non-ruminants meat	kcal/(cap*day)	177	213	158	111	72	40	16	*-91%*	*-93%*
Ruminants meat	kcal/(cap*day)	53	63	56	49	44	38	34	*-37%*	*-47%*
Fish	kcal/(cap*day)	33	43	38	34	29	24	20	*-39%*	*-54%*
Eggs	kcal/(cap*day)	32	36	27	20	13	8	4	*-88%*	*-90%*
**All products**	**kcal/(cap*day)**	**2763**	**3025**	**3223**	**3379**	**3498**	**3575**	**3601**	***30%***	***19%***

x: PPE: primary product equivalents; #: cap: person; *: mainly treenuts, stimulants and spices;

Table S20 Daily intake of main food categories per person in g crude protein in the base year, the reference scenario and in scenarios with reduced food-competing feedstuffs (no climate change impacts on yields, 20% yield reduction in livestock due to reduction in food-competing feedstuffs) for constant protein provision in all scenarios

Food types (PPE)[x]	Unit[#]	Base year (2005-2009)	Supply of food-competing feedstuffs to livestock in scenarios for 2050 [% of base year] 100%	80%	60%	40%	20%	0%	Difference of 0% food-competing feedstuffs scenario to base year	Difference of 0% to 100% food-competing feedstuffs scenario
**Plant products**	**g CP/(cap*day)**	**48**	**48**	**54**	**59**	**64**	**68**	**71**	***49%***	***48%***
Grains	g CP/(cap*day)	33	32	36	38	40	40	40	*21%*	*22%*
Starchy roots	g CP/(cap*day)	2	2	2	2	2	2	2	*9%*	*5%*
Oil crops	g CP/(cap*day)	0	1	1	1	1	0	0	*13%*	*-16%*
Legumes	g CP/(cap*day)	6	7	10	13	16	20	25	*302%*	*230%*
Vegetables	g CP/(cap*day)	4	4	4	3	3	3	3	*-35%*	*-24%*
Fruits	g CP/(cap*day)	1	1	1	1	1	1	1	*7%*	*-19%*
Sugars and sweeteners	g CP/(cap*day)	0	0	0	0	0	0	0	*35%*	*-10%*
Others*	g CP/(cap*day)	1	0	0	0	0	0	0	*-74%*	*-34%*
**Livestock products**	**g CP/(cap*day)**	**29**	**34**	**28**	**23**	**18**	**14**	**11**	***-63%***	***-68%***
Milk	g CP/(cap*day)	8	9	8	7	6	5	5	*-42%*	*-49%*
Meat	g CP/(cap*day)	14	18	14	11	8	6	4	*-72%*	*-77%*
Non-ruminants meat	g CP/(cap*day)	9	12	9	6	4	2	1	*-90%*	*-92%*
Ruminants meat	g CP/(cap*day)	5	6	5	5	4	4	3	*-37%*	*-48%*
Fish	g CP/(cap*day)	5	5	4	4	3	3	2	*-57%*	*-55%*
Eggs	g CP/(cap*day)	2	3	2	1	1	1	0	*-90%*	*-91%*
**All products**	**g CP/(cap*day)**	**77**	**82**	**82**	**82**	**82**	**82**	**82**	***6%***	***0%***

x: PPE: primary product equivalents; #: cap: person; CP: crude protein; *: mainly treenuts, stimulants and spices;

Table S21 Daily intake of main food categories per person in g crude protein in the base year, the reference scenario and in scenarios with reduced food-competing feedstuffs (climate change impacts on yields, 20% yield reduction in livestock due to reduction in food-competing feedstuffs) for constant protein provision in all scenarios

Food types (PPE)[x]	Unit[#]	Base year (2005-2009)	Supply of food-competing feedstuffs to livestock in scenarios for 2050 [% of base year] 100%	80%	60%	40%	20%	0%	Difference of 0% food-competing feedstuffs scenario to base year	Difference of 0% to 100% food-competing feedstuffs scenario
**Plant products**	**g CP/(cap*day)**	**48**	**47**	**53**	**58**	**63**	**67**	**70**	***48%***	***50%***
Grains	g CP/(cap*day)	33	33	36	39	41	41	41	*25%*	*24%*
Starchy roots	g CP/(cap*day)	2	2	2	2	2	2	2	*7%*	*6%*
Oil crops	g CP/(cap*day)	0	1	1	1	1	1	1	*32%*	*-12%*
Legumes	g CP/(cap*day)	6	7	9	12	15	19	23	*273%*	*246%*
Vegetables	g CP/(cap*day)	4	3	3	3	3	3	2	*-42%*	*-20%*
Fruits	g CP/(cap*day)	1	1	1	1	1	1	1	*7%*	*-16%*
Sugars and sweeteners	g CP/(cap*day)	0	0	0	0	0	0	0	*48%*	*-7%*
Others*	g CP/(cap*day)	1	0	0	0	0	0	0	*-70%*	*-27%*
**Livestock products**	**g CP/(cap*day)**	**29**	**35**	**29**	**24**	**19**	**15**	**12**	***-60%***	***-67%***
Milk	g CP/(cap*day)	8	8	7	7	6	5	4	*-44%*	*-48%*
Meat	g CP/(cap*day)	14	18	14	11	8	6	4	*-72%*	*-77%*
Non-ruminants meat	g CP/(cap*day)	9	12	9	6	4	2	1	*-89%*	*-92%*
Ruminants meat	g CP/(cap*day)	5	6	5	4	4	3	3	*-39%*	*-47%*
Fish	g CP/(cap*day)	5	6	6	5	4	4	3	*-39%*	*-53%*
Eggs	g CP/(cap*day)	2	3	2	2	1	1	0	*-88%*	*-90%*
**All products**	**g CP/(cap*day)**	**77**	**82**	**82**	**82**	**82**	**82**	**82**	***6%***	***0%***

x: PPE: primary product equivalents; #: cap: person; CP: crude protein; *: mainly treenuts, stimulants and spices;

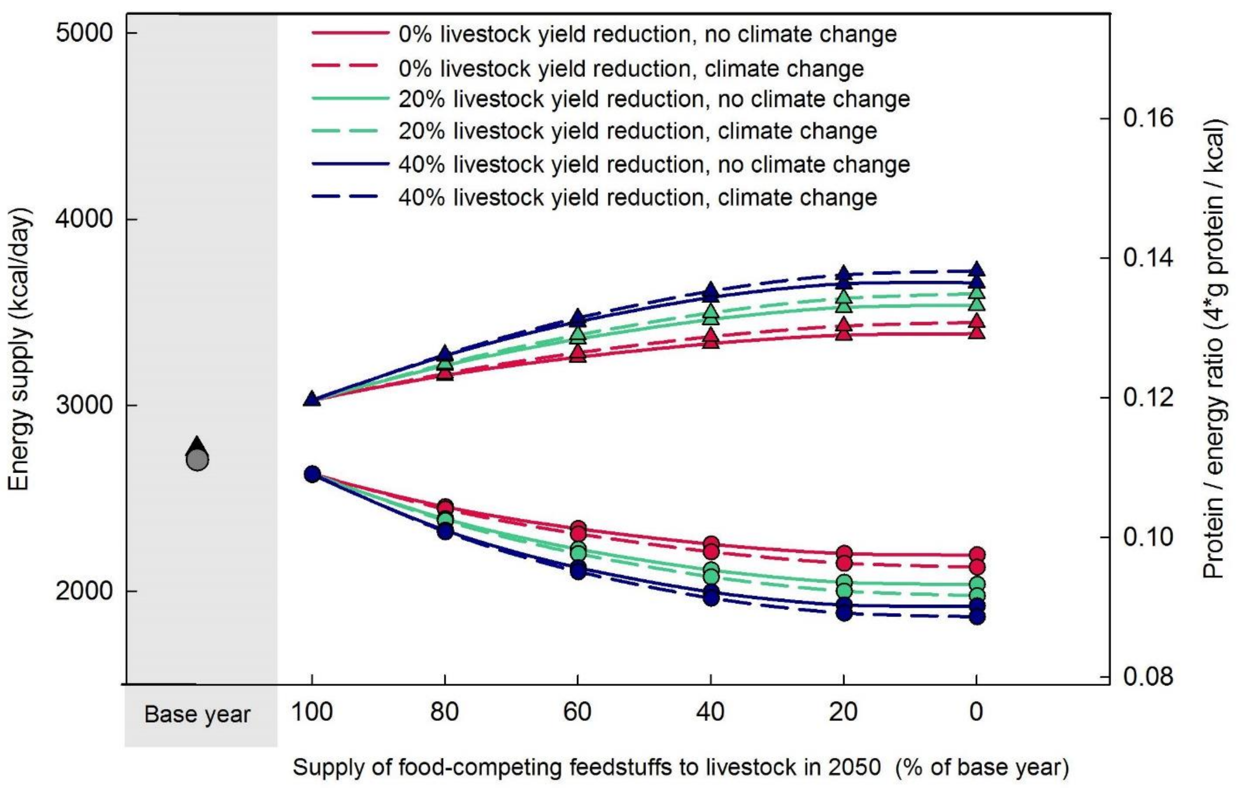

Figure S9 **Daily energy supply per person [kcal per person per day] and protein/calorie ratio in the base year, the reference scenario for 2050 and with reduction of food-competing feedstuffs (global averages).▲ , calorie supply; ●, protein / energy ratio. Black symbols: base year; constant protein provision in all scenarios**

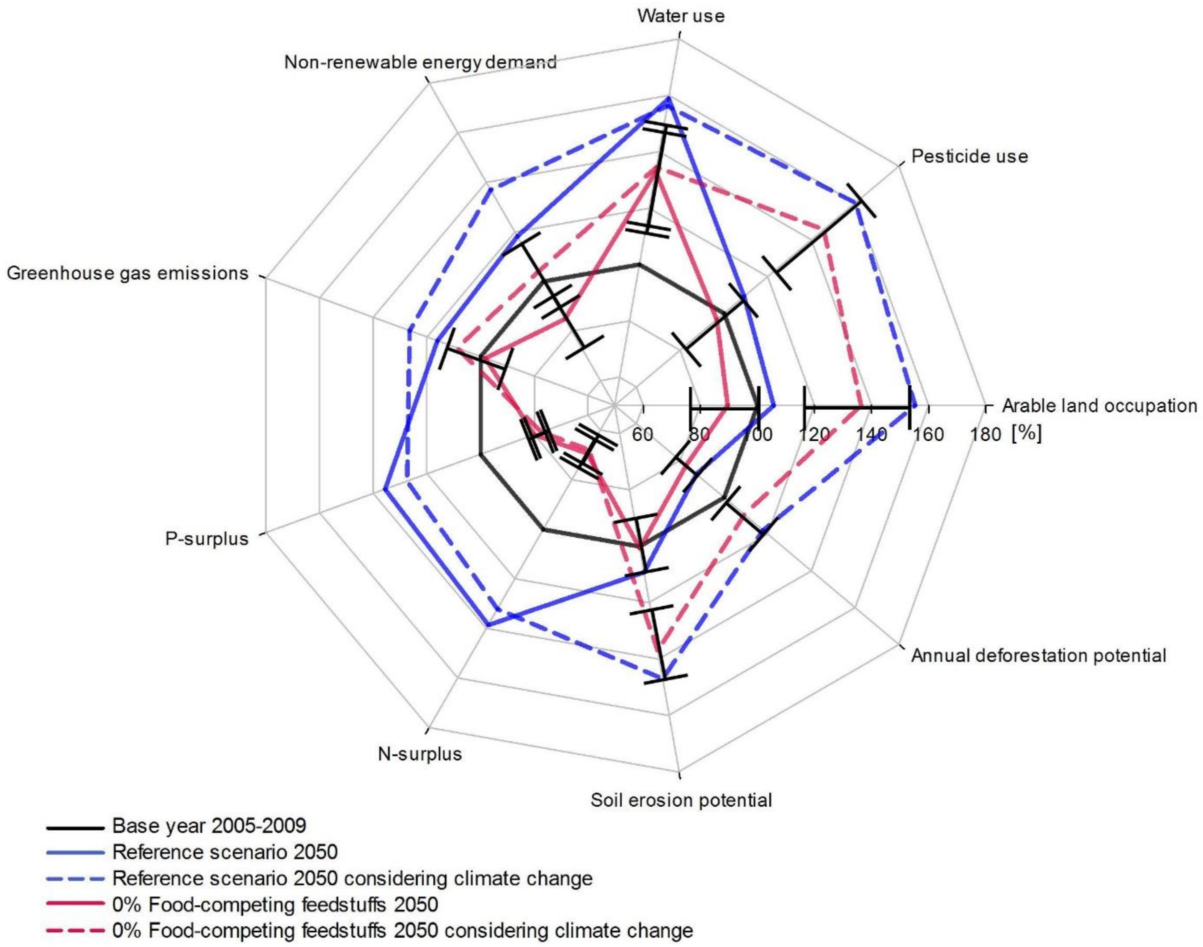

Figure S10 Change of environmental pressures resulting from a reduction in food-competing feedstuffs relative to the base year [%] assuming equal protein provision in the reference scenario and the scenario with 0% food-competing feedstuffs. Solid lines: no negative impact of climate change on yields assumed; dashed lines: negative impact of climate change on yields assumed; black: base year; blue: reference scenario (same level of food-competing feedstuffs assumed for 2050); red: 0% food-competing feedstuffs. Black whiskers represent the range resulting from different assumptions on animal yield reduction (0%-40%) for constant protein provision in all scenarios

Table S22 Overview of the main model outputs for all scenarios and sensitivity analyses with constant protein supply for human nutrition not considering climate change impacts on yields (absolute values). FCF: Food-competing feedstuffs.

			Reference	0% livestock yield reduction due reducting FCF					20% livestock yield reduction due reducting FCF					40% livestock yield reduction due reducting FCF				
		Base year	Scenario															
Indicators	Unit	2005-2009	2050	80% FCF	60% FCF	40% FCF	20% FCF	0% FCF	80% FCF	60% FCF	40% FCF	20% FCF	0% FCF	80% FCF	60% FCF	40% FCF	20% FCF	0% FCF
Human population	billion people	6.67	9.14	9.14	9.14	9.14	9.14	9.14	9.14	9.14	9.14	9.14	9.14	9.14	9.14	9.14	9.14	9.14
Land occupation: Total	billion hectares	4.92	5.01	4.92	4.85	4.79	4.72	4.65	4.98	4.94	4.89	4.83	4.76	5.03	5.02	4.98	4.93	4.84
Land occupation: Crop	billion hectares	1.54	1.63	1.55	1.47	1.41	1.34	1.27	1.60	1.56	1.51	1.45	1.38	1.65	1.64	1.61	1.55	1.46
Land occupation: Grass	billion hectares	3.38	3.38	3.38	3.38	3.38	3.38	3.38	3.38	3.38	3.38	3.38	3.38	3.38	3.38	3.38	3.38	3.38
Number of cattle	billion animals	1.39	1.85	1.74	1.66	1.58	1.51	1.45	1.74	1.66	1.58	1.51	1.45	1.74	1.66	1.58	1.51	1.45
Number of pigs	billion animals	0.92	1.17	0.90	0.66	0.45	0.27	0.11	0.93	0.69	0.48	0.28	0.11	0.96	0.73	0.50	0.30	0.12
Number of chickens	billion animals	17.56	33.85	27.28	20.96	15.25	10.03	5.21	28.14	22.05	16.21	10.67	5.47	28.99	23.10	17.07	11.19	5.64
Number of buffaloes	billion animals	0.18	0.27	0.26	0.26	0.26	0.26	0.26	0.26	0.26	0.26	0.26	0.26	0.26	0.26	0.26	0.26	0.26
Number of goats	billion animals	0.86	1.39	1.33	1.29	1.25	1.22	1.18	1.33	1.29	1.25	1.22	1.18	1.33	1.29	1.25	1.22	1.18
Number of sheep	billion animals	1.10	1.60	1.52	1.47	1.43	1.38	1.34	1.52	1.47	1.43	1.38	1.34	1.52	1.47	1.43	1.38	1.34
Energy intake per person and day	kcal/cap/day	2,763.27	3,028.06	3,161.30	3,259.26	3,332.23	3,377.87	3,385.05	3,214.30	3,356.45	3,461.76	3,526.47	3,538.21	3,266.78	3,449.69	3,579.96	3,653.46	3,659.15
Protein intake per person and day	g Protein/cap/day	76.83	82.48	82.48	82.48	82.48	82.48	82.48	82.48	82.48	82.48	82.48	82.48	82.48	82.48	82.48	82.48	82.48
Protein-Calorie ratio	Ratio	0.11	0.11	0.10	0.10	0.10	0.10	0.10	0.10	0.10	0.10	0.09	0.09	0.10	0.10	0.09	0.09	0.09
Calorie share of livestock products	%	15%	17%	14%	12%	10%	8%	7%	13%	10%	7%	6%	4%	12%	8%	6%	4%	2%
Protein share of livestock products	%	34%	38%	32%	28%	24%	20%	17%	30%	24%	19%	15%	11%	28%	21%	14%	10%	6%
Calorie share of meat	%	8%	9%	7%	6%	4%	3%	2%	7%	5%	3%	2%	1%	6%	4%	3%	1%	1%
Protein share of meat	%	20%	23%	19%	16%	13%	10%	8%	18%	14%	10%	7%	5%	17%	12%	8%	5%	3%
Calorie share of milk	%	6%	6%	6%	5%	5%	5%	5%	5%	4%	4%	3%	3%	5%	4%	3%	2%	2%
Protein share of milk	%	11%	11%	11%	10%	10%	9%	9%	10%	9%	7%	6%	6%	9%	7%	6%	4%	3%
Meat quantity per capita and day	g/cap/day	110.46	135.73	112.61	92.10	73.81	57.30	42.24	106.27	80.76	59.34	41.70	27.47	99.87	69.59	45.74	28.02	15.63
Milk quantity per capita and day	g/cap/day	241.63	274.17	257.67	246.33	236.29	227.31	219.22	237.47	208.50	182.98	160.39	140.30	218.09	173.81	136.48	105.11	78.92
N-surplus	million tonnes N	87.91	121.76	107.66	94.62	83.13	71.92	58.61	110.22	98.42	87.28	75.62	60.93	112.84	102.37	91.59	79.49	63.36
N-surplus per hectare	kg N/ha	18.57	24.98	22.43	19.99	17.79	15.59	12.89	22.73	20.45	18.30	16.03	13.11	23.04	20.94	18.86	16.55	13.41
P-surplus	million tonnes P	47.18	63.95	57.52	51.83	46.77	41.83	36.75	58.52	53.28	48.31	43.22	37.75	59.52	54.70	49.78	44.49	38.58
P-surplus per hectare	kg P/ha	9.97	13.12	11.98	10.95	10.01	9.06	8.08	12.07	11.07	10.13	9.16	8.12	12.15	11.19	10.25	9.26	8.17
GHG emissions	Gt CO_2-eq	11.01	12.78	12.26	11.80	11.39	10.99	10.55	12.42	12.06	11.68	11.28	10.82	12.59	12.31	11.96	11.54	11.03
Non-renewable energy demand	Exajoules	22.58	26.69	24.81	23.06	21.45	19.82	17.87	25.61	24.30	22.87	21.25	19.16	26.40	25.48	24.17	22.48	20.19
Pesticide use	dimensionless	14.07	15.36	14.82	14.21	13.65	13.11	12.54	15.33	15.04	14.65	14.19	13.60	15.83	15.83	15.57	15.11	14.44
Freshwater use	km^3	1,371.32	2,177.70	2,094.75	2,001.93	1,907.25	1,808.20	1,685.95	2,157.35	2,105.40	2,034.51	1,945.95	1,821.96	2,219.05	2,203.97	2,149.89	2,063.33	1,929.77
Deforestation	million ha	8.23	7.18	7.03	6.87	6.77	6.68	6.59	7.14	7.06	6.98	6.89	6.80	7.25	7.24	7.17	7.09	6.96
Soil erosion due to water	billion tonnes	33.71	36.77	35.94	35.08	34.26	33.40	32.39	36.63	36.23	35.66	34.93	33.91	37.32	37.32	36.95	36.24	35.12

Table S23 Overview of the main model outputs for all scenarios and sensitivity analyses with constant protein supply for human nutrition not considering climate change impacts on yields (relative values compared to base year). FCF: Food-competing feedstuffs.

Indicators	Unit	Base year 2005-2009	Reference Scenario 2050	0% livestock yield reduction due reducting FCF					20% livestock yield reduction due reducting FCF					40% livestock yield reduction due reducting FCF				
				80% FCF	60% FCF	40% FCF	20% FCF	0% FCF	80% FCF	60% FCF	40% FCF	20% FCF	0% FCF	80% FCF	60% FCF	40% FCF	20% FCF	0% FCF
Human population	billion people	6.67	137%	137%	137%	137%	137%	137%	137%	137%	137%	137%	137%	137%	137%	137%	137%	137%
Land occupation: Total	billion hectares	4.92	102%	100%	99%	97%	96%	95%	101%	100%	99%	98%	97%	102%	102%	101%	100%	98%
Land occupation: Crop	billion hectares	1.54	106%	100%	96%	91%	87%	83%	104%	101%	98%	94%	90%	107%	107%	104%	101%	95%
Land occupation: Grass	billion hectares	3.38	100%	100%	100%	100%	100%	100%	100%	100%	100%	100%	100%	100%	100%	100%	100%	100%
Number of cattle	billion animals	1.39	133%	125%	119%	114%	109%	104%	125%	119%	114%	109%	104%	125%	119%	114%	109%	104%
Number of pigs	billion animals	0.92	127%	97%	72%	49%	29%	12%	101%	75%	52%	31%	12%	104%	79%	55%	32%	13%
Number of chickens	billion animals	17.56	193%	155%	119%	87%	57%	30%	160%	126%	92%	61%	31%	165%	132%	97%	64%	32%
Number of buffaloes	billion animals	0.18	146%	144%	144%	144%	144%	144%	144%	144%	144%	144%	144%	144%	144%	144%	144%	144%
Number of goats	billion animals	0.86	161%	155%	150%	145%	141%	137%	155%	150%	145%	141%	137%	155%	150%	145%	141%	137%
Number of sheep	billion animals	1.10	146%	139%	134%	130%	126%	122%	139%	134%	130%	126%	122%	139%	134%	130%	126%	122%
Energy intake per person and day	kcal/cap/day	2,763.27	110%	114%	118%	121%	122%	123%	116%	121%	125%	128%	128%	118%	125%	130%	132%	132%
Protein intake per person and day	g Protein/cap/day	76.83	107%	107%	107%	107%	107%	107%	107%	107%	107%	107%	107%	107%	107%	107%	107%	107%
Protein-Calorie ratio	Ratio	0.11	98%	94%	91%	89%	88%	88%	92%	88%	86%	84%	84%	91%	86%	83%	81%	81%
Calorie share of livestock products	%	15%	111%	91%	76%	64%	54%	46%	84%	64%	49%	37%	28%	77%	53%	36%	24%	15%
Protein share of livestock products	%	34%	111%	95%	82%	70%	59%	50%	89%	71%	55%	43%	32%	83%	60%	42%	28%	18%
Calorie share of meat	%	8%	110%	86%	67%	51%	38%	27%	80%	57%	40%	27%	17%	74%	48%	30%	17%	9%
Protein share of meat	%	20%	116%	96%	80%	65%	51%	39%	91%	70%	52%	37%	26%	85%	60%	40%	25%	15%
Calorie share of milk	%	6%	114%	103%	95%	89%	85%	81%	93%	78%	67%	57%	50%	84%	64%	48%	36%	27%
Protein share of milk	%	11%	104%	97%	92%	88%	84%	80%	89%	78%	68%	59%	52%	82%	65%	51%	39%	29%
Meat quantity per capita and day	g/cap/day	110.46	123%	102%	83%	67%	52%	38%	96%	73%	54%	38%	25%	90%	63%	41%	25%	14%
Milk quantity per capita and day	g/cap/day	241.63	113%	107%	102%	98%	94%	91%	98%	86%	76%	66%	58%	90%	72%	56%	43%	33%
N-surplus	million tonnes N	87.91	139%	122%	108%	95%	82%	67%	125%	112%	99%	86%	69%	128%	116%	104%	90%	72%
N-surplus per hectare	kg N/ha	18.57	134%	121%	108%	96%	84%	69%	122%	110%	99%	86%	71%	124%	113%	102%	89%	72%
P-surplus	million tonnes P	47.18	136%	122%	110%	99%	89%	78%	124%	113%	102%	92%	80%	126%	116%	106%	94%	82%
P-surplus per hectare	kg P/ha	9.97	132%	120%	110%	100%	91%	81%	121%	111%	102%	92%	81%	122%	112%	103%	93%	82%
GHG emissions	Gt CO_2-eq	11.01	116%	111%	107%	103%	100%	96%	113%	109%	106%	102%	98%	114%	112%	109%	105%	100%
Non-renewable energy demand	Exajoules	22.58	118%	110%	102%	95%	88%	79%	113%	108%	101%	94%	85%	117%	113%	107%	100%	89%
Pesticide use	dimensionless	14.07	109%	105%	101%	97%	93%	89%	109%	107%	104%	101%	97%	113%	113%	111%	107%	103%
Freshwater use	km^3	1,371.32	159%	153%	146%	139%	132%	123%	157%	154%	148%	142%	133%	162%	161%	157%	150%	141%
Deforestation	million ha	8.23	87%	85%	84%	82%	81%	80%	87%	86%	85%	84%	83%	88%	88%	87%	86%	85%
Soil erosion due to water	billion tonnes	33.71	109%	107%	104%	102%	99%	96%	109%	107%	106%	104%	101%	111%	111%	110%	108%	104%

Table S24 Overview of the main model outputs for all scenarios and sensitivity analyses with constant protein supply for human nutrition not considering climate change impacts on yields (relative values compared to reference scenario). FCF: Food-competing feedstuffs.

Indicators	Unit	Base year 2005-2009	Reference Scenario 2050	0% livestock yield reduction due reducting FCF					20% livestock yield reduction due reducting FCF					40% livestock yield reduction due reducting FCF				
				80% FCF	60% FCF	40% FCF	20% FCF	0% FCF	80% FCF	60% FCF	40% FCF	20% FCF	0% FCF	80% FCF	60% FCF	40% FCF	20% FCF	0% FCF
Human population	billion people	6.67	9.14	100%	100%	100%	100%	100%	100%	100%	100%	100%	100%	100%	100%	100%	100%	100%
Land occupation: Total	billion hectares	4.92	5.01	98%	97%	96%	94%	93%	99%	99%	98%	97%	95%	100%	100%	100%	98%	97%
Land occupation: Crop	billion hectares	1.54	1.63	95%	91%	87%	83%	78%	98%	96%	93%	89%	85%	102%	101%	99%	95%	90%
Land occupation: Grass	billion hectares	3.38	3.38	100%	100%	100%	100%	100%	100%	100%	100%	100%	100%	100%	100%	100%	100%	100%
Number of cattle	billion animals	1.39	1.85	94%	90%	86%	82%	79%	94%	90%	86%	82%	79%	94%	90%	86%	82%	79%
Number of pigs	billion animals	0.92	1.17	77%	56%	38%	23%	9%	79%	59%	41%	24%	10%	82%	62%	43%	26%	10%
Number of chickens	billion animals	17.56	33.85	81%	62%	45%	30%	15%	83%	65%	48%	32%	16%	86%	68%	50%	33%	17%
Number of buffaloes	billion animals	0.18	0.27	98%	98%	98%	98%	98%	98%	98%	98%	98%	98%	98%	98%	98%	98%	98%
Number of goats	billion animals	0.86	1.39	96%	93%	90%	87%	85%	96%	93%	90%	87%	85%	96%	93%	90%	87%	85%
Number of sheep	billion animals	1.10	1.60	95%	92%	89%	87%	84%	95%	92%	89%	87%	84%	95%	92%	89%	87%	84%
Energy intake per person and day	kcal/cap/day	2,763.27	3,028.06	104%	108%	110%	112%	112%	106%	111%	114%	116%	117%	108%	114%	118%	121%	121%
Protein intake per person and day	g Protein/cap/day	76.83	82.48	100%	100%	100%	100%	100%	100%	100%	100%	100%	100%	100%	100%	100%	100%	100%
Protein-Calorie ratio	Ratio	0.11	0.11	96%	93%	91%	90%	89%	94%	90%	87%	86%	86%	93%	88%	85%	83%	83%
Calorie share of livestock products	%	0.15	0.17	83%	69%	58%	49%	41%	76%	58%	44%	34%	25%	70%	48%	32%	22%	14%
Protein share of livestock products	%	0.34	0.38	86%	74%	63%	54%	45%	80%	64%	50%	39%	29%	75%	55%	38%	26%	17%
Calorie share of meat	%	0.08	0.09	78%	61%	47%	35%	24%	73%	52%	36%	24%	15%	67%	44%	27%	16%	8%
Protein share of meat	%	0.20	0.23	83%	69%	56%	44%	34%	78%	60%	45%	32%	22%	74%	52%	34%	22%	13%
Calorie share of milk	%	0.06	0.06	90%	83%	78%	74%	71%	81%	68%	58%	50%	44%	74%	56%	42%	32%	24%
Protein share of milk	%	0.11	0.11	93%	89%	85%	81%	78%	86%	75%	66%	57%	50%	79%	63%	49%	38%	28%
Meat quantity per capita and day	g/cap/day	110.46	135.73	83%	68%	54%	42%	31%	78%	60%	44%	31%	20%	74%	51%	34%	21%	12%
Milk quantity per capita and day	g/cap/day	241.63	274.17	94%	90%	86%	83%	80%	87%	76%	67%	58%	51%	80%	63%	50%	38%	29%
N-surplus	million tonnes N	87.91	121.76	88%	78%	68%	59%	48%	91%	81%	72%	62%	50%	93%	84%	75%	65%	52%
N-surplus per hectare	kg N/ha	18.57	24.98	90%	80%	71%	62%	52%	91%	82%	73%	64%	53%	92%	84%	76%	66%	54%
P-surplus	million tonnes P	47.18	63.95	90%	81%	73%	65%	57%	92%	83%	76%	68%	59%	93%	86%	78%	70%	60%
P-surplus per hectare	kg P/ha	9.97	13.12	91%	83%	76%	69%	62%	92%	84%	77%	70%	62%	93%	85%	78%	71%	62%
GHG emissions	Gt CO_2-eq	11.01	12.78	96%	92%	89%	86%	82%	97%	94%	91%	88%	85%	98%	96%	94%	90%	86%
Non-renewable energy demand	Exajoules	22.58	26.69	93%	86%	80%	74%	67%	96%	91%	86%	80%	72%	99%	95%	91%	84%	76%
Pesticide use	dimensionless	14.07	15.36	97%	93%	89%	85%	82%	100%	98%	95%	92%	89%	103%	103%	101%	98%	94%
Freshwater use	km^3	1,371.32	2,177.70	96%	92%	88%	83%	77%	99%	97%	93%	89%	84%	102%	101%	99%	95%	89%
Deforestation	million ha	8.23	7.18	98%	96%	94%	93%	92%	99%	98%	97%	96%	95%	101%	101%	100%	99%	97%
Soil erosion due to water	billion tonnes	33.71	36.77	98%	95%	93%	91%	88%	100%	99%	97%	95%	92%	101%	101%	100%	99%	96%

Table S25 Overview of the main model outputs for all scenarios and sensitivity analyses with constant protein supply for human nutrition considering climate change impacts on yields (absolute values). FCF: Food-competing feedstuffs.

			Reference	0% livestock yield reduction due reducting FCF					20% livestock yield reduction due reducting FCF					40% livestock yield reduction due reducting FCF				
Indicators	Unit	Base year 2005-2009	Scenario 2050	80% FCF	60% FCF	40% FCF	20% FCF	0% FCF	80% FCF	60% FCF	40% FCF	20% FCF	0% FCF	80% FCF	60% FCF	40% FCF	20% FCF	0% FCF
Human population	billion people	6.67	9.14	9.14	9.14	9.14	9.14	9.14	9.14	9.14	9.14	9.14	9.14	9.14	9.14	9.14	9.14	9.14
Land occupation: Total	billion hectares	4.92	5.77	5.67	5.58	5.50	5.41	5.32	5.74	5.70	5.64	5.57	5.48	5.81	5.81	5.78	5.71	5.60
Land occupation: Crop	billion hectares	1.54	2.39	2.29	2.20	2.12	2.04	1.94	2.36	2.32	2.26	2.19	2.10	2.43	2.43	2.40	2.33	2.22
Land occupation: Grass	billion hectares	3.38	3.38	3.38	3.38	3.38	3.38	3.38	3.38	3.38	3.38	3.38	3.38	3.38	3.38	3.38	3.38	3.38
Number of cattle	billion animals	1.39	1.78	1.68	1.60	1.53	1.46	1.40	1.68	1.60	1.53	1.46	1.40	1.68	1.60	1.53	1.46	1.40
Number of pigs	billion animals	0.92	1.16	0.89	0.66	0.45	0.27	0.11	0.92	0.69	0.48	0.29	0.12	0.95	0.73	0.51	0.30	0.12
Number of chickens	billion animals	17.56	34.48	27.61	21.43	15.81	10.65	5.89	28.45	22.49	16.74	11.27	6.13	29.28	23.51	17.57	11.76	6.28
Number of buffaloes	billion animals	0.18	0.26	0.26	0.26	0.26	0.26	0.26	0.26	0.26	0.26	0.26	0.26	0.26	0.26	0.26	0.26	0.26
Number of goats	billion animals	0.86	1.33	1.29	1.25	1.21	1.18	1.14	1.29	1.25	1.21	1.18	1.14	1.29	1.25	1.21	1.18	1.14
Number of sheep	billion animals	1.10	1.52	1.47	1.42	1.38	1.34	1.30	1.47	1.42	1.38	1.34	1.30	1.47	1.42	1.38	1.34	1.30
Energy intake per person and day	kcal/cap/day	2,763.27	3,024.61	3,171.47	3,284.00	3,370.02	3,427.23	3,446.67	3,222.96	3,379.40	3,498.24	3,575.47	3,600.93	3,273.87	3,470.79	3,615.11	3,702.09	3,722.85
Protein intake per person and day	g Protein/cap/day	76.83	82.48	82.48	82.48	82.48	82.48	82.48	82.48	82.48	82.48	82.48	82.48	82.48	82.48	82.48	82.48	82.48
Protein-Calorie ratio	Ratio	0.11	0.11	0.10	0.10	0.10	0.10	0.10	0.10	0.10	0.09	0.09	0.09	0.10	0.10	0.09	0.09	0.09
Calorie share of livestock products	%	15%	17%	14%	11%	10%	8%	7%	13%	10%	7%	6%	4%	12%	8%	5%	4%	2%
Protein share of livestock products	%	34%	38%	32%	28%	24%	20%	17%	30%	24%	19%	14%	11%	28%	21%	14%	10%	6%
Calorie share of meat	%	8%	9%	7%	6%	4%	3%	2%	7%	5%	3%	2%	1%	6%	4%	2%	1%	1%
Protein share of meat	%	20%	23%	19%	16%	13%	10%	8%	18%	14%	10%	7%	5%	17%	12%	8%	5%	3%
Calorie share of milk	%	6%	6%	6%	5%	5%	5%	4%	5%	4%	4%	3%	3%	5%	3%	3%	2%	1%
Protein share of milk	%	11%	11%	10%	10%	9%	9%	9%	10%	8%	7%	6%	6%	9%	7%	5%	4%	3%
Meat quantity per capita and day	g/cap/day	110.46	134.21	111.50	91.41	73.40	57.10	42.20	105.16	80.10	58.96	41.52	27.45	98.78	68.96	45.41	27.88	15.61
Milk quantity per capita and day	g/cap/day	241.63	261.46	248.96	238.01	228.30	219.62	211.81	229.44	201.45	176.80	154.97	135.56	210.72	167.94	131.87	101.55	76.25
N-surplus	million tonnes N	87.91	116.14	101.17	89.30	78.90	68.87	57.31	103.62	92.99	82.93	72.50	59.70	106.14	96.82	87.16	76.33	62.16
N-surplus per hectare	kg N/ha	18.57	20.83	18.44	16.53	14.81	13.11	11.10	18.66	16.87	15.18	13.43	11.25	18.89	17.23	15.61	13.82	11.46
P-surplus	million tonnes P	47.18	60.09	54.11	48.99	44.34	39.90	35.50	55.04	50.35	45.80	41.23	36.48	55.98	51.69	47.20	42.45	37.30
P-surplus per hectare	kg P/ha	9.97	10.78	9.87	9.07	8.32	7.60	6.88	9.91	9.13	8.38	7.64	6.87	9.96	9.20	8.45	7.69	6.88
GHG emissions	Gt CO_2-eq	11.01	13.92	13.41	12.95	12.52	12.10	11.62	13.60	13.26	12.89	12.48	11.97	13.79	13.55	13.22	12.81	12.26
Non-renewable energy demand	Exajoules	22.58	30.92	28.92	27.16	25.53	23.87	21.85	29.81	28.58	27.19	25.58	23.45	30.70	29.93	28.71	27.04	24.71
Pesticide use	dimensionless	14.07	22.52	21.71	20.98	20.31	19.66	18.93	22.40	22.13	21.73	21.20	20.48	23.09	23.24	23.03	22.52	21.70
Freshwater use	km^3	1,371.32	2,142.14	2,061.80	1,984.36	1,904.01	1,818.29	1,708.23	2,122.56	2,085.70	2,029.76	1,955.68	1,845.31	2,182.45	2,182.27	2,143.84	2,072.90	1,954.19
Deforestation	million ha	8.23	9.69	9.49	9.28	9.07	8.86	8.64	9.65	9.55	9.41	9.23	8.99	9.82	9.82	9.72	9.55	9.27
Soil erosion due to water	billion tonnes	33.71	49.51	48.37	47.31	46.26	45.14	43.79	49.37	48.99	48.37	47.48	46.17	50.36	50.59	50.29	49.48	48.06

Table S26 Overview of the main model outputs for all scenarios and sensitivity analyses with constant protein supply for human nutrition considering climate change impacts on yields (relative values compared to base year). FCF: Food-competing feedstuffs.

Indicators	Unit	Base year 2005-2009	Reference Scenario 2050	0% livestock yield reduction due reducting FCF					20% livestock yield reduction due reducting FCF					40% livestock yield reduction due reducting FCF				
				80% FCF	60% FCF	40% FCF	20% FCF	0% FCF	80% FCF	60% FCF	40% FCF	20% FCF	0% FCF	80% FCF	60% FCF	40% FCF	20% FCF	0% FCF
Human population	billion people	6.67	137%	137%	137%	137%	137%	137%	137%	137%	137%	137%	137%	137%	137%	137%	137%	137%
Land occupation: Total	billion hectares	4.92	117%	115%	113%	112%	110%	108%	117%	116%	115%	113%	111%	118%	118%	117%	116%	114%
Land occupation: Crop	billion hectares	1.54	155%	149%	143%	138%	132%	126%	153%	151%	147%	143%	136%	158%	158%	156%	151%	144%
Land occupation: Grass	billion hectares	3.38	100%	100%	100%	100%	100%	100%	100%	100%	100%	100%	100%	100%	100%	100%	100%	100%
Number of cattle	billion animals	1.39	128%	121%	115%	110%	105%	101%	121%	115%	110%	105%	101%	121%	115%	110%	105%	101%
Number of pigs	billion animals	0.92	126%	97%	72%	49%	30%	12%	100%	75%	52%	31%	13%	103%	79%	55%	33%	13%
Number of chickens	billion animals	17.56	196%	157%	122%	90%	61%	34%	162%	128%	95%	64%	35%	167%	134%	100%	67%	36%
Number of buffaloes	billion animals	0.18	139%	139%	139%	139%	139%	139%	139%	139%	139%	139%	139%	139%	139%	139%	139%	139%
Number of goats	billion animals	0.86	155%	150%	145%	141%	136%	133%	150%	145%	141%	136%	133%	150%	145%	141%	136%	133%
Number of sheep	billion animals	1.10	139%	134%	130%	126%	122%	118%	134%	130%	126%	122%	118%	134%	130%	126%	122%	118%
Energy intake per person and day	kcal/cap/day	2,763.27	109%	115%	119%	122%	124%	125%	117%	122%	127%	129%	130%	118%	126%	131%	134%	135%
Protein intake per person and day	g Protein/cap/day	76.83	107%	107%	107%	107%	107%	107%	107%	107%	107%	107%	107%	107%	107%	107%	107%	107%
Protein-Calorie ratio	Ratio	0.11	98%	94%	90%	88%	87%	86%	92%	88%	85%	83%	82%	91%	85%	82%	80%	80%
Calorie share of livestock products	%	15%	109%	90%	75%	63%	52%	44%	83%	63%	48%	36%	27%	76%	52%	35%	23%	15%
Protein share of livestock products	%	34%	111%	95%	82%	70%	59%	50%	89%	71%	55%	43%	32%	83%	60%	42%	28%	18%
Calorie share of meat	%	8%	110%	85%	66%	51%	38%	26%	79%	57%	39%	26%	16%	74%	48%	29%	17%	9%
Protein share of meat	%	20%	117%	97%	80%	65%	52%	39%	92%	70%	52%	37%	26%	86%	60%	40%	25%	15%
Calorie share of milk	%	6%	110%	99%	92%	85%	81%	77%	90%	75%	64%	55%	47%	81%	61%	46%	35%	26%
Protein share of milk	%	11%	101%	95%	90%	86%	82%	79%	88%	76%	67%	58%	50%	81%	64%	50%	38%	28%
Meat quantity per capita and day	g/cap/day	110.46	122%	101%	83%	66%	52%	38%	95%	73%	53%	38%	25%	89%	62%	41%	25%	14%
Milk quantity per capita and day	g/cap/day	241.63	108%	103%	98%	94%	91%	88%	95%	83%	73%	64%	56%	87%	70%	55%	42%	32%
N-surplus	million tonnes N	87.91	132%	115%	102%	90%	78%	65%	118%	106%	94%	82%	68%	121%	110%	99%	87%	71%
N-surplus per hectare	kg N/ha	18.57	112%	99%	89%	80%	71%	60%	100%	91%	82%	72%	61%	102%	93%	84%	74%	62%
P-surplus	million tonnes P	47.18	127%	115%	104%	94%	85%	75%	117%	107%	97%	87%	77%	119%	110%	100%	90%	79%
P-surplus per hectare	kg P/ha	9.97	108%	99%	91%	83%	76%	69%	99%	92%	84%	77%	69%	100%	92%	85%	77%	69%
GHG emissions	Gt CO_2-eq	11.01	126%	122%	118%	114%	110%	105%	124%	120%	117%	113%	109%	125%	123%	120%	116%	111%
Non-renewable energy demand	Exajoules	22.58	137%	128%	120%	113%	106%	97%	132%	127%	120%	113%	104%	136%	133%	127%	120%	109%
Pesticide use	dimensionless	14.07	160%	154%	149%	144%	140%	135%	159%	157%	154%	151%	146%	164%	165%	164%	160%	154%
Freshwater use	km^3	1,371.32	156%	150%	145%	139%	133%	125%	155%	152%	148%	143%	135%	159%	159%	156%	151%	143%
Deforestation	million ha	8.23	118%	115%	113%	110%	108%	105%	117%	116%	114%	112%	109%	119%	119%	118%	116%	113%
Soil erosion due to water	billion tonnes	33.71	147%	144%	140%	137%	134%	130%	146%	145%	144%	141%	137%	149%	150%	149%	147%	143%

Table S27 Overview of the main model outputs for all scenarios and sensitivity analyses with constant protein supply for human nutrition considering climate change impacts on yields (relative values compared to reference scenario). FCF: Food-competing feedstuffs.

Indicators	Unit	Base year 2005-2009	Reference Scenario 2050	0% livestock yield reduction due reducting FCF					20% livestock yield reduction due reducting FCF					40% livestock yield reduction due reducting FCF				
				80% FCF	60% FCF	40% FCF	20% FCF	0% FCF	80% FCF	60% FCF	40% FCF	20% FCF	0% FCF	80% FCF	60% FCF	40% FCF	20% FCF	0% FCF
Human population	billion people	6.67	9.14	100%	100%	100%	100%	100%	100%	100%	100%	100%	100%	100%	100%	100%	100%	100%
Land occupation: Total	billion hectares	4.92	5.77	98%	97%	95%	94%	92%	100%	99%	98%	97%	95%	101%	101%	100%	99%	97%
Land occupation: Crop	billion hectares	1.54	2.39	96%	92%	89%	85%	81%	99%	97%	95%	92%	88%	102%	102%	100%	98%	93%
Land occupation: Grass	billion hectares	3.38	3.38	100%	100%	100%	100%	100%	100%	100%	100%	100%	100%	100%	100%	100%	100%	100%
Number of cattle	billion animals	1.39	1.78	95%	90%	86%	82%	79%	95%	90%	86%	82%	79%	95%	90%	86%	82%	79%
Number of pigs	billion animals	0.92	1.16	77%	57%	39%	24%	10%	79%	60%	41%	25%	10%	82%	62%	44%	26%	10%
Number of chickens	billion animals	17.56	34.48	80%	62%	46%	31%	17%	83%	65%	49%	33%	18%	85%	68%	51%	34%	18%
Number of buffaloes	billion animals	0.18	0.26	100%	100%	100%	100%	100%	100%	100%	100%	100%	100%	100%	100%	100%	100%	100%
Number of goats	billion animals	0.86	1.33	97%	94%	91%	88%	86%	97%	94%	91%	88%	86%	97%	94%	91%	88%	86%
Number of sheep	billion animals	1.10	1.52	97%	93%	91%	88%	85%	97%	93%	91%	88%	85%	97%	93%	91%	88%	85%
Energy intake per person and day	kcal/cap/day	2,763.27	3,024.61	105%	109%	111%	113%	114%	107%	112%	116%	118%	119%	108%	115%	120%	122%	123%
Protein intake per person and day	g Protein/cap/day	76.83	82.48	100%	100%	100%	100%	100%	100%	100%	100%	100%	100%	100%	100%	100%	100%	100%
Protein-Calorie ratio	Ratio	0.11	0.11	95%	92%	90%	88%	88%	94%	90%	86%	85%	84%	92%	87%	84%	82%	81%
Calorie share of livestock products	%	0.15	0.17	82%	69%	58%	48%	40%	76%	58%	44%	33%	25%	70%	48%	32%	21%	14%
Protein share of livestock products	%	0.34	0.38	86%	74%	63%	53%	45%	81%	64%	50%	38%	29%	75%	55%	38%	26%	16%
Calorie share of meat	%	0.08	0.09	78%	61%	46%	34%	24%	72%	52%	36%	24%	15%	67%	43%	27%	16%	8%
Protein share of meat	%	0.20	0.23	83%	69%	56%	44%	34%	78%	60%	45%	32%	22%	74%	52%	34%	22%	12%
Calorie share of milk	%	0.06	0.06	91%	84%	78%	74%	71%	82%	69%	58%	50%	43%	74%	56%	42%	32%	24%
Protein share of milk	%	0.11	0.11	94%	89%	85%	81%	78%	87%	76%	66%	58%	50%	80%	63%	49%	38%	28%
Meat quantity per capita and day	g/cap/day	110.46	134.21	83%	68%	55%	43%	31%	78%	60%	44%	31%	20%	74%	51%	34%	21%	12%
Milk quantity per capita and day	g/cap/day	241.63	261.46	95%	91%	87%	84%	81%	88%	77%	68%	59%	52%	81%	64%	50%	39%	29%
N-surplus	million tonnes N	87.91	116.14	87%	77%	68%	59%	49%	89%	80%	71%	62%	51%	91%	83%	75%	66%	54%
N-surplus per hectare	kg N/ha	18.57	20.83	89%	79%	71%	63%	53%	90%	81%	73%	64%	54%	91%	83%	75%	66%	55%
P-surplus	million tonnes P	47.18	60.09	90%	82%	74%	66%	59%	92%	84%	76%	69%	61%	93%	86%	79%	71%	62%
P-surplus per hectare	kg P/ha	9.97	10.78	92%	84%	77%	70%	64%	92%	85%	78%	71%	64%	92%	85%	78%	71%	64%
GHG emissions	Gt CO_2-eq	11.01	13.92	96%	93%	90%	87%	83%	98%	95%	93%	90%	86%	99%	97%	95%	92%	88%
Non-renewable energy demand	Exajoules	22.58	30.92	94%	88%	83%	77%	71%	96%	92%	88%	83%	76%	99%	97%	93%	87%	80%
Pesticide use	dimensionless	14.07	22.52	96%	93%	90%	87%	84%	99%	98%	97%	94%	91%	103%	103%	102%	100%	96%
Freshwater use	km^3	1,371.32	2,142.14	96%	93%	89%	85%	80%	99%	97%	95%	91%	86%	102%	102%	100%	97%	91%
Deforestation	million ha	8.23	9.69	98%	96%	94%	91%	89%	100%	99%	97%	95%	93%	101%	101%	100%	99%	96%
Soil erosion due to water	billion tonnes	33.71	49.51	98%	96%	93%	91%	88%	100%	99%	98%	96%	93%	102%	102%	102%	100%	97%

2.4 Results for scenarios with constant land use in all scenarios for 2050

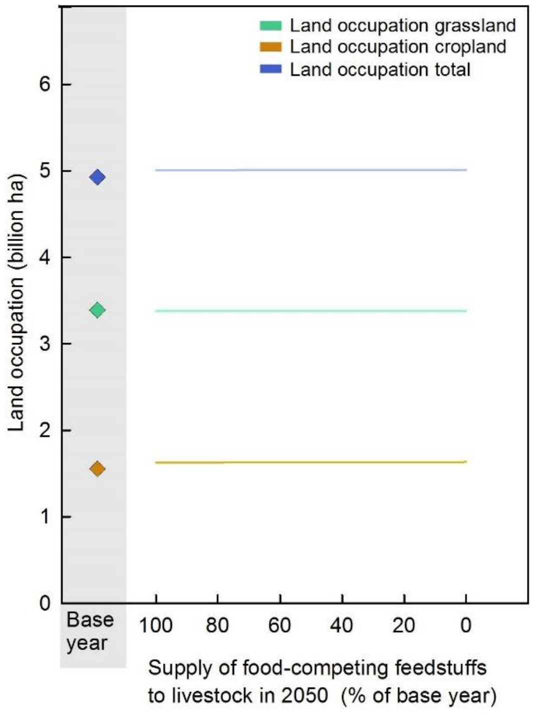

Figure S11 Land occupation by cropland, grassland and total agricultural land for constant land use in all scenarios. Results are shown for the base year (indicated by diamonds (♦)), reference scenario, i.e. no reduction in food-competing feedstuffs (= 100%) and with reduced usage of such feedstuffs. Straight lines: no negative impact of climate change on yields assumed; dashed lines: negative impact of climate change on yields assumed; dark lines: no livestock yield reductions due to reduction of food-competing feedstuffs assumed; medium lines: 20% livestock yield reductions due to reduction of food-competing feedstuffs assumed; light lines: 40% livestock yield reductions due to reduction of food-competing feedstuffs assumed

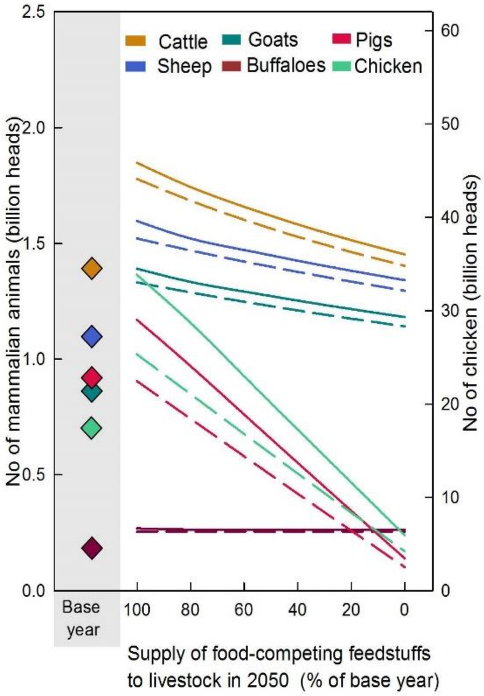

Figure S12 Livestock numbers for constant land use in all scenarios. Results are shown for the base year (indicated by diamonds (♦)), reference scenario, i.e. no reduction in food-competing feedstuffs (= 100%) and with reduced usage of such feedstuffs. Straight lines: no negative impact of climate change on yields assumed; dashed lines: negative impact of climate change on yields assumed; dark lines: no livestock yield reductions due to reduction of food-competing feedstuffs assumed; medium lines: 20% livestock yield reductions due to reduction of food-competing feedstuffs assumed; light lines: 40% livestock yield reductions due to reduction of food-competing feedstuffs assumed

Table S28 Daily intake of main food categories per person (fresh matter, primary crop equivalents, global average) in the base year, the reference scenario and in scenarios with reduced food-competing feedstuffs (no climate change impacts on yields, 20% yield reduction in livestock due to reduction in food-competing feedstuffs) for constant land use in all scenarios

Food types (PPE)[x]	Unit[#]	Base year (2005-2009)	Supply of food-competing feedstuffs to livestock in scenarios for 2050 [% of base year]						Difference of 0% food-competing feedstuffs scenario to base year	Difference of 0% to 100% food-competing feedstuffs scenario
			100%	80%	60%	40%	20%	0%		
**Plant products**	**g/(cap*day)**	**1442**	**1488**	**1600**	**1704**	**1798**	**1880**	**1954**	***36%***	***31%***
Grains	g/(cap*day)	519	499	567	632	691	742	786	*51%*	*58%*
Starchy roots	g/(cap*day)	185	193	213	233	250	265	277	*50%*	*43%*
Oil crops	g/(cap*day)	74	104	105	105	103	102	99	*34%*	*-5%*
Legumes	g/(cap*day)	42	56	70	87	109	137	174	*310%*	*213%*
Vegetables	g/(cap*day)	343	295	300	302	301	296	288	*-16%*	*-2%*
Fruits	g/(cap*day)	210	260	262	262	260	255	248	*18%*	*-4%*
Sugars and sweeteners[+]	g/(cap*day)	65	78	79	79	79	79	78	*21%*	*0%*
Others*	g/(cap*day)	5	4	4	4	4	4	4	*-15%*	*-1%*
**Livestock products**	**g/(cap*day)**	**425**	**484**	**414**	**355**	**303**	**258**	**219**	***-48%***	***-55%***
Milk	g/(cap*day)	242	274	237	208	183	160	140	*-42%*	*-49%*
Meat	g/(cap*day)	110	136	108	84	63	44	29	*-74%*	*-79%*
Non-ruminants meat	g/(cap*day)	77	97	75	54	36	21	8	*-89%*	*-91%*
Ruminants meat	g/(cap*day)	34	39	34	30	26	23	20	*-40%*	*-48%*
Fish	g/(cap*day)	50	48	48	48	48	48	48	*-4%*	*0%*
Eggs	g/(cap*day)	23	26	20	15	10	6	3	*-88%*	*-89%*
**All products**	**g/(cap*day)**	**1867**	**1972**	**2014**	**2059**	**2101**	**2138**	**2173**	***16%***	***10%***
Total energy availability	*kcal/(cap*day)*	*2763*	*3028*	*3269*	*3511*	*3754*	*4000*	*4262*	*54%*	*41%*
Total protein availability	*g CP/(cap*day)* [§]	*77*	*82*	*84*	*87*	*90*	*95*	*102*	*32%*	*24%*
Animal protein/total protein	*Ratio*	*34%*	*38%*	*30%*	*24%*	*18%*	*13%*	*9%*	*-73%*	*-76%*
Energy from proteins/total energy	*Ratio*	*0.111*	*0.108*	*0.103*	*0.099*	*0.096*	*0.095*	*0.096*	*-14%*	*-12%*

x: PPE: primary product equivalents; #: cap: person; +: raw sugar equivalents; *: mainly treenuts, stimulants and spices; §: CP: crude protein;

Table S29 Daily intake of main food categories per person (fresh matter, primary crop equivalents, global average) in the base year, the reference scenario and in scenarios with reduced food-competing feedstuffs (climate change impacts on yields, 20% yield reduction in livestock due to reduction in food-competing feedstuffs) for constant land use in all scenarios

			Supply of food-competing feedstuffs to livestock in scenarios for 2050 [% of base year]							
**Food types (PPE)[x]**	**Unit[#]**	**Base year (2005-2009)**	**100%**	**80%**	**60%**	**40%**	**20%**	**0%**	**Difference of 0% food-competing feedstuffs scenario to base year**	**Difference of 0% to 100% food-competing feedstuffs scenario**
**Plant products**	**g/(cap*day)**	**1442**	**1002**	**1084**	**1160**	**1229**	**1290**	**1345**	***-7%***	***34%***
Grains	g/(cap*day)	519	354	408	459	506	547	582	*12%*	*64%*
Starchy roots	g/(cap*day)	185	122	135	147	157	166	173	*-7%*	*41%*
Oil crops	g/(cap*day)	74	74	75	74	73	72	70	*-5%*	*-5%*
Legumes	g/(cap*day)	42	36	46	58	73	93	118	*178%*	*226%*
Vegetables	g/(cap*day)	343	184	187	188	188	184	180	*-48%*	*-2%*
Fruits	g/(cap*day)	210	176	178	178	176	173	168	*-20%*	*-5%*
Sugars and sweeteners[+]	g/(cap*day)	65	52	52	52	52	52	51	*-21%*	*-1%*
Others*	g/(cap*day)	5	3	3	3	3	3	3	*-39%*	*-1%*
**Livestock products**	**g/(cap*day)**	**425**	**440**	**381**	**330**	**284**	**245**	**211**	***-50%***	***-52%***
Milk	g/(cap*day)	242	261	229	201	177	155	136	*-44%*	*-48%*
Meat	g/(cap*day)	110	111	89	70	53	38	26	*-77%*	*-77%*
Non-ruminants meat	g/(cap*day)	77	75	57	41	27	16	6	*-92%*	*-92%*
Ruminants meat	g/(cap*day)	34	37	32	29	25	22	19	*-42%*	*-47%*
Fish	g/(cap*day)	50	48	48	48	48	48	48	*-4%*	*0%*
Eggs	g/(cap*day)	23	19	15	11	7	4	2	*-92%*	*-90%*
**All products**	**g/(cap*day)**	**1867**	**1442**	**1465**	**1490**	**1514**	**1535**	**1556**	***-17%***	***8%***
Total energy availability	*kcal/(cap*day)*	*2763*	*2172*	*2342*	*2513*	*2685*	*2857*	*3039*	*10%*	*40%*
Total protein availability	*g CP/(cap*day)[§]*	*77*	*62*	*63*	*64*	*66*	*69*	*73*	*-5%*	*18%*
Animal protein/total protein	*Ratio*	*34%*	*44%*	*36%*	*28%*	*22%*	*16%*	*12%*	*-65%*	*-73%*
Energy from proteins/total energy	*Ratio*	*0.111*	*0.114*	*0.108*	*0.102*	*0.098*	*0.097*	*0.096*	*-14%*	*-16%*

x: PPE: primary product equivalents; #: cap: person; +: raw sugar equivalents; *: mainly treenuts, stimulants and spices; §: CP: crude protein;

Table S30 **Daily intake of main food categories per person in kcal energy in the base year, the reference scenario and in scenarios with reduced food-competing feedstuffs (no climate change impacts on yields, 20% yield reduction in livestock due to reduction in food-competing feedstuffs) for constant land use in all scenarios**

Food types (PPE)[x]	Unit[#]	Base year (2005-2009)	Supply of food-competing feedstuffs to livestock in scenarios for 2050 [% of base year] 100%	80%	60%	40%	20%	0%	Difference of 0% food-competing feedstuffs scenario to base year	Difference of 0% to 100% food-competing feedstuffs scenario
**Plant products**	**kcal/(cap*day)**	**2314**	**2488**	**2823**	**3148**	**3464**	**3773**	**4089**	***77%***	***64%***
Grains	kcal/(cap*day)	1363	1334	1577	1803	2009	2188	2339	*72%*	*75%*
Starchy roots	kcal/(cap*day)	150	159	178	195	211	224	234	*56%*	*47%*
Oil crops	kcal/(cap*day)	168	241	245	244	242	238	233	*38%*	*-4%*
Legumes	kcal/(cap*day)	190	249	313	391	488	613	781	*310%*	*213%*
Vegetables	kcal/(cap*day)	88	75	76	77	77	75	73	*-17%*	*-3%*
Fruits	kcal/(cap*day)	99	127	128	129	127	125	122	*23%*	*-4%*
Sugars and sweeteners	kcal/(cap*day)	217	275	278	280	280	279	275	*27%*	*0%*
Treenuts	kcal/(cap*day)	38	27	29	30	30	31	32	*-16%*	*20%*
**Livestock products**	**kcal/(cap*day)**	**449**	**540**	**446**	**363**	**290**	**227**	**173**	***-62%***	***-68%***
Milk	kcal/(cap*day)	154	193	168	147	129	113	99	*-36%*	*-49%*
Meat	kcal/(cap*day)	230	279	221	169	124	85	52	*-77%*	*-81%*
Non-ruminants meat	kcal/(cap*day)	177	212	163	118	79	45	17	*-90%*	*-92%*
Ruminants meat	kcal/(cap*day)	53	67	58	51	45	40	35	*-35%*	*-48%*
Fish	kcal/(cap*day)	33	32	29	26	23	20	18	*-47%*	*-45%*
Eggs	kcal/(cap*day)	32	36	28	21	14	8	4	*-88%*	*-89%*
**All products**	**kcal/(cap*day)**	**2763**	**3028**	**3269**	**3511**	**3754**	**4000**	**4262**	***54%***	***41%***

x: PPE: primary product equivalents; #: cap: person; *: mainly treenuts, stimulants and spices;

Table S31 **Daily intake of main food categories per person in kcal energy in the base year, the reference scenario and in scenarios with reduced food-competing feedstuffs (climate change impacts on yields, 20% yield reduction in livestock due to reduction in food-competing feedstuffs) for constant land use in all scenarios**

Food types (PPE)[x]	Unit[#]	Base year (2005-2009)	Supply of food-competing feedstuffs to livestock in scenarios for 2050 [% of base year] 100%	80%	60%	40%	20%	0%	Difference of 0% food-competing feedstuffs scenario to base year	Difference of 0% to 100% food-competing feedstuffs scenario
**Plant products**	**kcal/(cap*day)**	**2314**	**1701**	**1950**	**2190**	**2423**	**2649**	**2877**	***24%***	***69%***
Grains	kcal/(cap*day)	1363	936	1122	1296	1454	1591	1709	*25%*	*83%*
Starchy roots	kcal/(cap*day)	150	100	112	123	132	140	146	*-3%*	*45%*
Oil crops	kcal/(cap*day)	168	168	170	169	168	165	161	*-4%*	*-4%*
Legumes	kcal/(cap*day)	190	163	208	262	329	416	530	*178%*	*226%*
Vegetables	kcal/(cap*day)	88	46	47	47	47	46	45	*-49%*	*-3%*
Fruits	kcal/(cap*day)	99	86	87	87	86	85	82	*-17%*	*-5%*
Sugars and sweeteners	kcal/(cap*day)	217	182	184	185	185	183	181	*-17%*	*-1%*
Others*	kcal/(cap*day)	38	20	20	21	22	23	22	*-41%*	*15%*
**Livestock products**	**kcal/(cap*day)**	**449**	**471**	**392**	**323**	**262**	**208**	**162**	***-64%***	***-66%***
Milk	kcal/(cap*day)	154	185	162	142	125	110	96	*-38%*	*-48%*
Meat	kcal/(cap*day)	230	227	180	139	103	72	46	*-80%*	*-80%*
Non-ruminants meat	kcal/(cap*day)	177	164	124	90	60	34	13	*-93%*	*-92%*
Ruminants meat	kcal/(cap*day)	53	63	56	49	44	38	34	*-37%*	*-47%*
Fish	kcal/(cap*day)	33	32	29	26	23	20	18	*-47%*	*-45%*
Eggs	kcal/(cap*day)	32	27	21	15	10	6	3	*-92%*	*-90%*
**All products**	**kcal/(cap*day)**	**2763**	**2172**	**2342**	**2513**	**2685**	**2857**	**3039**	***10%***	***40%***

x: PPE: primary product equivalents; #: cap: person; *: mainly treenuts, stimulants and spices;

Table S32 Daily intake of main food categories per person in g crude protein in the base year, the reference scenario and in scenarios with reduced food-competing feedstuffs (no climate change impacts on yields, 20% yield reduction in livestock due to reduction in food-competing feedstuffs) for constant land use in all scenarios

			Supply of food-competing feedstuffs to livestock in scenarios for 2050 [% of base year]						Difference of 0% food-competing feedstuffs scenario to base year	Difference of 0% to 100% food-competing feedstuffs scenario
Food types (PPE)[x]	Unit[#]	Base year (2005-2009)	100%	80%	60%	40%	20%	0%		
**Plant products**	**g CP/(cap*day)**	**48**	**48**	**56**	**64**	**71**	**80**	**90**	***90%***	***88%***
Grains	g CP/(cap*day)	33	32	36	40	43	46	48	*46%*	*47%*
Starchy roots	g CP/(cap*day)	2	2	2	2	3	3	3	*34%*	*29%*
Oil crops	g CP/(cap*day)	0	1	1	1	1	1	1	*28%*	*-4%*
Legumes	g CP/(cap*day)	6	7	11	14	19	25	33	*445%*	*347%*
Vegetables	g CP/(cap*day)	4	4	4	4	4	4	3	*-16%*	*-3%*
Fruits	g CP/(cap*day)	1	1	1	1	1	1	1	*28%*	*-3%*
Sugars and sweeteners	g CP/(cap*day)	0	0	0	0	0	0	0	*51%*	*1%*
Others*	g CP/(cap*day)	1	0	1	1	1	1	1	*4%*	*162%*
**Livestock products**	**g CP/(cap*day)**	**29**	**34**	**28**	**23**	**19**	**15**	**12**	***-61%***	***-66%***
Milk	g CP/(cap*day)	8	9	8	7	6	5	5	*-42%*	*-49%*
Meat	g CP/(cap*day)	14	18	14	11	8	6	4	*-71%*	*-77%*
Non-ruminants meat	g CP/(cap*day)	9	12	9	7	4	3	1	*-89%*	*-91%*
Ruminants meat	g CP/(cap*day)	5	6	5	5	4	4	3	*-37%*	*-48%*
Fish	g CP/(cap*day)	5	5	4	4	3	3	3	*-46%*	*-45%*
Eggs	g CP/(cap*day)	2	3	2	2	1	1	0	*-88%*	*-89%*
**All products**	**g CP/(cap*day)**	**77**	**82**	**84**	**87**	**90**	**95**	**102**	***32%***	***24%***

x: PPE: primary product equivalents; #: cap: person; CP: crude protein; *: mainly treenuts, stimulants and spices;

Table S33 Daily intake of main food categories per person in g crude protein in the base year, the reference scenario and in scenarios with reduced food-competing feedstuffs (climate change impacts on yields, 20% yield reduction in livestock due to reduction in food-competing feedstuffs) for constant land use in all scenarios

			Supply of food-competing feedstuffs to livestock in scenarios for 2050 [% of base year]						Difference of 0% food-competing feedstuffs scenario to base year	Difference of 0% to 100% food-competing feedstuffs scenario
Food types (PPE)[x]	Unit[#]	Base year (2005-2009)	100%	80%	60%	40%	20%	0%		
**Plant products**	**g CP/(cap*day)**	**48**	**32**	**38**	**43**	**49**	**55**	**62**	***30%***	***93%***
Grains	g CP/(cap*day)	33	23	26	28	31	33	35	*5%*	*53%*
Starchy roots	g CP/(cap*day)	2	1	2	2	2	2	2	*-14%*	*29%*
Oil crops	g CP/(cap*day)	0	0	0	0	0	0	0	*-7%*	*-5%*
Legumes	g CP/(cap*day)	6	4	7	9	12	16	22	*255%*	*398%*
Vegetables	g CP/(cap*day)	4	2	2	2	2	2	2	*-49%*	*-3%*
Fruits	g CP/(cap*day)	1	1	1	1	1	1	1	*-16%*	*-4%*
Sugars and sweeteners	g CP/(cap*day)	0	0	0	0	0	0	0	*-1%*	*1%*
Others*	g CP/(cap*day)	1	0	1	0	0	1	1	*-54%*	*58%*
**Livestock products**	**g CP/(cap*day)**	**29**	**30**	**25**	**21**	**17**	**14**	**11**	***-63%***	***-63%***
Milk	g CP/(cap*day)	8	8	7	7	6	5	4	*-44%*	*-48%*
Meat	g CP/(cap*day)	14	15	12	9	7	5	4	*-73%*	*-74%*
Non-ruminants meat	g CP/(cap*day)	9	9	7	5	3	2	1	*-92%*	*-92%*
Ruminants meat	g CP/(cap*day)	5	6	5	4	4	3	3	*-39%*	*-47%*
Fish	g CP/(cap*day)	5	5	4	4	3	3	3	*-46%*	*-45%*
Eggs	g CP/(cap*day)	2	2	2	1	1	0	0	*-92%*	*-90%*
**All products**	**g CP/(cap*day)**	**77**	**62**	**63**	**64**	**66**	**69**	**73**	***-5%***	***18%***

x: PPE: primary product equivalents; #: cap: person; CP: crude protein; *: mainly treenuts, stimulants and spices;

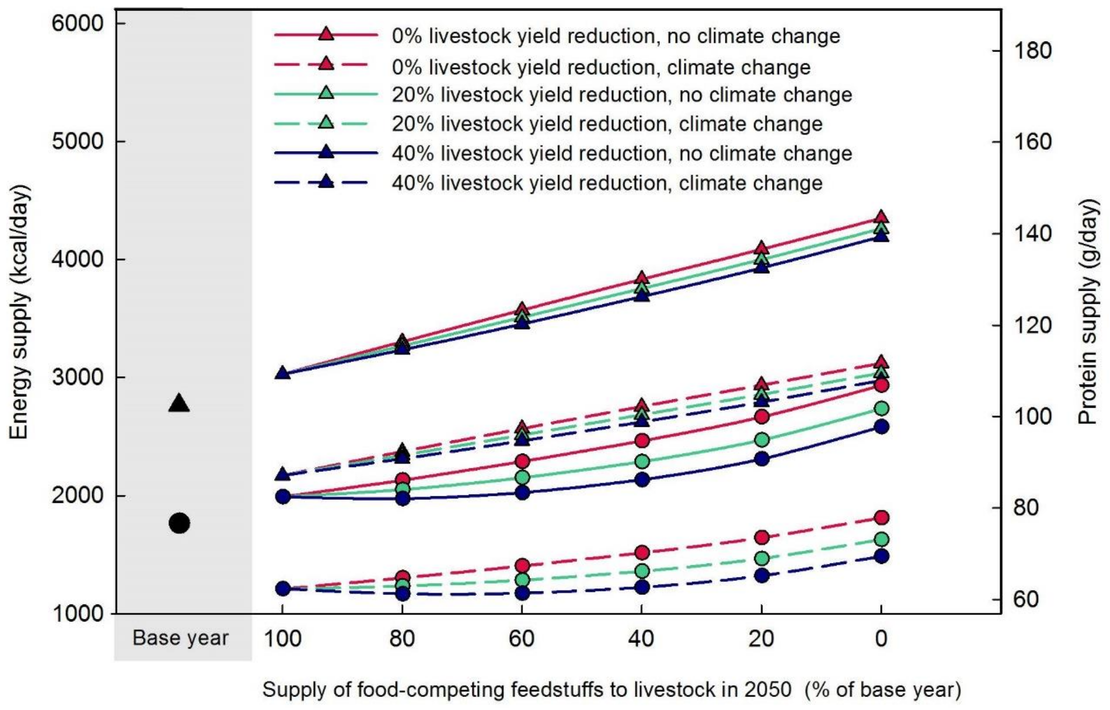

Figure S13 Daily energy supply [kcal per person per day] and protein supply [g crude protein per person per day] per person in the base year, the reference scenario for 2050 and with reduction of food-competing feedstuffs (global averages).▲ , calorie supply; ● protein supply; Black symbols: base year; constant land use in all scenarios

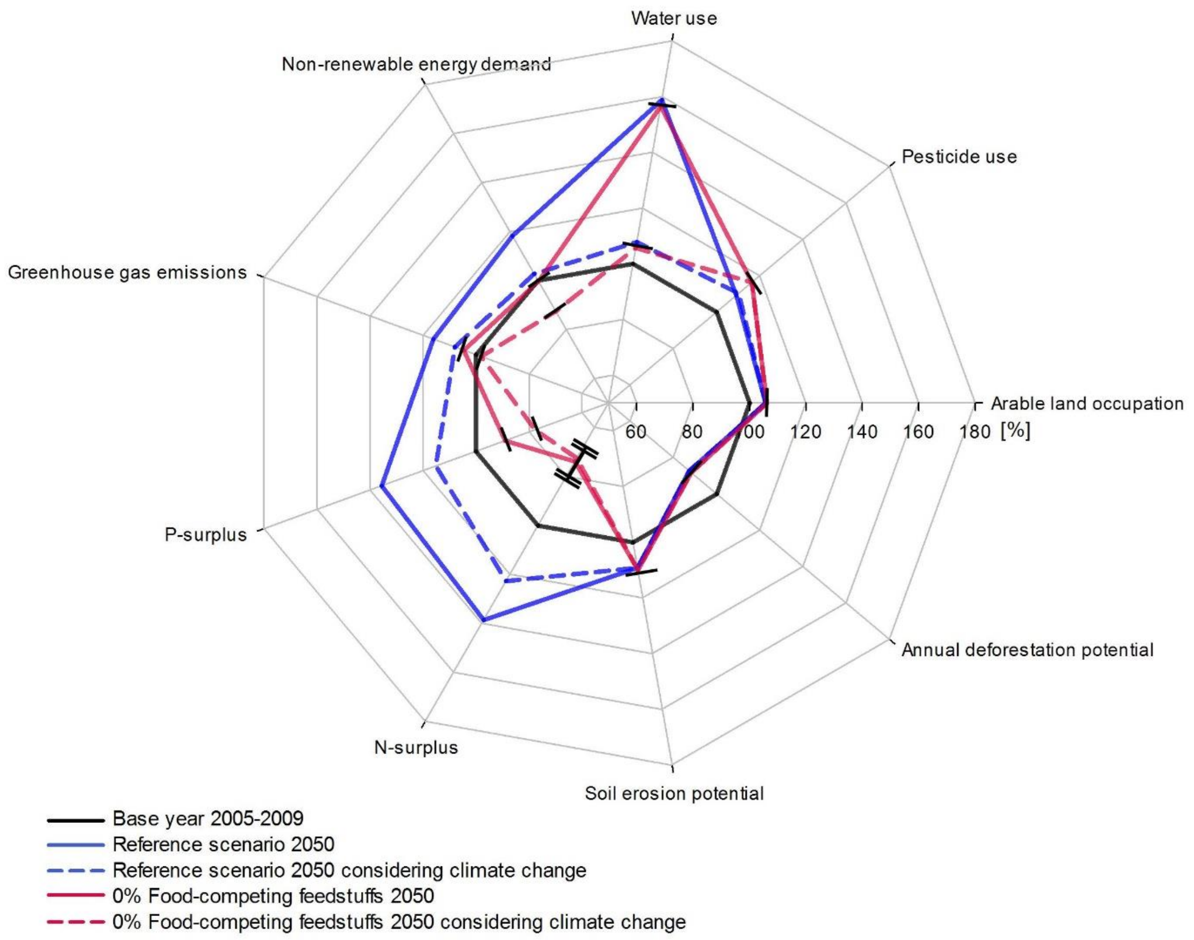

Figure S14 Change of environmental pressures resulting from a reduction in food-competing feedstuffs relative to the base year [%] assuming equal land total land occupation in the reference scenario and the scenario with 0% food-competing feedstuffs. Solid lines: no negative impact of climate change on yields assumed; dashed lines: negative impact of climate change on yields assumed; black: base year; blue: reference scenario (same level of food-competing feedstuffs assumed for 2050); red: 0% food-competing feedstuffs. Black whiskers represent the range resulting from different assumptions on animal yield reduction (0%-40%) for constant land use in all scenarios

Table S34 Overview of the main model outputs for all scenarios and sensitivity analyses with constant land use not considering climate change impacts on yields (absolute values). FCF: Food-competing feedstuffs.

Indicators	Unit	Base year 2005-2009	Reference Scenario 2050	0% livestock yield reduction due reducting FCF					20% livestock yield reduction due reducting FCF					40% livestock yield reduction due reducting FCF				
				80% FCF	60% FCF	40% FCF	20% FCF	0% FCF	80% FCF	60% FCF	40% FCF	20% FCF	0% FCF	80% FCF	60% FCF	40% FCF	20% FCF	0% FCF
Human population	billion people	6.67	9.14	9.14	9.14	9.14	9.14	9.14	9.14	9.14	9.14	9.14	9.14	9.14	9.14	9.14	9.14	9.14
Land occupation: Total	billion hectares	4.92	5.01	5.01	5.01	5.01	5.01	5.01	5.01	5.01	5.01	5.01	5.01	5.01	5.01	5.01	5.01	5.01
Land occupation: Crop	billion hectares	1.54	1.63	1.63	1.63	1.63	1.63	1.63	1.63	1.63	1.63	1.63	1.63	1.63	1.63	1.63	1.63	1.63
Land occupation: Grass	billion hectares	3.38	3.38	3.38	3.38	3.38	3.38	3.38	3.38	3.38	3.38	3.38	3.38	3.38	3.38	3.38	3.38	3.38
Number of cattle	billion animals	1.39	1.85	1.74	1.66	1.58	1.51	1.45	1.74	1.66	1.58	1.51	1.45	1.74	1.66	1.58	1.51	1.45
Number of pigs	billion animals	0.92	1.17	0.97	0.76	0.55	0.35	0.14	0.97	0.76	0.55	0.34	0.14	0.97	0.76	0.55	0.34	0.14
Number of chickens	billion animals	17.56	33.85	28.76	23.07	17.39	11.74	6.12	28.72	22.99	17.27	11.59	5.95	28.67	22.91	17.17	11.47	5.81
Number of buffaloes	billion animals	0.18	0.27	0.26	0.26	0.26	0.26	0.26	0.26	0.26	0.26	0.26	0.26	0.26	0.26	0.26	0.26	0.26
Number of goats	billion animals	0.86	1.39	1.33	1.29	1.25	1.22	1.18	1.33	1.29	1.25	1.22	1.18	1.33	1.29	1.25	1.22	1.18
Number of sheep	billion animals	1.10	1.60	1.52	1.47	1.43	1.38	1.34	1.52	1.47	1.43	1.38	1.34	1.52	1.47	1.43	1.38	1.34
Energy intake per person and day	kcal/cap/day	2,763.27	3,028.06	3,304.16	3,572.31	3,832.52	4,087.03	4,349.39	3,268.54	3,510.86	3,754.24	4,000.17	4,261.57	3,234.40	3,454.61	3,686.12	3,928.64	4,193.52
Protein intake per person and day	g Protein/cap/day	76.83	82.48	86.10	90.19	94.69	99.92	106.82	84.04	86.63	90.15	94.88	101.72	82.07	83.38	86.20	90.73	97.76
Protein-Calorie ratio	Ratio	0.11	0.11	0.10	0.10	0.10	0.10	0.10	0.10	0.10	0.10	0.09	0.10	0.10	0.10	0.09	0.09	0.09
Calorie share of livestock products	%	15%	17%	14%	11%	9%	7%	6%	13%	10%	7%	5%	4%	12%	8%	5%	3%	2%
Protein share of livestock products	%	34%	38%	32%	27%	22%	18%	14%	30%	24%	18%	13%	9%	28%	20%	14%	9%	5%
Calorie share of meat	%	8%	9%	7%	6%	4%	3%	2%	7%	5%	3%	2%	1%	6%	4%	3%	1%	1%
Protein share of meat	%	20%	23%	19%	15%	12%	9%	6%	18%	14%	10%	7%	4%	17%	12%	8%	5%	2%
Calorie share of milk	%	6%	6%	6%	5%	4%	4%	4%	5%	4%	3%	3%	2%	5%	4%	3%	2%	1%
Protein share of milk	%	11%	11%	10%	9%	8%	8%	7%	10%	8%	7%	6%	5%	9%	7%	5%	4%	3%
Meat quantity per capita and day	g/cap/day	110.46	135.73	117.61	99.20	80.94	62.86	44.92	108.32	83.84	62.53	44.19	28.60	99.42	69.80	46.54	28.87	16.01
Milk quantity per capita and day	g/cap/day	241.63	274.17	257.67	246.33	236.29	227.31	219.22	237.47	208.50	182.98	160.39	140.30	218.09	173.81	136.48	105.11	78.92
N-surplus	million tonnes N	87.91	121.76	110.84	99.62	89.00	77.65	62.22	112.38	102.28	92.30	81.07	64.90	113.93	104.94	95.63	84.53	67.56
N-surplus per hectare	kg N/ha	18.57	24.98	22.73	20.42	18.24	15.91	12.75	23.05	20.97	18.92	16.61	13.29	23.36	21.51	19.60	17.32	13.84
P-surplus	million tonnes P	47.18	63.95	59.62	55.31	51.17	46.78	41.80	59.79	55.60	51.53	47.13	41.99	59.97	55.90	51.88	47.49	42.18
P-surplus per hectare	kg P/ha	9.97	13.12	12.23	11.34	10.49	9.58	8.56	12.26	11.40	10.56	9.66	8.60	12.30	11.46	10.63	9.73	8.64
GHG emissions	Gt CO_2-eq	11.01	12.78	12.54	12.31	12.08	11.83	11.52	12.54	12.31	12.07	11.83	11.52	12.54	12.31	12.07	11.83	11.52
Non-renewable energy demand	Exajoules	22.58	26.69	26.11	25.41	24.68	23.80	22.53	26.10	25.41	24.67	23.79	22.52	26.10	25.40	24.66	23.78	22.51
Pesticide use	dimensionless	14.07	15.36	15.64	15.79	15.93	16.10	16.37	15.64	15.79	15.93	16.10	16.37	15.64	15.79	15.93	16.10	16.37
Freshwater use	km^3	1,371.32	2,177.70	2,190.55	2,191.26	2,182.84	2,169.14	2,146.32	2,190.55	2,191.25	2,182.82	2,169.11	2,146.30	2,190.54	2,191.23	2,182.81	2,169.09	2,146.28
Deforestation	million ha	8.23	7.18	7.20	7.21	7.23	7.24	7.26	7.20	7.21	7.23	7.24	7.26	7.20	7.21	7.23	7.24	7.26
Soil erosion due to water	billion tonnes	33.71	36.77	36.83	36.90	36.95	36.99	37.05	36.83	36.90	36.95	36.99	37.05	36.83	36.90	36.95	36.99	37.05

Table S35 Overview of the main model outputs for all scenarios and sensitivity analyses with constant land use not considering climate change impacts on yields (relative values compared to base year). FCF: Food-competing feedstuffs.

Indicators	Unit	Base year 2005-2009	Reference Scenario 2050	0% livestock yield reduction due reducting FCF					20% livestock yield reduction due reducting FCF					40% livestock yield reduction due reducting FCF				
				80% FCF	60% FCF	40% FCF	20% FCF	0% FCF	80% FCF	60% FCF	40% FCF	20% FCF	0% FCF	80% FCF	60% FCF	40% FCF	20% FCF	0% FCF
Human population	billion people	6.67	137%	137%	137%	137%	137%	137%	137%	137%	137%	137%	137%	137%	137%	137%	137%	137%
Land occupation: Total	billion hectares	4.92	102%	102%	102%	102%	102%	102%	102%	102%	102%	102%	102%	102%	102%	102%	102%	102%
Land occupation: Crop	billion hectares	1.54	106%	106%	106%	106%	106%	106%	106%	106%	106%	106%	106%	106%	106%	106%	106%	106%
Land occupation: Grass	billion hectares	3.38	100%	100%	100%	100%	100%	100%	100%	100%	100%	100%	100%	100%	100%	100%	100%	100%
Number of cattle	billion animals	1.39	133%	125%	119%	114%	109%	104%	125%	119%	114%	109%	104%	125%	119%	114%	109%	104%
Number of pigs	billion animals	0.92	127%	106%	83%	60%	38%	15%	105%	83%	60%	37%	15%	105%	83%	60%	37%	15%
Number of chickens	billion animals	17.56	193%	164%	131%	99%	67%	35%	164%	131%	98%	66%	34%	163%	131%	98%	65%	33%
Number of buffaloes	billion animals	0.18	146%	144%	144%	144%	144%	144%	144%	144%	144%	144%	144%	144%	144%	144%	144%	144%
Number of goats	billion animals	0.86	161%	155%	150%	145%	141%	137%	155%	150%	145%	141%	137%	155%	150%	145%	141%	137%
Number of sheep	billion animals	1.10	146%	139%	134%	130%	126%	122%	139%	134%	130%	126%	122%	139%	134%	130%	126%	122%
Energy intake per person and day	kcal/cap/day	2,763.27	110%	120%	129%	139%	148%	157%	118%	127%	136%	145%	154%	117%	125%	133%	142%	152%
Protein intake per person and day	g Protein/cap/day	76.83	107%	112%	117%	123%	130%	139%	109%	113%	117%	123%	132%	107%	109%	112%	118%	127%
Protein-Calorie ratio	Ratio	0.11	98%	94%	91%	89%	88%	88%	92%	89%	86%	85%	86%	91%	87%	84%	83%	84%
Calorie share of livestock products	%	15%	111%	90%	73%	59%	47%	37%	84%	63%	47%	34%	24%	78%	53%	35%	23%	14%
Protein share of livestock products	%	34%	111%	94%	78%	64%	52%	40%	89%	69%	52%	38%	27%	83%	60%	41%	26%	16%
Calorie share of meat	%	8%	110%	87%	67%	50%	35%	22%	81%	57%	39%	25%	15%	75%	49%	30%	17%	8%
Protein share of meat	%	20%	116%	96%	78%	61%	46%	32%	91%	69%	50%	34%	21%	85%	59%	39%	23%	12%
Calorie share of milk	%	6%	114%	98%	87%	78%	70%	63%	92%	75%	61%	50%	41%	85%	63%	47%	34%	24%
Protein share of milk	%	11%	104%	93%	84%	76%	69%	62%	88%	74%	62%	52%	42%	82%	64%	49%	35%	25%
Meat quantity per capita and day	g/cap/day	110.46	123%	106%	90%	73%	57%	41%	98%	76%	57%	40%	26%	90%	63%	42%	26%	14%
Milk quantity per capita and day	g/cap/day	241.63	113%	107%	102%	98%	94%	91%	98%	86%	76%	66%	58%	90%	72%	56%	43%	33%
N-surplus	million tonnes N	87.91	139%	126%	113%	101%	88%	71%	128%	116%	105%	92%	74%	130%	119%	109%	96%	77%
N-surplus per hectare	kg N/ha	18.57	134%	122%	110%	98%	86%	69%	124%	113%	102%	89%	72%	126%	116%	106%	93%	75%
P-surplus	million tonnes P	47.18	136%	126%	117%	108%	99%	89%	127%	118%	109%	100%	89%	127%	118%	110%	101%	89%
P-surplus per hectare	kg P/ha	9.97	132%	123%	114%	105%	96%	86%	123%	114%	106%	97%	86%	123%	115%	107%	98%	87%
GHG emissions	Gt CO_2-eq	11.01	116%	114%	112%	110%	107%	105%	114%	112%	110%	107%	105%	114%	112%	110%	107%	105%
Non-renewable energy demand	Exajoules	22.58	118%	116%	113%	109%	105%	100%	116%	113%	109%	105%	100%	116%	112%	109%	105%	100%
Pesticide use	dimensionless	14.07	109%	111%	112%	113%	114%	116%	111%	112%	113%	114%	116%	111%	112%	113%	114%	116%
Freshwater use	km^3	1,371.32	159%	160%	160%	159%	158%	157%	160%	160%	159%	158%	157%	160%	160%	159%	158%	157%
Deforestation	million ha	8.23	87%	88%	88%	88%	88%	88%	88%	88%	88%	88%	88%	88%	88%	88%	88%	88%
Soil erosion due to water	billion tonnes	33.71	109%	109%	109%	110%	110%	110%	109%	109%	110%	110%	110%	109%	109%	110%	110%	110%

Table S36 Overview of the main model outputs for all scenarios and sensitivity analyses with constant land use not considering climate change impacts on yields (relative values compared to reference scenario). FCF: Food-competing feedstuffs.

Indicators	Unit	Base year 2005-2009	Reference Scenario 2050	0% livestock yield reduction due reducting FCF					20% livestock yield reduction due reducting FCF					40% livestock yield reduction due reducting FCF				
				80% FCF	60% FCF	40% FCF	20% FCF	0% FCF	80% FCF	60% FCF	40% FCF	20% FCF	0% FCF	80% FCF	60% FCF	40% FCF	20% FCF	0% FCF
Human population	billion people	6.67	9.14	100%	100%	100%	100%	100%	100%	100%	100%	100%	100%	100%	100%	100%	100%	100%
Land occupation: Total	billion hectares	4.92	5.01	100%	100%	100%	100%	100%	100%	100%	100%	100%	100%	100%	100%	100%	100%	100%
Land occupation: Crop	billion hectares	1.54	1.63	100%	100%	100%	100%	100%	100%	100%	100%	100%	100%	100%	100%	100%	100%	100%
Land occupation: Grass	billion hectares	3.38	3.38	100%	100%	100%	100%	100%	100%	100%	100%	100%	100%	100%	100%	100%	100%	100%
Number of cattle	billion animals	1.39	1.85	94%	90%	86%	82%	79%	94%	90%	86%	82%	79%	94%	90%	86%	82%	79%
Number of pigs	billion animals	0.92	1.17	83%	65%	47%	30%	12%	83%	65%	47%	29%	12%	83%	65%	47%	29%	12%
Number of chickens	billion animals	17.56	33.85	85%	68%	51%	35%	18%	85%	68%	51%	34%	18%	85%	68%	51%	34%	17%
Number of buffaloes	billion animals	0.18	0.27	98%	98%	98%	98%	98%	98%	98%	98%	98%	98%	98%	98%	98%	98%	98%
Number of goats	billion animals	0.86	1.39	96%	93%	90%	87%	85%	96%	93%	90%	87%	85%	96%	93%	90%	87%	85%
Number of sheep	billion animals	1.10	1.60	95%	92%	89%	87%	84%	95%	92%	89%	87%	84%	95%	92%	89%	87%	84%
Energy intake per person and day	kcal/cap/day	2,763.27	3,028.06	109%	118%	127%	135%	144%	108%	116%	124%	132%	141%	107%	114%	122%	130%	138%
Protein intake per person and day	g Protein/cap/day	76.83	82.48	104%	109%	115%	121%	130%	102%	105%	109%	115%	123%	100%	101%	105%	110%	119%
Protein-Calorie ratio	Ratio	0.11	0.11	96%	93%	91%	90%	90%	94%	91%	88%	87%	88%	93%	89%	86%	85%	86%
Calorie share of livestock products	%	0.15	0.17	81%	66%	53%	43%	33%	76%	57%	42%	31%	22%	70%	48%	32%	20%	12%
Protein share of livestock products	%	0.34	0.38	85%	71%	58%	47%	36%	80%	62%	47%	35%	24%	75%	54%	37%	24%	14%
Calorie share of meat	%	0.08	0.09	79%	61%	45%	32%	20%	73%	52%	36%	23%	13%	68%	44%	27%	15%	8%
Protein share of meat	%	0.20	0.23	83%	67%	53%	40%	28%	78%	59%	43%	29%	18%	74%	51%	33%	20%	11%
Calorie share of milk	%	0.06	0.06	86%	76%	68%	61%	55%	80%	65%	54%	44%	36%	74%	55%	41%	29%	21%
Protein share of milk	%	0.11	0.11	89%	81%	74%	67%	60%	84%	72%	60%	50%	40%	80%	62%	47%	34%	24%
Meat quantity per capita and day	g/cap/day	110.46	135.73	87%	73%	60%	46%	33%	80%	62%	46%	33%	21%	73%	51%	34%	21%	12%
Milk quantity per capita and day	g/cap/day	241.63	274.17	94%	90%	86%	83%	80%	87%	76%	67%	58%	51%	80%	63%	50%	38%	29%
N-surplus	million tonnes N	87.91	121.76	91%	82%	73%	64%	51%	92%	84%	76%	67%	53%	94%	86%	79%	69%	55%
N-surplus per hectare	kg N/ha	18.57	24.98	91%	82%	73%	64%	51%	92%	84%	76%	67%	53%	94%	86%	78%	69%	55%
P-surplus	million tonnes P	47.18	63.95	93%	86%	80%	73%	65%	94%	87%	81%	74%	66%	94%	87%	81%	74%	66%
P-surplus per hectare	kg P/ha	9.97	13.12	93%	86%	80%	73%	65%	93%	87%	81%	74%	66%	94%	87%	81%	74%	66%
GHG emissions	Gt CO_2-eq	11.01	12.78	98%	96%	94%	93%	90%	98%	96%	94%	93%	90%	98%	96%	94%	92%	90%
Non-renewable energy demand	Exajoules	22.58	26.69	98%	95%	92%	89%	84%	98%	95%	92%	89%	84%	98%	95%	92%	89%	84%
Pesticide use	dimensionless	14.07	15.36	102%	103%	104%	105%	107%	102%	103%	104%	105%	107%	102%	103%	104%	105%	107%
Freshwater use	km^3	1,371.32	2,177.70	101%	101%	100%	100%	99%	101%	101%	100%	100%	99%	101%	101%	100%	100%	99%
Deforestation	million ha	8.23	7.18	100%	100%	101%	101%	101%	100%	100%	101%	101%	101%	100%	100%	101%	101%	101%
Soil erosion due to water	billion tonnes	33.71	36.77	100%	100%	100%	101%	101%	100%	100%	100%	101%	101%	100%	100%	100%	101%	101%

Table S37 Overview of the main model outputs for all scenarios and sensitivity analyses with constant land use considering climate change impacts on yields (absolute values). FCF: Food-competing feedstuffs.

Indicators	Unit	Base year 2005-2009	Reference Scenario 2050	0% livestock yield reduction due reducting FCF					20% livestock yield reduction due reducting FCF					40% livestock yield reduction due reducting FCF				
				80% FCF	60% FCF	40% FCF	20% FCF	0% FCF	80% FCF	60% FCF	40% FCF	20% FCF	0% FCF	80% FCF	60% FCF	40% FCF	20% FCF	0% FCF
Human population	billion people	6.67	9.14	9.14	9.14	9.14	9.14	9.14	9.14	9.14	9.14	9.14	9.14	9.14	9.14	9.14	9.14	9.14
Land occupation: Total	billion hectares	4.92	5.01	5.01	5.01	5.01	5.01	5.01	5.01	5.01	5.01	5.01	5.01	5.01	5.01	5.01	5.01	5.01
Land occupation: Crop	billion hectares	1.54	1.63	1.63	1.63	1.63	1.63	1.63	1.63	1.63	1.63	1.63	1.63	1.63	1.63	1.63	1.63	1.63
Land occupation: Grass	billion hectares	3.38	3.38	3.38	3.38	3.38	3.38	3.38	3.38	3.38	3.38	3.38	3.38	3.38	3.38	3.38	3.38	3.38
Number of cattle	billion animals	1.39	1.78	1.68	1.60	1.53	1.46	1.40	1.68	1.60	1.53	1.46	1.40	1.68	1.60	1.53	1.46	1.40
Number of pigs	billion animals	0.92	0.90	0.74	0.58	0.42	0.26	0.10	0.74	0.58	0.42	0.26	0.10	0.74	0.58	0.42	0.26	0.10
Number of chickens	billion animals	17.56	25.31	21.09	16.87	12.66	8.49	4.39	21.05	16.78	12.54	8.35	4.22	21.01	16.71	12.44	8.23	4.09
Number of buffaloes	billion animals	0.18	0.26	0.26	0.26	0.26	0.26	0.26	0.26	0.26	0.26	0.26	0.26	0.26	0.26	0.26	0.26	0.26
Number of goats	billion animals	0.86	1.33	1.29	1.25	1.21	1.18	1.14	1.29	1.25	1.21	1.18	1.14	1.29	1.25	1.21	1.18	1.14
Number of sheep	billion animals	1.10	1.52	1.47	1.42	1.38	1.34	1.30	1.47	1.42	1.38	1.34	1.30	1.47	1.42	1.38	1.34	1.30
Energy intake per person and day	kcal/cap/day	2,763.27	2,171.56	2,373.11	2,567.33	2,754.38	2,935.59	3,120.99	2,342.06	2,513.22	2,684.54	2,856.63	3,038.97	2,312.29	2,463.71	2,623.76	2,791.61	2,975.40
Protein intake per person and day	g Protein/cap/day	76.83	62.34	64.73	67.35	70.21	73.52	77.88	62.94	64.24	66.18	68.96	73.13	61.23	61.38	62.67	65.20	69.45
Protein-Calorie ratio	Ratio	0.11	0.11	0.11	0.10	0.10	0.10	0.10	0.11	0.10	0.10	0.10	0.10	0.11	0.10	0.10	0.09	0.09
Calorie share of livestock products	%	15%	21%	17%	14%	11%	9%	7%	16%	12%	9%	7%	5%	15%	10%	7%	4%	3%
Protein share of livestock products	%	34%	44%	37%	32%	27%	22%	17%	36%	28%	22%	16%	12%	34%	25%	17%	11%	7%
Calorie share of meat	%	8%	11%	8%	6%	5%	4%	2%	8%	6%	4%	3%	2%	7%	5%	3%	2%	1%
Protein share of meat	%	20%	26%	21%	18%	14%	11%	8%	20%	16%	12%	8%	5%	19%	14%	9%	6%	3%
Calorie share of milk	%	6%	9%	7%	7%	6%	5%	5%	7%	6%	5%	4%	3%	7%	5%	4%	3%	2%
Protein share of milk	%	11%	15%	13%	12%	11%	10%	9%	13%	11%	9%	8%	6%	12%	9%	7%	5%	4%
Meat quantity per capita and day	g/cap/day	110.46	111.27	96.71	82.29	68.04	54.04	40.24	89.06	69.54	52.56	37.98	25.62	81.74	57.89	39.11	24.80	14.34
Milk quantity per capita and day	g/cap/day	241.63	261.46	248.96	238.01	228.30	219.62	211.81	229.44	201.45	176.80	154.97	135.56	210.72	167.94	131.87	101.55	76.25
N-surplus	million tonnes N	87.91	107.82	97.40	88.60	80.39	71.92	61.22	98.72	90.87	83.24	74.90	63.72	100.05	93.16	86.11	77.92	66.18
N-surplus per hectare	kg N/ha	18.57	22.12	19.97	18.16	16.48	14.74	12.54	20.25	18.63	17.06	15.35	13.05	20.52	19.10	17.65	15.97	13.56
P-surplus	million tonnes P	47.18	54.36	50.49	46.93	43.53	40.06	36.50	50.65	47.19	43.84	40.37	36.69	50.81	47.45	44.15	40.68	36.88
P-surplus per hectare	kg P/ha	9.97	11.15	10.35	9.62	8.92	8.21	7.48	10.39	9.67	8.98	8.27	7.52	10.42	9.73	9.05	8.34	7.55
GHG emissions	Gt CO_2-eq	11.01	11.89	11.67	11.47	11.27	11.06	10.81	11.67	11.47	11.27	11.06	10.81	11.67	11.47	11.27	11.06	10.81
Non-renewable energy demand	Exajoules	22.58	23.21	22.62	22.05	21.44	20.73	19.75	22.62	22.04	21.44	20.72	19.74	22.61	22.04	21.43	20.71	19.73
Pesticide use	dimensionless	14.07	15.51	15.64	15.79	15.93	16.10	16.37	15.64	15.79	15.93	16.10	16.37	15.64	15.79	15.93	16.10	16.37
Freshwater use	km^3	1,371.32	1,478.51	1,483.38	1,483.95	1,477.83	1,466.96	1,450.19	1,483.38	1,483.93	1,477.81	1,466.94	1,450.16	1,483.37	1,483.92	1,477.79	1,466.92	1,450.14
Deforestation	million ha	8.23	7.18	7.20	7.21	7.23	7.24	7.26	7.20	7.21	7.23	7.24	7.26	7.20	7.21	7.23	7.24	7.26
Soil erosion due to water	billion tonnes	33.71	36.77	36.83	36.90	36.95	36.99	37.05	36.83	36.90	36.95	36.99	37.05	36.83	36.90	36.95	36.99	37.05

Table S38 Overview of the main model outputs for all scenarios and sensitivity analyses with constant land use considering climate change impacts on yields (relative values compared to base year). FCF: Food-competing feedstuffs.

Indicators	Unit	Base year 2005-2009	Reference Scenario 2050	0% livestock yield reduction due reducting FCF					20% livestock yield reduction due reducting FCF					40% livestock yield reduction due reducting FCF				
				80% FCF	60% FCF	40% FCF	20% FCF	0% FCF	80% FCF	60% FCF	40% FCF	20% FCF	0% FCF	80% FCF	60% FCF	40% FCF	20% FCF	0% FCF
Human population	billion people	6.67	137%	137%	137%	137%	137%	137%	137%	137%	137%	137%	137%	137%	137%	137%	137%	137%
Land occupation: Total	billion hectares	4.92	102%	102%	102%	102%	102%	102%	102%	102%	102%	102%	102%	102%	102%	102%	102%	102%
Land occupation: Crop	billion hectares	1.54	106%	106%	106%	106%	106%	106%	106%	106%	106%	106%	106%	106%	106%	106%	106%	106%
Land occupation: Grass	billion hectares	3.38	100%	100%	100%	100%	100%	100%	100%	100%	100%	100%	100%	100%	100%	100%	100%	100%
Number of cattle	billion animals	1.39	128%	121%	115%	110%	105%	101%	121%	115%	110%	105%	101%	121%	115%	110%	105%	101%
Number of pigs	billion animals	0.92	98%	81%	63%	46%	28%	11%	81%	63%	45%	28%	11%	81%	63%	45%	28%	11%
Number of chickens	billion animals	17.56	144%	120%	96%	72%	48%	25%	120%	96%	71%	48%	24%	120%	95%	71%	47%	23%
Number of buffaloes	billion animals	0.18	139%	139%	139%	139%	139%	139%	139%	139%	139%	139%	139%	139%	139%	139%	139%	139%
Number of goats	billion animals	0.86	155%	150%	145%	141%	136%	133%	150%	145%	141%	136%	133%	150%	145%	141%	136%	133%
Number of sheep	billion animals	1.10	139%	134%	130%	126%	122%	118%	134%	130%	126%	122%	118%	134%	130%	126%	122%	118%
Energy intake per person and day	kcal/cap/day	2,763.27	79%	86%	93%	100%	106%	113%	85%	91%	97%	103%	110%	84%	89%	95%	101%	108%
Protein intake per person and day	g Protein/cap/day	76.83	81%	84%	88%	91%	96%	101%	82%	84%	86%	90%	95%	80%	80%	82%	85%	90%
Protein-Calorie ratio	Ratio	0.11	103%	98%	94%	92%	90%	90%	97%	92%	89%	87%	87%	95%	90%	86%	84%	84%
Calorie share of livestock products	%	15%	134%	110%	90%	74%	60%	48%	102%	78%	58%	43%	31%	95%	66%	45%	29%	18%
Protein share of livestock products	%	34%	128%	110%	93%	78%	64%	51%	104%	83%	64%	48%	35%	99%	73%	51%	34%	21%
Calorie share of meat	%	8%	126%	99%	77%	58%	42%	28%	92%	66%	46%	30%	18%	86%	56%	35%	20%	10%
Protein share of meat	%	20%	130%	108%	89%	71%	55%	40%	103%	79%	59%	41%	27%	97%	69%	46%	29%	16%
Calorie share of milk	%	6%	153%	133%	117%	105%	94%	86%	124%	101%	83%	68%	56%	115%	86%	63%	46%	32%
Protein share of milk	%	11%	133%	121%	110%	101%	92%	83%	115%	98%	83%	69%	57%	109%	86%	66%	48%	34%
Meat quantity per capita and day	g/cap/day	110.46	101%	88%	74%	62%	49%	36%	81%	63%	48%	34%	23%	74%	52%	35%	22%	13%
Milk quantity per capita and day	g/cap/day	241.63	108%	103%	98%	94%	91%	88%	95%	83%	73%	64%	56%	87%	70%	55%	42%	32%
N-surplus	million tonnes N	87.91	123%	111%	101%	91%	82%	70%	112%	103%	95%	85%	72%	114%	106%	98%	89%	75%
N-surplus per hectare	kg N/ha	18.57	119%	108%	98%	89%	79%	68%	109%	100%	92%	83%	70%	110%	103%	95%	86%	73%
P-surplus	million tonnes P	47.18	115%	107%	99%	92%	85%	77%	107%	100%	93%	86%	78%	108%	101%	94%	86%	78%
P-surplus per hectare	kg P/ha	9.97	112%	104%	97%	89%	82%	75%	104%	97%	90%	83%	75%	105%	98%	91%	84%	76%
GHG emissions	Gt CO_2-eq	11.01	108%	106%	104%	102%	100%	98%	106%	104%	102%	100%	98%	106%	104%	102%	100%	98%
Non-renewable energy demand	Exajoules	22.58	103%	100%	98%	95%	92%	87%	100%	98%	95%	92%	87%	100%	98%	95%	92%	87%
Pesticide use	dimensionless	14.07	110%	111%	112%	113%	114%	116%	111%	112%	113%	114%	116%	111%	112%	113%	114%	116%
Freshwater use	km^3	1,371.32	108%	108%	108%	108%	107%	106%	108%	108%	108%	107%	106%	108%	108%	108%	107%	106%
Deforestation	million ha	8.23	87%	88%	88%	88%	88%	88%	88%	88%	88%	88%	88%	88%	88%	88%	88%	88%
Soil erosion due to water	billion tonnes	33.71	109%	109%	109%	110%	110%	110%	109%	109%	110%	110%	110%	109%	109%	110%	110%	110%

Table S39 Overview of the main model outputs for all scenarios and sensitivity analyses with constant land use considering climate change impacts on yields (relative values compared to reference scenario). FCF: Food-competing feedstuffs.

Indicators	Unit	Base year 2005-2009	Reference Scenario 2050	0% livestock yield reduction due reducting FCF					20% livestock yield reduction due reducting FCF					40% livestock yield reduction due reducting FCF				
				80% FCF	60% FCF	40% FCF	20% FCF	0% FCF	80% FCF	60% FCF	40% FCF	20% FCF	0% FCF	80% FCF	60% FCF	40% FCF	20% FCF	0% FCF
Human population	billion people	6.67	9.14	100%	100%	100%	100%	100%	100%	100%	100%	100%	100%	100%	100%	100%	100%	100%
Land occupation: Total	billion hectares	4.92	5.01	100%	100%	100%	100%	100%	100%	100%	100%	100%	100%	100%	100%	100%	100%	100%
Land occupation: Crop	billion hectares	1.54	1.63	100%	100%	100%	100%	100%	100%	100%	100%	100%	100%	100%	100%	100%	100%	100%
Land occupation: Grass	billion hectares	3.38	3.38	100%	100%	100%	100%	100%	100%	100%	100%	100%	100%	100%	100%	100%	100%	100%
Number of cattle	billion animals	1.39	1.78	95%	90%	86%	82%	79%	95%	90%	86%	82%	79%	95%	90%	86%	82%	79%
Number of pigs	billion animals	0.92	0.90	82%	64%	46%	29%	11%	82%	64%	46%	29%	11%	82%	64%	46%	28%	11%
Number of chickens	billion animals	17.56	25.31	83%	67%	50%	34%	17%	83%	66%	50%	33%	17%	83%	66%	49%	33%	16%
Number of buffaloes	billion animals	0.18	0.26	100%	100%	100%	100%	100%	100%	100%	100%	100%	100%	100%	100%	100%	100%	100%
Number of goats	billion animals	0.86	1.33	97%	94%	91%	88%	86%	97%	94%	91%	88%	86%	97%	94%	91%	88%	86%
Number of sheep	billion animals	1.10	1.52	97%	93%	91%	88%	85%	97%	93%	91%	88%	85%	97%	93%	91%	88%	85%
Energy intake per person and day	kcal/cap/day	2,763.27	2,171.56	109%	118%	127%	135%	144%	108%	116%	124%	132%	140%	106%	113%	121%	129%	137%
Protein intake per person and day	g Protein/cap/day	76.83	62.34	104%	108%	113%	118%	125%	101%	103%	106%	111%	117%	98%	98%	101%	105%	111%
Protein-Calorie ratio	Ratio	0.11	0.11	95%	91%	89%	87%	87%	94%	89%	86%	84%	84%	92%	87%	83%	81%	81%
Calorie share of livestock products	%	0.15	0.21	82%	67%	55%	45%	36%	76%	58%	44%	32%	23%	71%	49%	33%	22%	13%
Protein share of livestock products	%	0.34	0.44	86%	73%	61%	50%	40%	81%	65%	50%	38%	27%	77%	57%	40%	26%	16%
Calorie share of meat	%	0.08	0.11	79%	61%	46%	33%	22%	73%	53%	36%	24%	14%	68%	45%	28%	16%	8%
Protein share of meat	%	0.20	0.26	84%	69%	55%	42%	31%	79%	61%	45%	32%	21%	75%	53%	36%	22%	12%
Calorie share of milk	%	0.06	0.09	87%	77%	68%	62%	56%	81%	66%	54%	45%	37%	76%	56%	42%	30%	21%
Protein share of milk	%	0.11	0.15	91%	83%	76%	69%	62%	86%	74%	62%	52%	43%	82%	65%	49%	36%	25%
Meat quantity per capita and day	g/cap/day	110.46	111.27	87%	74%	61%	49%	36%	80%	63%	47%	34%	23%	73%	52%	35%	22%	13%
Milk quantity per capita and day	g/cap/day	241.63	261.46	95%	91%	87%	84%	81%	88%	77%	68%	59%	52%	81%	64%	50%	39%	29%
N-surplus	million tonnes N	87.91	107.82	90%	82%	75%	67%	57%	92%	84%	77%	69%	59%	93%	86%	80%	72%	61%
N-surplus per hectare	kg N/ha	18.57	22.12	90%	82%	74%	67%	57%	92%	84%	77%	69%	59%	93%	86%	80%	72%	61%
P-surplus	million tonnes P	47.18	54.36	93%	86%	80%	74%	67%	93%	87%	81%	74%	67%	93%	87%	81%	75%	68%
P-surplus per hectare	kg P/ha	9.97	11.15	93%	86%	80%	74%	67%	93%	87%	81%	74%	67%	93%	87%	81%	75%	68%
GHG emissions	Gt CO_2-eq	11.01	11.89	98%	96%	95%	93%	91%	98%	96%	95%	93%	91%	98%	96%	95%	93%	91%
Non-renewable energy demand	Exajoules	22.58	23.21	97%	95%	92%	89%	85%	97%	95%	92%	89%	85%	97%	95%	92%	89%	85%
Pesticide use	dimensionless	14.07	15.51	101%	102%	103%	104%	106%	101%	102%	103%	104%	106%	101%	102%	103%	104%	106%
Freshwater use	km^3	1,371.32	1,478.51	100%	100%	100%	99%	98%	100%	100%	100%	99%	98%	100%	100%	100%	99%	98%
Deforestation	million ha	8.23	7.18	100%	100%	101%	101%	101%	100%	100%	101%	101%	101%	100%	100%	101%	101%	101%
Soil erosion due to water	billion tonnes	33.71	36.77	100%	100%	100%	101%	101%	100%	100%	100%	101%	101%	100%	100%	100%	101%	101%

Appendices

Table S40 List of activities

Activity group	Activities
**Grains**	Barley, buckwheat, canary seed, fonio, maize, millet, oats, popcorn, quinoa, rice, rye, sorghum, triticale, wheat, other cereals
**Legumes**	Alfalfa for forage and silage, alfalfa for meal and pellets, bambara beans, beans (dry), broad beans, horse beans, chick peas, clover for forage and silage, Cow peas, groundnuts, lentils, lupins, peas, pigeon peas, soybeans, vetches, other pulses, other leguminous
**Starchy roots**	Cassava, potatoes, sweet potatoes, taro (cocoyam), yams, yautia (cocoyam), other roots and tubers
**Oil crops**	Castor oil seed, coconuts, hempseed, jojoba, kapok fruit, karite nuts (Sheanuts), kolanuts, linseed, melonseed, mustard seed, oil palm fruit, olives, poppy seed, rapeseed, safflower seed, sesame seed, sunflower seed, tallow tree, other oilseeds
**Sugars and sweeteners**	maple, sugar beet, sugar cane, other sugar crops
**Vegetables**	Artichokes, asparagus, beans (green), cabbages and other brassicas, carrots and turnips, cauliflowers and broccoli, chicory roots, chillies and peppers (dry), chillies and peppers (green), cucumbers and gherkins, eggplants (aubergines), garlic, leeks and other alliaceae vegetables, lettuce and chicory, maize (green), mushrooms and truffles, okra, onions (inc. shallots) (green), onions (dry), other melons (inc.cantaloupes), Peas (green), Pumpkins, squash and gourds, spinach, string beans, tomatoes, watermelons, other vegetables
**Fruits**	Apples, apricots, avocados, bananas, blueberries, carobs, cashewapple, cherries, Cranberries, Currants, Dates, Figs, Fruit Fresh Nes, Fruit, tropical fresh nes, Fruits, all, Fruits, most, Gooseberries, Grapefruit (inc. pomelos), Grapes, Kiwi fruit, Lemons and limes, Mangoes, mangosteens, guavas, oranges, papayas, peaches and nectarines, pears, persimmons, pineapples, plantains, plums and sloes, quinces, raspberries, sour cherries, strawberries, tangerines, mandarins, other fruits
**Tree nuts**	Almonds with shell, arecanuts, brazil nuts with shell, cashew nuts with shell, chestnuts, hazelnuts with shell, pistachios, tung nuts, walnuts with shell, other nuts
**Stimulants and spices**	Anise, badian, fennel, corian., cinnamon (canella), cloves, cocoa beans, coffee (green), ginger, hops, maté, nutmeg, mace and cardamoms, oil of citronella, pepper (Piper spp.), peppermint, pyrethrum (dried), tea, tobacco (unmanufactured), vanilla, other spices
**Fibers and Rubber**	Agave, arabic gum, coir, flax fibre and tow, natural gums, hemp tow waste, jute, manila fibre (Abaca), natural rubber, ramie, seed cotton, sisal, other fibers
**Fodder crops***	Alfalfa for forage and silage, alfalfa for meal and pellets, beets for fodder, clover for forage and silage, maize for forage and silage, swedes for fodder, triticale, turnips for fodder, other fodder crops, grassland
**Animals**	Asses, beehives, buffaloes, camels, cattle, cattle (including subcategories in herd structure models), chickens (including subcategories in herd structure models), ducks, game, geese and guinea fowls, goats, horses, mules, other camelids, other rodents, pigeons, other birds, pigs, Pigs (including subcategories in herd structure models), rabbits and hares, sheep, snails (not sea snails), turkeys, other animals
**Fish and seafood**	Aquatic plants, cephalopods, crustaceans, demersal fish, freshwater fish, aquatic mammals, pelagic fish, other fish and seafood

*overlapping with crop groups above

Table S41 Country-specific ratings of pesticide legislation (PL) and the accessibility of pesticides to farmers (AP)

Country	PL	AP
Afghanistan	3	1
Albania	2.5	2.5
Algeria	2	2
American Samoa	2	3
Andorra	2	3
Angola	3	1
Anguilla	2	3
Antigua and Barbuda	2	3
Argentina	2	2.5
Armenia	3	3
Aruba	2	3
Australia	1	3
Austria	1	3
Azerbaijan	3	2
Bahamas	2	3
Bahrain	2	2.5
Bangladesh	3	1
Barbados	2	3
Belarus	2.5	3
Belgium	1	3
Belize	2	3
Benin	2.5	1
Bermuda	2	3
Bhutan	3	1.5
Boli*via* (Plurinational State of)	3	2
Bosnia and Herzegovina	2	3
Botswana	2	1.5
Brazil	1.5	2.5
British Virgin Islands	2	3
Brunei Darussalam	2	2
Bulgaria	2.5	3
Burkina Faso	2.5	1
Burundi	2	2
Cambodia	3	1
Cameroon	3	2
Canada	1	3
Cape Verde	3	1.5
Cayman Islands	2	3
Central African Republic	3	1
Chad	3	1
Channel Islands	1	3
Chile	2	3
China	3	2
Colombia	2	2.5
Comoros	2	2
Congo	3	1
Cook Islands	1.5	3
Costa Rica	2	3
Côte d'Ivoire	3	1
Croatia	1.5	3
Cuba	2.5	2
Cyprus	2	3
Czech Republic	2	3
Democratic People's Republic of Korea	3	1.5
Democratic Republic of the Congo	3	1
Denmark	1	3
Djibouti	3	2
Dominica	2	2.5
Dominican Republic	2	2.5
Ecuador	2.5	2.5
Egypt	2	3
El Salvador	2	3
Equatorial Guinea	3	2
Eritrea	2.5	1
Estonia	1.5	3
Ethiopia	2	1.5
Falkland Islands (Malvinas)	2	3
Faroe Islands	1	3
Fiji	2	3
Finland	1	3
France	1	3
French Guiana	2	2
French Polynesia	1.5	3
Gabon	3	1.5
Gambia	2.5	1
Georgia	3	2
Germany	1	3
Ghana	2	1.5
Gibraltar	2	3
Greece	2	3
Greenland	1	2.5
Grenada	2	3
Guadeloupe	2	2.5
Guam	2	3
Guatemala	2	3
Guinea	2.5	1
Guinea-Bissau	3	1
Guyana	2.5	2
Haiti	3	2
Honduras	2	3
Hungary	2	3
Iceland	1	3
India	3	1.5
Indonesia	3	1
Iran (Islamic Republic of)	3	2
Iraq	2	2
Ireland	1	3
Isle of Man	1	3
Israel	2	3
Italy	1.5	3
Jamaica	2	2.5
Japan	1	3
Jordan	2	3
Kazakhstan	3	2
Kenya	2	2
Kiribati	2	3
Kuwait	2	3
Kyrgyzstan	3	2
Lao People's Democratic Republic	3	1
Lat*via*	1.5	3
Lebanon	2	2
Lesotho	2	2
Liberia	3	1
Libya	2	2
Liechtenstein	2	3
Lithuania	1.5	3
Luxembourg	1	3
Madagascar	2	2
Malawi	2	2
Malaysia	2	1.5
Maldives	2	2
Mali	2.5	1
Malta	1.5	3
Marshall Islands		
Martinique	2	3
Mauritania	2.5	1
Mauritius	2	2
Mayotte	3	1
Mexico	2	2.5
Micronesia (Federated States of)	2	3
Mongolia	3	1
Montenegro	2	3
Montserrat	2	3
Morocco	1	2
Mozambique	2	2
Myanmar	3	1
Namibia	2	2
Nauru	2	3
Nepal	3	1
Netherlands	1	3
Netherlands Antilles	2	3
New Caledonia	1.5	3
New Zealand	1	3
Nicaragua	2	2.5
Niger	2.5	1
Nigeria	3	1
Niue	2	2.5
Norfolk Island		
Northern Mariana Islands		
Norway	1	3
Occupied Palestinian Territory	3	1
Oman	2	3
Pakistan	3	1
Palau		
Panama	2	3
Papua New Guinea	2.5	1.5
Paraguay	2	3

Country	PL	AP
Peru	2.5	2
Philippines	2.5	1.5
Pitcairn Islands		
Poland	2	3
Portugal	1	3
Puerto Rico	2	2
Qatar	2	3
Republic of Korea	1.5	3
Republic of Moldova	3	2.5
Réunion	3	2
Romania	2	3
Russian Federation	3	2
Rwanda	2	2
Saint Helena	3	1
Saint Kitts and Nevis	2	3
Saint Lucia	2	3
Saint Pierre and Miquelon	1	3
Saint Vincent and the Grenadines	2	3
Samoa	2	3
San Marino	2	3
Sao Tome and Principe	3	2
Saudi Arabia	2	3
Senegal	2	1
Serbia	2	3
Serbia and Montenegro	2	3
Seychelles	3	2
Sierra Leone	3	1
Singapore	2.5	2.5
Slovakia	2	3
Slovenia	2	3
Solomon Islands	2	3
Somalia	3	1
South Africa	2	2
Spain	1.5	3
Sri Lanka	2.5	1
Sudan	3	1
Suriname	2.5	2
Swaziland	3	2
Sweden	1	3
Switzerland	1	3
Syrian Arab Republic	2	2
Tajikistan	3	2
Thailand	2.5	1
The former Yugoslav Republic of Macedonia	2	3
Timor-Leste	3	1
Togo	2.5	1
Tokelau	2	2.5
Tonga	2	3
Trinidad and Tobago	2	3
Tunisia	2	2
Turkey	2	2
Turkmenistan	3	2.5
Turks and Caicos Islands	2	3
Tuvalu	2	2.5
Uganda	2	2
Ukraine	2.5	3
United Arab Emirates	2	3
United Kingdom	1	3
United Republic of Tanzania	2	2
United States of America	1	3
United States Virgin Islands	1.5	3
Uruguay	2	3
Uzbekistan	3	2
Vanuatu	2	3
Venezuela (Bolivarian Republic of)	2	2.5
Viet Nam	3	1
Wallis and Futuna Islands	2	3
Western Sahara	2	1
Yemen	2	2
Yugoslav SFR	2	3
Zambia	3	1.5
Zimbabwe	3	1

PL = Pesticide legislation; AP = accessibility of pesticides to farmers

Table S42 Crop-specific pesticide use intensity (PUI)

Activity	PUI
Agave Fibres Nes	1
Alfalfa For Forage+Silag	0
Alfalfa Meal And Pellets	0
Almonds, with shell	2
Anise, badian, fennel, corian.	1
Apples	3
Apricots	3
Arabic Gum	0
Arecanuts	1
Artichokes	2.5
Asparagus	2.5
Avocados	3
Bambara beans	2
Bananas	3
Barley	2
Beans, dry	3
Beans, green	2
Beets For Fodder	0
Berries Nes	2
Blueberries	2
Brazil nuts, with shell	1
Broad beans, horse beans, dry	3
Buckwheat	1
Cabbages and other brassicas	3
Canary seed	1
Carobs	2
Carrots and turnips	3
Cashew nuts, with shell	1.5
Cashewapple	2
Cassava	2.5
Castor oil seed	3
Cauliflowers and broccoli	3
Cereals, most	0
Cereals, nes	2
Cherries	3
Chestnuts	1.5
Chick peas	3
Chicory roots	2.5
Chillies and peppers, dry	2.5
Chillies and peppers, green	2.5
Cinnamon (canella)	2
Citrus fruit, nes	3
Clover For Forage+Silage	0
Cloves	3

Activity	PUI
Cocoa beans	3
Coconuts	2.5
Coffee, green	3
Coir	2
Cow peas, dry	3
Cranberries	1
Cucumbers and gherkins	3
Currants	2
Dates	3
Eggplants (aubergines)	3
Eggs Excl Hen	0
Fibre Crops Nes	3
Figs	2
Flax fibre and tow	2
Fonio	1
Forage Products Nes	0
Fruit Fresh Nes	3
Fruit, tropical fresh nes	3
Fruits, most	0
Garlic	1.5
Ginger	2
Gooseberries	3
Grapefruit (inc. pomelos)	3
Grapes	3
Grass	0
Grasses Nes,Forage+Silag	0
Groundnuts, with shell	2
Gums Natural	3
Hay (Clover,Lucerne,Etc)	0
Hay (Unspecified)	0
Hay Non-Leguminous	0
Hazelnuts, with shell	1
Hemp Tow Waste	2
Hempseed	1
Hops	2
Jojoba	0
Jute	2
Kapok Fruit	2
Karite Nuts (Sheanuts)	1
Kiwi fruit	3
Kolanuts	1
Leguminous Nes,For+Sil	0
Leguminous vegetables, nes	2.5
Lemons and limes	3

Activity	PUI
Lentils	1
Lettuce and chicory	2.5
Linseed	1
Lupins	1
Maize	3
Maize For Forage+Silage	0
Maize, green	3
Mangoes, mangosteens, guavas	3
Manila Fibre (Abaca)	2
Maple	0
Maté	2
Melonseed	1
Millet	2.5
Mixed grain	2
Mules	0
Mushrooms and truffles	0
Mustard seed	1
Natural rubber	3
Nutmeg, mace and cardamoms	2
Nuts, nes	1.5
Oats	1
Oil Of Citronella	0
Oil palm fruit	3
Oils Marine Animals	0
Oilseeds, Nes	2.5
Okra	2
Olives	2
Onions (inc. shallots), green	2
Onions, dry	2
Oranges	3
Other Bastfibres	2
Other melons (inc.cantaloupes)	2.5
Papayas	3
Peaches and nectarines	3
Pears	3
Peas, dry	3
Peas, green	3
Pepper (Piper spp.)	2
Peppermint	1
Persimmons	2
Pigeon peas	2
Pineapples	2.5

Activity	PUI
Pistachios	1
Plantains	3
Plums and sloes	3
Pome Fruit Nes	3
Popcorn	2.5
Poppy seed	1
Potatoes	3
Pulses, nes	2
Pumpkins, squash and gourds	3
Pyrethrum,Dried	0
Quinces	3
Quinoa	1
Ramie	1
Rapeseed	3
Raspberries	3
Rice, paddy	3
Roots and Tubers, most	0
Roots and Tubers, nes	2.5
Rye	1
Safflower seed	2

Activity	PUI
Seed cotton	3
Sesame seed	2
Sisal	2
Sorghum	2.5
Sour cherries	3
Soybeans	3
Spices, nes	2
Spinach	3
Starch and Sugar crops for Alc	0
Stone fruit, nes	3
Straw, Husks	0
Strawberries	3
String beans	3
Sugar beet	2.5
Sugar cane	3
Sugar crops, nes	2.5
Sunflower seed	1
Swedes For Fodder	0
Sweet potatoes	2.5
Tallow tree	0

Activity	PUI
Tangerines, mandarins, clem.	3
Taro (cocoyam)	2.5
Tea	3
Tea Nes	0
Tobacco, unmanufactured	3
Tomatoes	3
Triticale	2
Tung Nuts	1
Turkeys	0
Turnips For Fodder	0
Vanilla	2
Vegetables fresh nes	2.5
Vegetables, most	0
Vegetables+Roots,Fodder	0
Vetches	1
Walnuts, with shell	1.5
Watermelons	3
Wheat	2
Yams	2.5
Yautia (cocoyam)	2.5

PUI = Pesticide use intensity; Nes = other, not elsewhere specified

Table S43 Soil erosion (from water) values in tonnes soil lost/ha*yr

Country	min	Agricultural Land/Cropland mean	max	min	Grassland/ Pasture mean	max	Forest	Orchard	Shrubs	Vineyard	Reference
Albania	0.78		1.86								Grazhdani [65]
Argentina	0.20	18.80	38.00		0.00						Pimentel [66] and Lal, Hall [67] (averages)
Austria	0.50	8.93	39.00								Darmendrail, Cerdan [68] (average) and Strauss and Klaghofer [69] (range)
Belgium	2.80	8.50	17.60								Darmendrail, Cerdan [68] (average) and Verstraeten, Poesen [70] (range)
Benin	17.00		28.00								Lal, Hall [67]
Brazil		18.80									Lal, Hall [67]
Bulgaria	0.27	4.76	5.15	0.03	2.69	6.00		12.65		12.65	Rousseva, Lazarov [71]
Burkina Faso	5.00		35.00								Pimentel [66]
China	10.00		251.00								Lal, Hall [67]
Colombia		22.00									Lal, Hall [67]
Côte d'Ivoire	60.00		570.00								Lal, Hall [67]
Czech Republic	0.00	2.27	13.89								Dostal, Janecek [72]
Denmark	0.26	0.64	12.79		0.03						Darmendrail, Cerdan [68], Veihe and Hasholt [73]
Ecuador	210.00		564.00								Lal, Hall [67]
Ethiopia	8.00		117.70	2.00		29.40					Taddese [74]
Finland	0.10		2.35								Tattari and Rekolainen [75]
France		2.03			0.01					11.09	Darmendrail, Cerdan [68]
Germany		1.32			0.14		0.00		0.13	33.23	Darmendrail, Cerdan [68]. Auerswald [76] (Grassland)
Ghana	5.00		10.00								Lal [77]

Country	min	Agricultural Land/Cropland mean	max	min	Grassland/ Pasture mean	max	Forest	Orchard	Shrubs	Vineyard	Reference
Greece		0.58						0.05	1.17	0.41	Darmendrail, Cerdan [68]
Guatemala	5.00		35.00								Lal, Hall [67]
Guinea	17.90		24.50								Lal, Hall [67]
India		25.00									Ismail and Ravichandran [78]
Indonesia (Java)	50.80		144.30								Magrath and Arens [79]
Italy		1.33			0.28		0.20		0.06	54.86	Darmendrail, Cerdan [68]
Jamaica		90.00									Lal, Hall [67]
Kenya	25.00		45.00		5.00		1.00		7.50		Cohen, Brown [80]
Lesotho		20.00									Bojö [81]
Lithuania	2.50	19.38	32.20		0.01						Darmendrail, Cerdan [68] (average), Jankausas and Fullen [82] (range)
Malawi		20.00									Bojö [81]
Mali		6.50									Bojö [81]
Mexico	10.00		15.00								Margulis [83]
Nepal		40.00									Lal, Hall [67]
Netherlands		6.76									Darmendrail, Cerdan [68]
Nicaragua		11.00									Alfsen, De Franco [84]
Niger	35.00		70.00								Lal, Hall [67]
Nigeria		14.40									Lal, Hall [67]
Norway	0.20		3.50	0.10		2.60					Oygarden, Lundekvam [85]
Papua New Guinea	6.00		320.00								Lal, Hall [67]
Paraguay		18.80									Lal, Hall [67]
Peru		15.00									Lal, Hall [67]

Country	min	Agricultural Land/Cropland mean	max	min	Grassland/ Pasture mean	max	Forest	Orchard	Shrubs	Vineyard	Reference
Portugal		0.59			0.04				0.40		Darmendrail, Cerdan [68]
Romania	0.70		44.80								Ionita, Radoane [86]
Russian Federation	0.50	4.80	20.00								Sidochuk, Litvin [87]
Rwanda	35.00		246.00								Berry, Olson [88]
Senegal	5.00		30.00								Pimentel [66]
Slovakia		20.00									Stankoviansky, Fulajtar [89]
Slovenia	2.39		10.94	0.04		1.89		4.77		22.12	Hrvatin, Komac [90]
South Africa		5.00									Bojö [81]
Spain		0.30			0.84		0.00		0.52		Darmendrail, Cerdan [68]
Switzerland		0.67									Prasuhn [91]
The former Yugoslav Republic of Macedonia	0.04		4.77								Blinkov and Trendafilov [92]
Turkey		2.42									Demirci and Karaburun [93]
Uganda		5.10					0.10				Isabirye [94]
United Kingdom	0.59	2.09	5.60		0.01						Darmendrail, Cerdan [68] _ENREF_12Boardman and Evans [95]
United Republic of Tanzania	10.10		92.80								Lal, Hall [67]
United States of America		6.68									Ismail and Ravichandran [78]
Zimbabwe		43.00									Bojö [81]

References

[1] FAOSTAT. 2013 FAOSTAT database. (Rome, Italy, Food and Agriculture Organization of the United Nations (FAO)).
[2] Gillespie, J.R. & Flanders, F.B. 2010 *Modern Livestock and Poultry Production, 8th edition*.
[3] Gimenez, D.M. 1994 Nutrient requirements of sheep and goats, 6th edition. (National Research Council, http://www.aces.edu/pubs/docs/A/ANR-0812/ANR-0812.html).
[4] Drochner, W., Simon, O. & Jeroch, H. 1999 *Ernährung landwirtschaftlicher Nutztiere*, Ulmer Verlag.
[5] Kirchgessner, M., Windisch, W., Müller, H., Engelhardt, W.v., Leonhard-Marek, S., Breves, G. & Giesecke, D. 1995 Nutritional factors for the quantification of methane production. In *Ruminant physiology: digestion, metabolism, growth and reproduction. Proceedings 8th International Symposium on Ruminant Physiology.* (pp. 333-348, Delmar Publishers).
[6] Schader, C., Jud, K., Meier, M.S., Kuhn, T., Oehen, B. & Gattinger, A. 2014 Quantification of the effectiveness of greenhouse gas mitigation measures in Swiss organic milk production using a life cycle assessment approach. *J. Clean. Prod.* **73**, 227-235.
[7] Courtney, D. 2002 The Feed Value Of Selected Foodstuffs For Beef Cattle. (Department of Environment and Primary Industries, Victoria, Australia).
[8] Hay & Straw. 2013 The hay & Straw Company - Product information. (New Zealand).
[9] LGAN. 2012 An Introduction to Feeding Nutritionally Superior Maize Silage. (Rothwell, UK, Limagrain Ltd).
[10] Schaumann. 2010 Maize Silage Handbook.
[11] Feedipedia. 2013 Feedipedia. http://www.feedipedia.org/. *INRA CIRAD AFZ and FAO*.
[12] Alexandratos, N. & Bruinsma, J. 2012 World Agriculture Towards 2030/2050. The 2012 Revision. In *ESA Working Paper No. 12-03* (ed. Agricultural Development Economics Division). Rome, FAO).
[13] Golan, A., Judge, G. & Miller, D. 1996 *Maximum entropy econometrics. Robust estimation with limited data*. Chichester, New York, Wiley.
[14] IPCC. 2006 2006 IPCC Guidelines for National Greenhouse Gas Inventories - Volume 4: Agriculture, Forestry and Other Land Use. (Intergovernmental Panel on Climate Change (IPCC)).
[15] Kirchgeßner, M. 1995 *Tierernährung*. Frankfurt/Main, DLG-Verlag.
[16] Neuweiler, R. 2011 Düngungsrichtlinien für den Gemüsebau. (Agroscope).
[17] IFA. 2013 World Fertilizer Use Manual (International Fertilizer Association IFA).
[18] Smil, V. 1999 Crop residues: agriculture's largest harvest. *Bioscience* **49**, 299-308.
[19] Bertschinger, L., Gysi, C., Häseli, A., Neuwiler, R., Pfamatter, W., Ryser, J., Schmid, A. & Weibel, F. 2003 Grundlagen für die Düngung der Obstkulturen. (Agroscope).
[20] Herridge, D., Peoples, M. & Boddey, R. 2008 Global inputs of biological nitrogen fixation in agricultural systems. *Plant Soil* **311**, 1-18. (doi:10.1007/s11104-008-9668-3).
[21] Akbar, N., Iqbal, A., Khan, H.Z., Hanif, M.K. & Bashir, M.U. 2010 Effect of different sowing dates on the yield and yield components of direct seeded fine rice (Oryza sativa L.). *J. Plant Breeding Crop Sci,* **2**, 312-315.
[22] Hasanuzzaman, M., Karim, F., Fattah, Q.A. & Nahar, K. 2007 Yield Performance of Chickpea Varieties Following Application of Growth Regulator. *Am. Eurasian J. Sci. Res.* **2**, 117-120.
[23] Agri-Facts. 2007 Using 1000 Kernel Weight for Calculating Seeding Rates and Harvest Losses. (Alberta Agriculture and Food).
[24] Zare, M. 2011 Interrelationship between grain yield and related traits in rapeseed (Brassica napus L.). *Afr. J. Agric. Res.* **6**, 6684-6689.
[25] Shirokle, S.S., Kenghe, R.N. & Nimkar, P.M. 2011 Moisture Dependent Physical Properties of Soybean *Int. J. Eng. Sci. Technol.* **3**, 3807-3815.
[26] Karadogan, T., Akgun, I. & Altindal, N. 2009 Effect of Leaf Removal on Sunflower Yield and Yield Components and Some Quality Characters. *Turk. J. Field Crops* **14**, 45-54.
[27] FAO. 2013 AQUASTAT Database. http://www.fao.org/nr/water/aquastat/main/index.stm. (Rome, Italy, Food and Agriculture Organization of the United Nations (FAO)).
[28] Nemecek, T., Dubois, D., Huguenin-Elie, O. & Gaillard, G. 2011 Life cycle assessment of Swiss farming systems: I. Integrated and organic farming. *Agric. Syst.* **104**, 217-232.

[29] Nemecek, T. & Kägi, T. 2007 Life Cycle Inventories of Agricultural Production Systems. Data v2.0. Ecoinvent report No. 15. (Zürich and Dübendorf, ecoinvent centre. Swiss Centre for Life Cycle Inventories. Agroscope Reckenholz-Tänikon Research Station (ART)).
[30] Schader, C. 2009 *Cost-effectiveness of organic farming for achieving environmental policy targets in Switzerland*. Aberystwyth, UK; Frick, Switzerland, Aberystwyth University; Research Institute of Organic Farming (FiBL).
[31] Frischknecht, R., Althaus, H.-J., Doka, G., Dones, R., Heck, T., Hellweg, S., Hischier, R., Jungbluth, N., Nemecek, T., Reblitzer, G., et al. 2007 Overview and methodology. Final report ecoinvent v2.0 No.1. (Duebendorf, Switzerland, Swiss Centre for Life Cycle Inventories).
[32] Erb, K.H., Gaube, V., Krausmann, F., Plutzar, C., Bondeau, A. & Haberl, H. 2007 A comprehensive global 5 min resolution land-use data set for the year 2000 consistent with national census data. *J. Land Use Sci.* **2**, 191-224.
[33] Herrero, M., Havlik, P., Valin, H., Notenbaert, A., Rufino, M.C., Thornton, P.K., Blümmel, M., Weiss, F., Grace, D. & Obersteiner, M. 2013 Biomass use, production, feed efficiencies, and greenhouse gas emissions from global livestock systems. *Proc Natl Acad Sci U S A* **110**, 20888-20893.
[34] ACW & ART. 2009 Grundlagen für die Düngung im Acker- und Futterbau (GRUDAF). *Agrarforschung* **16**, 1-95.
[35] ASAE. 2005 Manure Production and Characteristics. (American Society of Agricultural Engineers).
[36] ADAS. 2007 Nitrogen output of livestock excreta. (ADAS).
[37] Flachowsky, G. & Brade, W. 2007 Potenziale zur Reduzierung der Methan-Emissionen bei Wiederkäuern. *Züchtungskunde* **79**, 417-465.
[38] DLG. 2009 Empfehlungen zur Fütterung von Mutterkühen und deren Nachzucht. (DLG Fütterungsempfehlungen).
[39] Steinfeld, H. 2006 *Livestock's long shadow: environmental issues and options*. Rome, Food and Agriculture Organization of the United Nations.
[40] USEPA. 2004 National Emission Inventory—Ammonia Emissions from Animal Husbandry Operations. (United States Environmental Protection Agency US EPA).
[41] IPCC. 1997 Revised 1996 IPCC Guidelines for National Greenhouse Gas Inventories. (Intergovernmental Panel on Climate Change (IPCC)).
[42] De Wit, J., van Keulen, H., van der Meer, H.G. & Nell, A.J. 1997 Animal manure: asset or liability? . *World Anim. Rev.* **88**, 30-37.
[43] Hutchings, N.J., Sommer, S.G., Andersen, J.M. & Asman, W.A.H. 2001 A detailed ammonia emission inventory for Denmark. *Atmos. Environ.* **35**, 1959-1968.
[44] EEA. 2002 Joint EMEP/CORINAIR Atmospheric Emission Inventory Guidebook, 3rd ed. (European Environmental Agency EEA).
[45] FAO. 2001 Food Balance Sheets - A Handbook. (Rome, Food and Agriculture Organization of the United Nations FAO).
[46] FAO. 2000 Technical ConversionFactors for Agricultural Commodities. (Rome, Food and Agriculture Organization of the United Nations FAO).
[47] FAO. 2011 Demand and Supply of Feed ingrdients for farmed fish and crustaceans, FAO Fisheries and Aquaculture Technical Paper 564. (Rome, Food and Agriculture Organization of the United Nations FAO).
[48] FAO. 2012 The State of World Fisheries and Aquaculture 2012. (Rome, Food and Agriculture Organization of the United Nations FAO).
[49] FAO. 2010 FAO Fishery and Aquaculture Statistics 2010. (Rome, Food and Agriculture Organization of the United Nations FAO).
[50] OECD/FAO. 2012 OECD-FAO Agricultural Outlook 2012. (OECD, FAO).
[51] UN. 2008 *World population prospects : the 2008 revision: United Nations world population estimates and projections*. New York, United Nations; 3 v. p.
[52] FAO. 2013 Food Security Indicators. (Rome, Italy, Food and Agriculture Organization FAO).
[53] FNB. 2002 *Dietary Reference Intake for Energy, Carbohydrate, Fiber, Fat, Fatty Acids, Cholesterol, Protein, and Amino Acids*, Food and Nutrition Board of Institute of Medicine of U.S. National Academy of Sciences

[54] Müller, C., Bondeau, A., Popp, A., Waha, K. & Fader, M. 2010 Climate Change Impacts on Agricultural Yields. In *World Development Report 2010, Background Note* (Potsdam Institute for Climate Impact Research PIK).
[55] Challinor, A., Watson, J., Lobell, D., Howden, S., Smith, D. & Chhetri, N. 2014 A meta-analysis of crop yield under climate change and adaptation. *Nat. Clim. Change* **4**, 287-291.
[56] Porter, J.R., Xie, L., Challinor, A.J., Cochrane, K., Howden, S.M., Iqbal, M.M., Lobell, D.B. & Travasso, M.I. 2014 Chapter 7: Food Security and Food Production Systems. In *Climate Change 2014, Contribution of the WG II to the 5th Assessment Report of the IPCC* (Cambridge University Press).
[57] Müller, C. & Robertson, R.D. 2014 Projecting future crop productivity for global economic modeling. *Agr. Econ.* **45**, 37-50.
[58] Lobell, D., Schlenker, W. & Costa-Roberts, J. 2011 Climate Trends and Global Crop Production Since 1980. *Science* **333**, 616-620.
[59] Asseng, S., Ewert, F., Martre, P., Rötter, R., Lobell, D., Cammarano, D., Kimball, B., Ottman, M., Wall, G. & White, J. 2014 Rising temperatures reduce global wheat production. *Nat. Clim. Change* **5**, 143-147.
[60] GfE. 2006 Recommendations for energy and nutrient supply for Pigs. (Frankfut/Main, Gesellschaft für Ernährungsphysiologie).
[61] GfE. 1995 Nutrient requirements of beef cattle. (Frankfut/Main, Gesellschaft für Ernährungsphysiologie).
[62] GfE. 1996 Energy requirements of sheep. *Proc. Soc. Nutr. Physiol.* **5**, 149-152.
[63] NRC. 1994 Nutrient requirements of poultry. (National Research Council NRC, Board on Agriculture, Committee on Animal Nutrition, Subcommittee on Poultry Nutrition).
[64] NRC. 1996 Nutrient requirements of domestic animals. (National Research Council NRC, Board on Agriculture, Committee on Animal Nutrition, Subcommittee on Beef Cattle Nutrition).
[65] Grazhdani, S. 2006 Albania. In *Soil Erosion in Europe* (eds. J. Boardman & J. Poesen). West Sussex, England, John Wiley & Sons, Ltd).
[66] Pimentel, D., ed. 1993 *World Soil Erosion and Conservation*, Cambridge University Press.
[67] Lal, R., Hall, G.F. & Miller, F.P. 1989 Soil Degradaton: 1. Basic Processes. *Land Degradation & Rehabilitaion* **1**, 51-69.
[68] Darmendrail, D., Cerdan, O., Gobin A., Bouzit M., Blanchard F. & B., S. 2004 Assessing the economic impact of soil deterioration: Case Studies and Database Research. (European Commission).
[69] Strauss, P. & Klaghofer, E. 2006 Austria. In *Soil Erosion in Europe* (eds. J. Boardman & J. Poesen). West Sussex, England, John Wiley & Sons, Ltd).
[70] Verstraeten, G., Poesen, J., Goossens, D., Gillijns, K., Bielders, C., Gabriels, D., Ruysschaert, G., Van Den Eeckhaut, M., Vanwalleghem, T. & Govers, G. 2006 Belgium. In *Soil Erosion in Europe* (eds. J. Boardman & J. Poesen), pp. 385-411, John Wiley & Sons, Ltd).
[71] Rousseva, S., Lazarov, A., Tsvetkova, E., Marinov, I., Malinov, I., Kroumov, V. & Stefanova, V. 2006 Bulgaria. In *Soil Erosion in Europe* (eds. J. Boardman & J. Poesen). West Sussex, England, John Wiley & Sons, Ltd).
[72] Dostal, T., Janecek, M., Kliment, Z., Krasa, J., Langhammer, J., Vaska, J. & Vrana, K. 2006 Czech Republic. In *Soil Erosion in Europe* (eds. J. Boardman & J. Poesen). West Sussex, England, John Wiley & Sons, Ltd).
[73] Veihe, A. & Hasholt, B. 2006 Denmark. In *Soil Erosion in Europe* (eds. J. Boardman & J. Poesen). West Sussex, England, John Wiley & Sons, Ltd).
[74] Taddese, G. 2001 Land Degradation: A Challenge to Ethiopia. *Environ. Manage.* **27**, 815-824. (doi:10.1007/s002670010190).
[75] Tattari, S. & Rekolainen, S. 2006 Finland. In *Soil Erosion in Europe* (eds. J. Boardman & J. Poesen). West Sussex, England, John Wiley & Sons, Ltd).
[76] Auerswald, K. 2006 Germany. In *Soil Erosion in Europe* (eds. J. Boardman & J. Poesen), pp. 213-230, John Wiley & Sons, Ltd).
[77] Lal, R. 1993 Soil erosion and conservation in West Africa. In *World Soil Erosion and Conservation* (eds. D. Pimentel &), Cambridge University Press).
[78] Ismail, J. & Ravichandran, S. 2008 RUSLE2 Model Application for Soil Erosion Assessment Using Remote Sensing and GIS. *Water Resour. Manage.* **22**, 83-102. (doi:10.1007/s11269-006-9145-9).

[79] Magrath, W. & Arens, P. 1989 The costs of soil erosion on Java: a natural resource accounting approach. In *Environment Department working paper ; no. ENV 18* (Washington D.C. , The Worldbank).
[80] Cohen, M.J., Brown, M.T. & Shepherd, K.D. 2006 Estimating the environmental costs of soil erosion at multiple scales in Kenya using emergy synthesis. *Agric., Ecosyst. Environ.* **114**, 249-269. (doi:http://dx.doi.org/10.1016/j.agee.2005.10.021).
[81] Bojö, J. 1996 Analysis- The costs of land degradation in Sub-Saharan Africa. *Ecol. Econ.*, 161-173.
[82] Jankausas, B. & Fullen, M.A. 2006 Lithuania. In *Soil Erosion in Europe* (eds. J. Boardman & J. Poesen). West Sussex, England, John Wiley & Sons, Ltd).
[83] Margulis, S. 1992 Back of the envelope estimates of environmental damage costs in Mexico. *Policy Research Working Paper Series*.
[84] Alfsen, K.H., De Franco, M.A., Glomsrød, S. & Johnsen, T. 1996 The cost of soil erosion in Nicaragua. *Ecol. Econ.* **16**, 129-145. (doi:http://dx.doi.org/10.1016/0921-8009(95)00083-6).
[85] Oygarden, L., Lundekvam, H., Arnoldussen, A.H. & Borresen, T. 2006 Norway. In *Soil Erosion in Europe* (eds. J. Boardman & J. Poesen). West Sussex, England, John Wiley & Sons, Ltd).
[86] Ionita, I., Radoane, M. & Mircea, S. 2006 Romania. In *Soil Erosion in Europe* (eds. J. Boardman & J. Poesen). West Sussex, England, John Wiley & Sons, Ltd).
[87] Sidochuk, A., Litvin, L., Golosv, V. & Chernysh, A. 2006 European Russia and Byelorus. In *Soil Erosion in Europe* (eds. J. Boardman & J. Poesen). West Sussex, England, John Wiley and Sons, Ltd.).
[88] Berry, L., Olson, J. & Campbell, D. 2003 Assessing the extent, cost and impact of land degradation at the national level: findings and lessons learned from seven pilot case studies. (Commissioned by Global Mechanism with support from the World BankWorld Bank).
[89] Stankoviansky, M., Fulajtar, E. & Jambor, P. 2006 Slovakia. In *Soil Erosion in Europe* (eds. J. Boardman & J. Poesen). West Sussex, England, John Wiley & Sons, Ltd).
[90] Hrvatin, M., Komac, B., Perko, D. & Zorn, M. 2006 Slovenia. In *Soil Erosion in Europe* (eds. J. Boardman & J. Poesen). West Sussex, England, John Wiley and Sons, Ltd).
[91] Prasuhn, V. 2004 Mapping of actual soil erosion in Switzerland. (Agroscope Reckenholz, Zürich-Reckenholz, Switzerland, Swiss Federal Research Station for Agroecology and Agriculture).
[92] Blinkov, I. & Trendafilov, A. 2006 Macedonia. In *Soil Erosion in Europe* (eds. J. Boardman & J. Poesen). West Sussex, England, John Wiley and Sons, Ltd).
[93] Demirci, A. & Karaburun, A. 2012 Estimation of soil erosion using RUSLE in a GIS framework: a case study in the Buyukcekmece Lake watershed, northwest Turkey. *Environ Earth Sci* **66**, 903-913. (doi:10.1007/s12665-011-1300-9).
[94] Isabirye, M. 2005 *Land Evaluation Around Lake Victoria: Environmental Implications of Land Use Change*, Katholieke Universiteit Leuven.
[95] Boardman, J. & Evans, R. 2006 Britain. In *Soil Erosion in Europe* (eds. J. Boardman & J. Poesen). West Sussex, England, John Wiley & Sons, Ltd).
